# Structural evolution of the MTCH family of mitochondrial insertases

**DOI:** 10.64898/2026.02.17.705849

**Published:** 2026-02-18

**Authors:** Taylor A. Stevens, Zhilin Luo, Camryn Lee, Masami Hazu, Erini G. Galatis, Alison J. Inglis, Alina Guna, Rebecca M. Voorhees

**Affiliations:** 1 Division of Biology and Biological Engineering, California Institute of Technology, 1200 E. California Ave., Pasadena, CA 91125, USA; 2 Howard Hughes Medical Institute, California Institute of Technology, 1200 E. California Ave., Pasadena, CA 91125, USA; 3 Department of Biochemistry and Biophysics, University of California, San Francisco, San Francisco, CA, USA.

## Abstract

Here we demonstrated that MTCH2 is the defining member of a large family of mitochondrial outer membrane (OM) insertases. The MTCH family is conserved across holozoa and has diverged from the solute carrier 25 transporters. The cryoelectron microscopy structure of the 33 kDa human MTCH2 revealed that evolution of its insertase activity required loss of a transmembrane helix, which created a lipid-accessible hydrophilic groove stabilized by its unique, structured C-terminus. Mutational analyses showed that MTCH insertase activity is attenuated, while experimental structures of hyperactive mutants demonstrated that the hydrophobicity, charge, and size of the residues that line its groove regulated MTCH function. Leveraging the MTCH2 structure, we identified the plant OM insertase, and proposed a universal mechanism for OM insertion across all kingdoms of life.

## Background and Introduction

A critical step in the biogenesis of all integral membrane proteins is their integration into the lipid bilayer. Successful insertion requires the simultaneous shepherding of a hydrophobic element, often a transmembrane helix (TM), into the membrane coupled with the translocation of an associated soluble domain across the bilayer. The latter of which typically requires catalysis by a membrane protein insertase. To ensure the efficient and robust insertion of the full diversity of membrane proteins, eukaryotic cells have evolved multiple families of insertases that function at each membrane and specialize in specific classes of substrates (1–3).

An important site of membrane protein biogenesis is the mitochondrial outer membrane (OM). The OM is essential for mediating communication between the organelle and the cytosol (4), which relies on the accurate localization and insertion of a suite of α-helical membrane proteins. Because of their roles in regulating both mitochondrial dynamics and cellular processes such as apoptosis and the innate immune system, defects in OM protein biogenesis are associated with numerous human diseases including Parkinson’s disease, metabolic disorders, and many cancers (5, 6). In mammals, the OM contains ~150 α-helical membrane proteins, all of which are encoded by the nuclear genome, translated on cytosolic ribosomes, and then integrated directly into the OM (7).

We recently demonstrated that the human Mitochondrial Carrier Homolog 2 (MTCH2) functions as an OM membrane insertase for many of these α-helical proteins in human cells (8), providing a biochemical explanation for its central role in mitochondrial biology and human disease (9–11). MTCH2 and its close paralog, MTCH1 (12), are diverged members of the solute carrier 25 (SLC25) family, which are transporters typically localized to the inner mitochondrial membrane (13–15). Many aspects of MTCH2 function are not understood, including disagreement about its topology, fundamental questions regarding the molecular basis of its insertase activity, and limited information about how broadly it is conserved. Moreover, how MTCH2 evolved from a family of transporters that move small molecules across the membrane to one that mediates insertion of proteins into the bilayer is not clear. Finally, the relationship between MTCH2 and its counterparts in fungus (Mim1/2; (16, 17)) and protists (pATOM36; (18, 19)), and identification of a putative OM insertase in plants, would provide insight into the evolution of OM protein biogenesis across eukaryotic kingdoms. Here we set out to use a combination of structure, biochemistry, and molecular biology to characterize how MTCH2 evolved to co-opt a solute carrier fold for insertion of α-helical proteins into the bilayer.

### The MTCH family of mitochondrial membrane protein insertases

**Fig. 1.**
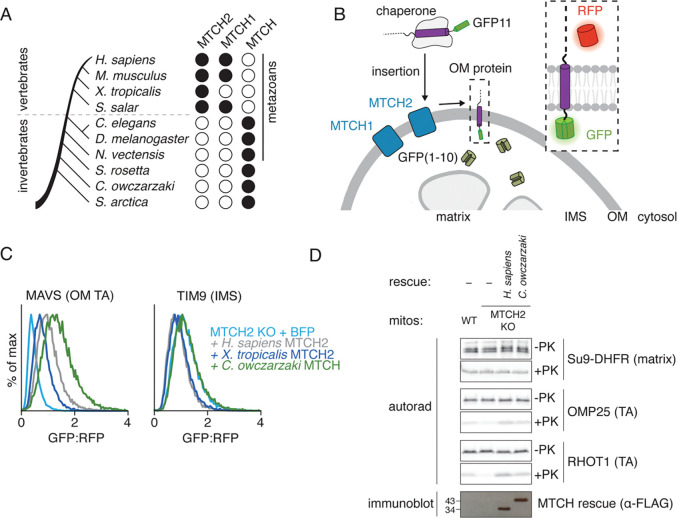
MTCH2 is the defining member of the MTCH family of insertases conserved across holozoa. **A)** To identify MTCH2 homologs across the holozoan clade, we queried Uniprot for sequences annotated with the panther family PTHR10780. Vertebrates expressed homologs of both MTCH1 and MTCH2, while invertebrates contained a single MTCH homolog, which cannot be unambiguously assigned as related to MTCH1 vs 2. The presence and type of the predicted homolog is indicated with a black circle, and a summary of the query results are in table S4. **B)** Schematic of the split-GFP fluorescent reporter system previously established to assess insertion into the outer mitochondrial membrane (OM) (8, 21, 22). Here, GFP(1–10) is localized to the inner membrane space (IMS) while GFP11 is appended to the protein of interest. Insertion into the OM in the correct orientation results in complementation and thereby GFP fluorescence. GFP11 containing reporters are encoded on the same open reading frame as a normalization marker, RFP, separated by a P2A ribosomal skipping sequence to specifically allow measurement of post-translational effects on reporter biogenesis. Using this system, we demonstrated that both MTCH1 and MTCH2 facilitated insertion of α-helical containing OM proteins in human cells (see also fig. S2). **C)** Insertion of the OM tail-anchored (TA) reporter MAVS-GFP11 and an IMS localized control, TIM9-GFP11 as described in (B) was assessed by flow cytometry in K562 MTCH2 KO cells expressing either a BFP control, human MTCH2 (*H. sapiens*), *X. tropicalis* MTCH2, or *C. owczarzaki* MTCH. GFP fluorescence, a proxy for reporter insertion, relative to our normalization control (RFP) was calculated and is displayed as a histogram (additional substrates and relevant controls are displayed in fig S3A). **D)** To directly assess insertion activity of the indicated MTCH homologs, we performed an *in vitro* insertion experiment using isolated mitochondria (fig. S3C). The indicated ^35^S-methionine-labelled substrates were translated in rabbit reticulocyte lysate and released from the ribosome by treatment with puromycin. These included the MTCH2 independent Su9-DHFR control (which contains the canonical TOM targeting sequence, Su9), and two MTCH2 dependent OM proteins, OMP25 (the native sequence) and RHOT1 (where the large cytosolic lumenal domain of RHOT1 is replaced with the smaller, globular VHP domain as described previously (8)). Each substrate was incubated with mitochondria isolated from WT or MTCH2 KO human K562 cells expressing either a mock control, 3xFLAG-tagged *H. sapiens* MTCH2, or 3xFLAG-tagged *C. owczarzaki* MTCH. Relative insertion was assessed using a protease protection assay and analyzed using SDS-polyacrylamide gel electrophoresis (SDS-PAGE) and autoradiography, before (−PK) and after (+PK) addition of protease. An immunoblot (3xFLAG) is displayed to indicate relative expression of the MTCH homologs.

Bioinformatic analysis identified a distinct SLC25 subclass, which includes both human MTCH1 and MTCH2 as well as thousands of other sequences widely distributed across the holozoan clade (Fig. 1A). Sequence comparison suggested that these MTCH2 homologs retain many features of the SLC25 family including highly conserved prolines within TM1, TM3, and TM5 (fig. S1A,B) (13). However, they have also lost several transporter elements such as the characteristic salt bridges between helices, important for the alternating access mechanism used for solute transport. Further, the MTCH2 homologs all contain a highly diverged and shortened C-terminus compared to canonical SLC25s (fig. S1C). While all metazoans contain at least one MTCH2 homolog predicted to localize to their respective OM, vertebrates express two paralogs, MTCH1 and MTCH2, which have previously been shown to be synthetic lethal in human cells (20).

To determine if these putative MTCH2 homologs could also function as membrane protein insertases, we tested if they could rescue α-helical OM protein insertion in a MTCH2 depleted human cell. For this, we used a split-GFP reporter assay that specifically monitors OM insertion (8, 21, 22) (Fig. 1B). Consistent with earlier results (8, 12), we observed that MTCH1 could rescue loss of MTCH2 for several α-helical OM substrates (fig. S2A,B). Mammalian mitochondria also express a third SLC25 member, SLC25A46, in their outer membrane (15), which does not appear to play a role in insertion (fig. S2C,D). Remarkably, we found that several MTCH2 homologs, including those from *X. tropicalis* and the single-celled organism *C. owczarzaki*, rescued a MTCH2 deletion phenotype using our ratiometric fluorescent reporter assay (Fig. 1C, fig. S3A,B). To confirm that this effect was at the level of insertion, we purified human mitochondria from a MTCH2 knockout cell line exogenously expressing either human MTCH2 or the *C. owczarzaki* MTCH2 homolog. Even the distantly related homolog was able to mediate the integration of human α-helical proteins into the OM in vitro (Fig. 1D, fig. S3C), suggesting an exceptional level of conservation across what we will now refer to as the MTCH family of membrane protein insertases.

### The structure and evolution of human MTCH2

To better understand how this family of proteins may have evolved their insertase activity from an ancestral transporter fold, we sought to analyze the structure of a representative member of the MTCH family. While the arrangement of some of the transmembrane helices could be potentially inferred due to their similarity to the SLC25s, the conserved C-terminus, unique to the MTCH family, and likely of functional significance, cannot be confidently predicted by AlphaFold (23, 24). Additionally, while canonical SLC25 carriers alternate between conformations open to the cytosol or matrix (25, 26), MTCH2, and all other MTCH homologs, lack conserved residues stabilizing either of these conformations. It is not clear whether MTCH2 adopts multiple conformations, and indeed there is disagreement in the literature about its topology and number of TMs. Therefore, an experimental structure is required to unambiguously assess the molecular basis for insertase activity. For structural analysis we chose to focus on human MTCH2, since it is the best characterized member of the family and because an experimental structure could be therapeutically valuable due to the close association between MTCH2 and human disease (27).

**Fig. 2.**
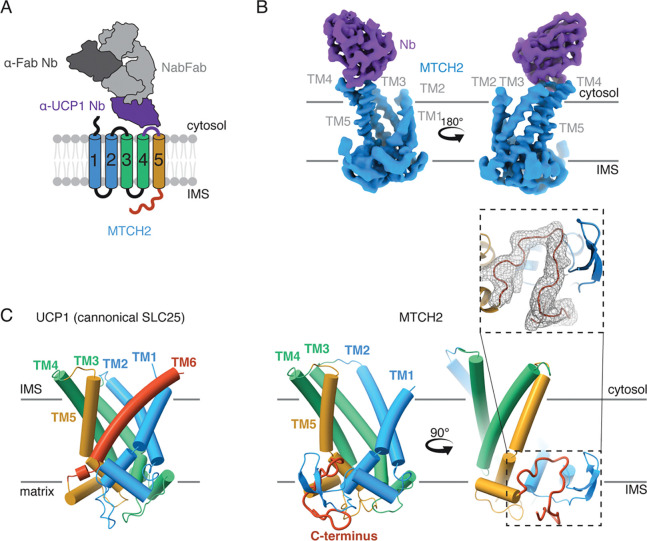
Structure of the 33 kDa human MTCH2 determined by single particle cryo-EM. **A)** Schematic of the strategy used to assemble the tetrameric complexes of human MTCH2 used for structural determination. As described in detail in Fig. S5C,D, the *H. sapiens* MTCH2 sequence was modified to include an epitope that is recognized by the α-UCP1 nanobody (Nb) pMb65 (29). pMb65 was modified to permit binding of the universal NabFab (30), which was itself bound to an additional α-Fab Nb (31). **B)** EM density map of human MTCH2 (blue) and the α-UCP1 Nb (purple) determined to an overall resolution of 3.6 Å. All five TMs were unambiguously resolved and the resolution was sufficient to allow *de novo* building of the C-terminal domain of MTCH2, which is unique to the MTCH insertase family (fig. S1C). **C)** Comparison of MTCH2 with the SLC25 transporter UCP1 (PDB ID: 8HBV, left (55)) highlights both the similarities and differences between the MTCH family insertases and their ancestral transporters, including the loss of a 6^th^ TM. Inset is the refined model and EM density for the C-terminus of MTCH2, whose structure was incorrectly predicted by AlphaFold3. Its positioning directly below the cavity left by loss of the 6^th^ TM, and role in stabilization of MTCH2 (fig. S8D,E) suggests it may have co-evolved with the insertase activity of the MTCH family.

Due to its small size (33 kDa) structural determination by cryoelectron (cryo-EM) microscopy of MTCH2 required a strategy to rigidly add asymmetric mass as a fiducial marker for particle alignment. We tested several fusion strategies at various positions within MTCH2. These included fusions with BRIL, which has been successfully used to determine other membrane protein structures and can be complexed with the established anti-BRIL antibody (28). While we were able to determine an initial low-resolution structure using a BRIL fusion strategy (fig. S4, Table S1), preliminary density maps suggested that further optimization would be required to allow *de novo* building of the C-terminal domain of MTCH2. We instead leveraged tools from the recently determined structure of the uncoupling protein (UCP1) (29), an SLC25 transporter localized to the inner mitochondrial membrane. Jones et al. generated a nanobody that binds to a short segment of the loop between TMs 4 and 5 (loop 4/5), which is positioned in the IMS. We determined that the homologous cytosolic loop in MTCH2 was a similar length, its sequence was poorly conserved amongst MTCH homologs, and mutations to this loop did not affect MTCH2 insertase activity in human cells (fig. S5A,B). We therefore reasoned that we could replace these residues in MTCH2 with the nanobody recognition sequence from the UCP1 loop4/5 without altering MTCH2’s structure or function (fig. S5C,D). Indeed the MTCH2-loop fusion was stably expressed, remained competent for substrate insertion (fig. S5E,F), and could be purified as a homogeneous and monodispersed sample in the detergent UDM. We then generated a version of the UCP1 nanobody that could be recognized by a universal anti-nanobody Fab (NabFab) (30) to allow purification of a 112 kDa tetrameric complex of MTCH2, the modified UCP1 nanobody, the NabFab, and finally an anti-Fab nanobody (31) (Fig. 2A).

Using a standard data collection and computational processing pipeline, we determined the structure of human MTCH2 to an overall resolution of 3.6 Å (Fig. 2B, fig. S6-7, Table S1), sufficient to allow unambiguous fitting of the TM helices and *de novo* building of the C-terminal domain (Fig. 2C) (23, 24). Comparison of the EM density from the UCP1-fused and BRIL-fused datasets suggests that the overall shape and conformation is not impacted by either modification (fig. S7C,D). Given that the size of loop 4/5 is similar in most SLC25s, but the sequence is typically not critical for function (32), it is likely that this UCP1-fusion strategy is universally applicable to enable structure determination of diverse members of this essential transporter family and all MTCH insertases.

**Fig. 3.**
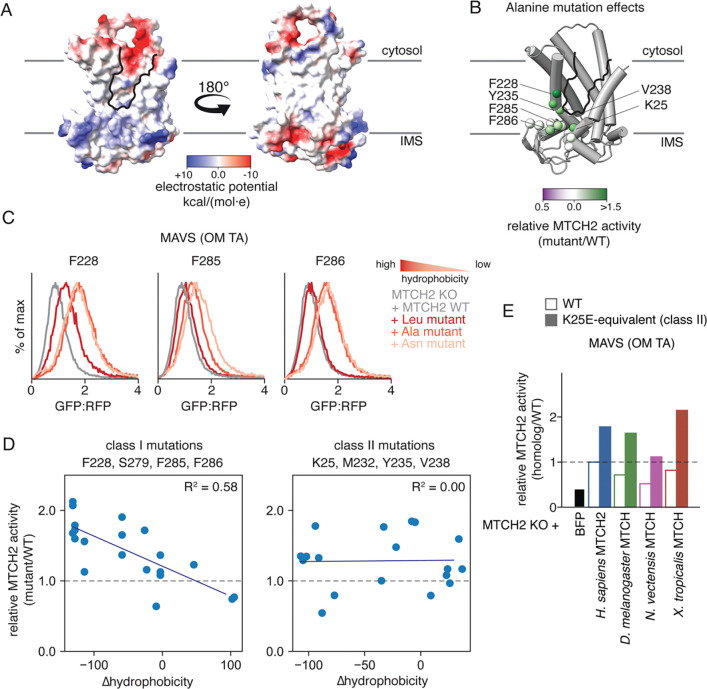
The activity of MTCH2 is attenuated by residues surrounding its hydrophilic groove. **A)** Coulombic electrostatic potential was calculated using ChimeraX and mapped onto a surface visualization of the experimentally determined model of MTCH2. **B)** To test the role of MTCH2’s hydrophilic groove in insertion (outlined in black), we performed alanine scanning mutagenesis on the indicated residues surrounding the groove. The ability of each of these alanine mutants to rescue a MTCH2 KO phenotype on MAVS insertion, when compared to similar expression of WT MTCH2 (additional substrates and relevant controls are displayed in fig. S11B), was assessed using the ratiometric reporter described in Fig 1B. The relative activity of each alanine mutant compared to WT MTCH2 (calculated as GFP:RFP_mut_/GFP:RFP_wt_) was mapped onto the corresponding site using the indicated scale. Darker green indicates positions where mutations leads to increased insertion compared to wild type MTCH2 that cannot be explained by changes in expression (fig. S11C). **C)** Eight positions lining the MTCH2 hydrophilic groove were selected for more extensive mutagenesis (Phe, Leu, Gln, Glu and Arg in addition to Ala). Relative insertion of MAVS and OMP25 and a MTCH2 independent control was assessed as in (B) (additional substrates and relevant controls are displayed in fig. S13). Highlighted here are the effects of a subset of mutations for three representative positions lining the hydrophilic groove. Curves are colored by the hydrophobicity of the amino acid introduced in each mutant. **D)** (left) Data summarizing the effect of mutations (Phe, Leu, Ala, Gln, Glu, Arg) at four MTCH2 positions (F228, S279, F285, and F286) on MAVS insertion as described in (C). Displayed is the relative MTCH2 activity (GFP:RFP_mut_/GFP:RFP_wt_) plotted against the change in hydrophobicity of the mutated amino acid (ΔHydrophobicity=hydrophobicity_aa mut_-hydrophobicity_aa WT_) (56). These positions, referred to as Class I, share a correlation between the hydrophobicity of the mutation and activity of MTCH2 in insertion. (right) The same analysis was carried out with four separate positions (K25E, M232, Y235, V238), referred to as Class II, where there is no strong correlation between hyperactivity and hydrophobicity (additional substrate displayed in fig. S14A). **E)** To test whether MTCH2 activating mutations are conserved across the MTCH family, we measured the activity of a panel of MTCH homologs and their respective Class II mutants. Using the same rescue assay described in Fig. 1C, we tested the relative activity of a BFP control, WT and K25E human MTCH2, and WT and mutant MTCH homologs from *C. owczarzaki*, *D. melanogaster*, or *N. vectensis* (mutations equivalent to K25E in human MTCH2) on MAVS insertion. Relative activity was calculated as GFP:RFP_mut_/GFP:RFP_wt_ (additional substrates and relevant controls are displayed in fig. S15A).

Comparison with the UCP1 structure definitively indicated that unlike a canonical SLC25, MTCH2 contained only five TMs (Fig. 2C). This is consistent with our analysis of its topology in human cells (fig. S8A,B) and resolves earlier disagreement in the literature. Instead, the C-terminus of MTCH2, conserved across the MTCH family of insertases (fig. S1A), adopts a defined fold that stabilizes the cavity formed by loss of this sixth TM. Indeed, truncations beyond M278 to the C-terminus of MTCH2 resulted in a marked destabilization of the protein in cells (fig. S8D,E). Therefore, unlike a transporter, which forms an enclosed hydrophilic pore through the membrane, MTCH2 contains an exposed hydrophilic vestibule within the bilayer (Fig. 3A). Further analysis revealed that the structure of MTCH2 adopts a cytoplasm-open conformation (fig. S9A) despite lacking conserved salt bridges, which stabilize this conformation in canonical SLC25 carriers (fig. S1B), and mutations restoring these salt bridges do not reduce MTCH2s function (fig. S9B). The presence of a cytosol-accessible cavity with a lateral opening to the lipid bilayer is consistent with a model in which nascent TMs are trafficked from the cytosol to MTCH2 for OM insertion. Notably, the cavity is also lined by a series of bulky hydrophobic residues that are conserved across MTCH family homologs, many of which are within the C-terminal domain that could only be visualized in the experimental structure (fig. S1C and S10A). The MTCH family of insertases is yet another example of the convergent evolution that has led many evolutionarily unrelated insertase families to utilize similar hydrophilic grooves to mediate insertion (fig. S10A). This includes the universally conserved Oxa1/YidC superfamily of insertases (33, 34) including EMC (35), GET1/2 (36), and YidC (37) (fig. S10B). In all cases, positioning of a hydrophilic surface within the membrane is thought to decrease the energetic barrier of translocation of soluble domains across the hydrophobic core of the bilayer, thereby catalyzing membrane protein insertion (1).

Certainly, loss of this sixth TM is one of the most striking evolutionary adaptations between the SLC25 transporters and the MTCH insertase family. Indeed, SLC25A46, though also localized to the OM like MTCH1 and 2, cannot mediate membrane protein insertion (fig. S2A,C) and is predicted to contain six TMs (23, 24). To test whether this evolved hydrophilic vestibule was important for MTCH family insertase function, we performed alanine scanning mutagenesis to residues around this region (Fig. 3B, fig. S11A,B). While we expected that mutations would primarily decrease MTCH2 activity, remarkably many of the mutations resulted in apparent activation of MTCH2 mediated insertion in cells. The observed activation was independent of effects on the stability or expression of MTCH2 (fig. S11C). Many of these mutations are localized to the C-terminal domain of MTCH2 which appears to have co-evolved with loss of the sixth TM, likely both critical steps during its evolution from a transporter to an insertase.

One possibility is that these mutations cause increased insertion by disrupting interactions between MTCH2 and constitutively-bound inhibitory small molecules or proteins to MTCH2. If this were the case, depletion of this putative regulatory molecule would phenocopy the activating effect. However, knockdown of known interaction partners of MTCH2, ARMC1 and DNAJC11 (38), do not activate MTCH2 function in K562 cells (fig. S12A-D). We therefore investigated whether these mutations could instead alter the intrinsic insertion propensity of MTCH2 itself, perhaps by relieving an inhibitory effect of certain amino acids towards insertion.

### Hydrophobic residues impede MTCH family insertase activity

To examine the mechanistic basis for this activation, we performed a more extensive mutational analysis aimed at identifying how hydrophobicity, size, and charge affect MTCH2 function. We selected a panel of eight positions, focusing on functionally important regions along the hydrophilic groove identified through the preliminary alanine scanning experiment. Using multiple substrates, we tested the activity of MTCH2 in human cells when one of six amino acids was present at each position (Phe, Ala, Leu, Asn, Glu, Arg) (fig. S13 A-D). For many of the sites tested, we noted a negative correlation between hydrophobicity and insertion propensity across multiple MTCH2 substrates (Fig. 3D, fig. S14A). For example, mutation of residues F228, F285, and F286 to increasingly more polar amino acids (i.e. Ala, Asn) generally resulted in a commensurate activation of MTCH2 in human cells (Fig. 3C). Remarkably this effect held across four of the eight sites tested, suggesting a shared mechanism for activation at these positions, which all line the hydrophilic vestibule. Our analysis suggests that in addition to hydrophobicity, charge and amino acid size also contribute to MTCH2 activity.

Notably though, this trend was not universal, such as position Y235, which becomes more active upon mutation to leucine. Indeed, we found a second class of mutations where there is no correlation between change in hydrophobicity and MTCH2 insertion activity (Fig. 3D). Corresponding mutations across the MTCH homologs of this second class also resulted in a similarly activating effect (Fig. 3E, fig. S15). We therefore postulated there were two distinct mechanisms for activation of MTCH2. Further, the features of the hydrophilic groove and the residues that line it seem to be conserved across the MTCH family. We think that these results are most consistent with an apparent attenuation, or tuning, of MTCH family insertase activity across holozoans, which is altered by these mutations.

### Structural analysis defines two strategies to achieve hyperactivation of MTCH2

**Fig. 4.**
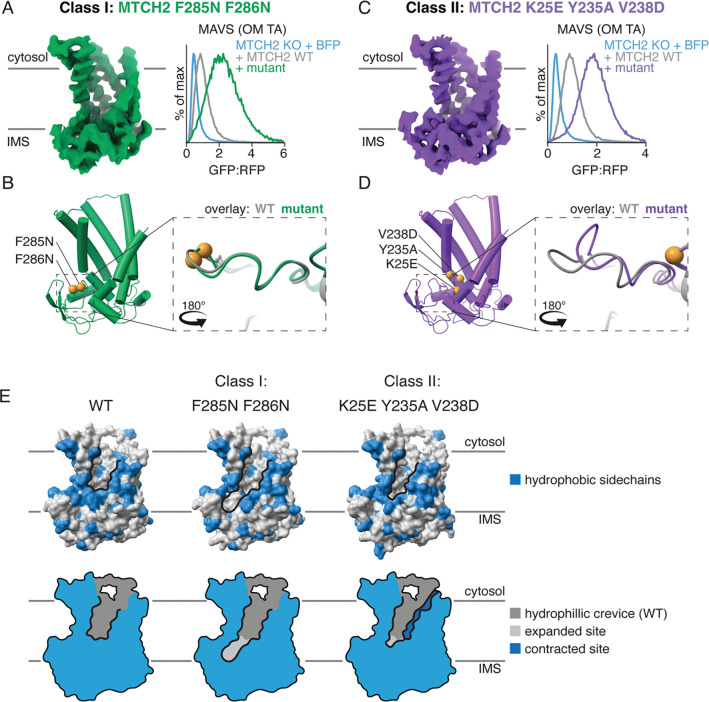
The structural basis for two distinct mechanisms of MTCH2 hyperactivation. **A)** (left) Cryo-EM density of a representative Class I MTCH2 hyperactive mutant (MTCH2^F285N, F286N^), which displays an anti-correlative relationship between hydrophobicity and insertion activity (Fig. 3D, fig. S14B). Structural determination was performed using a similar strategy for the wildtype MTCH2 as shown in Fig. 2A. (right) Activity of WT vs MTCH2^F285N, F286N^ in a MTCH2 KO K562 cell line, compared to a BFP control for MAVS insertion (additional substrates and relevant controls are displayed in fig. S14C, D). **B)** (left) The experimentally determined model of hyperactive MTCH2^F285N, F286N^ is displayed as a cartoon. The location of the two mutations, positioned along the hydrophilic groove, is highlighted (gold spheres). (right) Superposition of the WT (gray) and MTCH2^F285N, F286N^ (green) models, highlighting the C-terminal domain. **C, D)** As in (A, B) for a mutation representing the Class II MTCH2 activating mutants (MTCH2^K25E, Y235A, V238D^). At these three positions, we observed no correlation between hydrophobicity and MTCH2 activity, in contrast to the MTCH2^F285N, F286N^ mutant (Fig. 3D). MTCH2^K25E, Y235A, V238D^ induced a conformational change of residues 283–286 within the C-terminal domain of up to 7 Å, which was not observed for the MTCH2^F285N, F286N^ mutant. This movement directly repositions residues F285 and F286 (already shown to be important for MTCH2 activation in (B)) away from the hydrophilic groove (fig. S19C). **E)** (top) Displayed is the space filling representations of the experimentally determined structures of WT, MTCH2^F285N, F286N^, and MTCH2^K25E, Y235A, V238D^ with bulky hydrophobics (Phe, Leu, Met, Ile, Trp, or Tyr) shown in blue, and the hydrophilic groove outlined in black. (bottom) A simplified 2D representation highlights how these two classes of mutants result in changes to the hydrophilic groove of MTCH2 that correlate with changes in MTCH2 activity. Extensions to the hydrophilic groove when compared to WT are highlighted in light gray, while narrowing is highlighted in dark blue. The two classes of activating mutations use distinct mechanisms for achieving MTCH2 activation: (Class I) by either directly mutating hydrophobic residues that line the groove and apparently impede insertion or (Class II) by inducing a conformational change that then repositions these same hydrophobic residues.

To understand the molecular basis for this activation, we chose representative constructs for each of the two classes of activating mutations: (Class I) those where a decrease in hydrophobicity along the groove leads to increased MTCH2 activity; (Class II) those that show no correlation between MTCH2 activity and hydrophobicity. For the former category, we selected a double mutant in which two conserved aromatic hydrophobic residues within its C-terminal domain that line the groove were mutated to smaller polar amino acids. We determined the structure of this MTCH2^F285N, F286N^ to 3.2Å resolution using the same UCP1-nanobody strategy utilized for the wildtype structure (Fig. 4A, fig S16, fig. S17, Table S1). Superposition with wildtype MTCH2 identified no major conformational changes (Fig. 4B), suggesting that activation may instead be explained by how mutations to these residues directly change the size, shape, and electrostatic potential of the hydrophilic groove (Fig. 4E). We anticipate that all of the Class I mutations would behave in a similar manner to MTCH2^F285N, F286N^, where mutation of a hydrophobic residue is also predicted to increase the width of the hydrophilic groove (Fig. 4E), resulting in a corresponding increase in insertion activity.

To study the second mechanism used by the Class II mutants for MTCH2 activation, we combined three mutations identified through our systematic analysis into a triple mutant (MTCH2^K25E, V238D, Y235A^ ) that resulted in a comparable stimulation of insertion as MTCH2^F285N, F286N^ (Fig 4C, fig. S14C-E). Using the same UCP1-nanobody strategy, we determined the structure of MTCH2^K25E, V238D, Y235A^ to 3.1Å resolution (Fig. 4C, fig. S17, fig. S18, Table S1). In contrast to our Class I mutant, we observed a significant conformational change in the C-terminus of MTCH2^K25E, V238D, Y235A^ when compared to the wildtype structure (Fig. 4D, fig. S19B). This conformational change is characterized by a sharp kink in the peptide backbone around S283 and a ~7 Å shift in the Cα position for F285 and F286, repositioning these residues away from the opening of the hydrophilic crevice towards F228 and M232 on TM5 (fig. S19C, movie S1). Of note, this conformational change is not predicted in an AlphaFold3 model of MTCH2 with the same mutant (23, 24) (fig. S19D).

Taken together, both mutant structures highlight the importance of the positioning of hydrophobic amino acids lining the base of the hydrophilic crevice, which impede MTCH2’s insertase activity. While MTCH2^F285N, F286N^ directly targets two such residues, MTCH2^K25E, V238D, Y235A^ repositions these same residues away from the base of the hydrophilic groove to achieve its effect. The end result in either case is an expansion at the base of the hydrophilic groove (Fig. 4E), demonstrating the critical structural features of MTCH2’s hydrophilic groove for its activity, and revealing a concordant strategy for activation that is conserved across MTCH family insertases (Fig. 3E, fig. S15).

### Convergent evolution across OM insertases

**Fig. 5.**
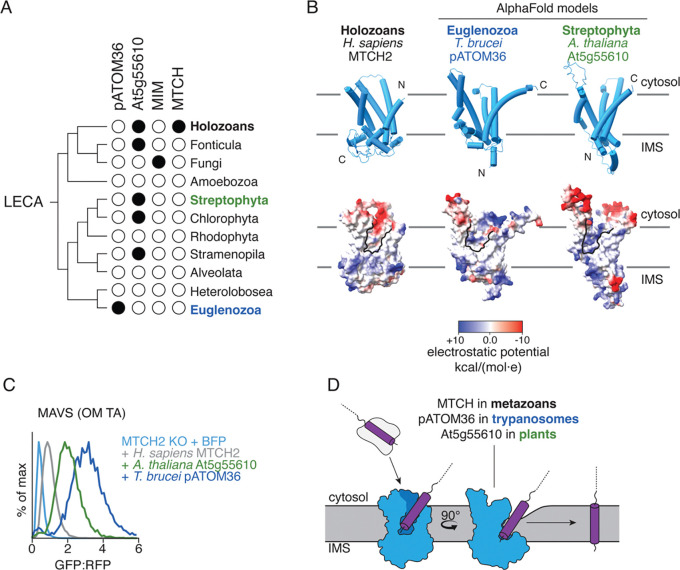
Convergent evolution of mitochondrial OM insertases across all kingdoms of life. **A)** Families of OM insertases were identified by querying Uniprot for sequences annotated with panther families PTHR10780 (MTCH), PTHR28241 (Mim1), PTHR36074 (At5g55610), or the interpro family IPR043645 (pATOM36) across eukaryotic lineages and mapped onto a species tree relative to the last eukaryotic common ancestor (LECA) (57). The presence of the putative insertase is indicated with a black circle (table S5). **B)** Comparison between the experimentally determined structure of MTCH2 (left) and AlphaFold3-predicted models of *T. brucei* pATOM36 (middle) and *A. thaliana* At5g55610 (23, 24) (right). Cartoon representations of the structures (top) are shown along with coulombic electrostatic potential mapped onto a space-filling model (bottom) with the respective hydrophilic clefts outlined in black. Topology of each insertase is based on split-GFP experiments shown in fig. S8B and S21A. Residues 1–85 and 1–60 of pATOM36 and At5g55610 respectively, which are predicted with low confidence were omitted for simplicity. **C)** To test whether At5g55610 functioned as an insertase, we tested its ability to rescue a MTCH2 KO phenotype in human cells. We expressed either a BFP control, *H. sapiens* MTCH2, *A. thaliana* At5g55610, or *T. brucei* pATOM36. Their effect on the human OM TA MAVS was measured by flow cytometry and is displayed as a histogram (additional substrates and relevant controls are displayed in fig. S21B). **D)** A universal model for insertion of α-helical proteins into the mitochondrial OM across all species. Conserved, hydrophilic grooves, along with a likely contribution of local membrane thinning, are utilized to decrease the energetic barrier for translocation of IMS-localized soluble domains across the bilayer.

While nearly all eukaryotic cells contain mitochondria, we could not identify MTCH family homologs outside of holozoa (Fig. 5A). It has previously been shown that in yeast, α-helical protein insertion into the OM relies on two single-pass proteins, Mim1 and Mim2, which are conserved across fungi (16, 17). Conversely, trypanosomes such as *T. brucei* rely on the integral OM protein pATOM36 to perform a similar function (18, 19). Plants however, have no known OM insertase.

We exploited our experimental structure of MTCH2 to perform structural homology analysis in plants using a proteomics dataset from the OM of *A. thaliana* (39). We identified a protein, At5g55610, which was predicted by AlphaFold3 to contain five TMs (23, 24), as well as a hydrophilic groove within the lipid bilayer, similar to human MTCH2, and was conserved across plant species (Fig. 5B, fig. S20). While At5g55610 is not an SLC25 carrier, its AlphaFold3-predicted model is strikingly similar to that of pATOM36. Further, it has been previously shown to localize to the OM by microscopy in *A. thaliana* (39). We found that At5g55610 and pATOM36 both localized to the OM in human cells, and experimental analysis suggested that both have an inverse topology when compared to MTCH2, with their C-terminus localized to the cytosol and N-terminus in the IMS (fig. S21A). Unlike MTCH and Mim1/2, At5g55610 and pATOM36 homologs are sporadically found throughout several distant eukaryotic lineages (Fig. 5A). One possible explanation is that this family emerged during the early evolution of plants and was acquired by other lineages through secondary endosymbiosis or horizontal gene transfer (40).

To determine if At5g55610 displayed insertase activity, we leveraged our ratiometric fluorescent reporter system in human cells to test if the protist, and putative plant insertase could rescue loss of MTCH2. Both the protist and plant OM insertases can not only express and fold in human mitochondria but can functionally replace MTCH2 for insertion of human OM proteins (Fig. 5C, fig. S21B,C), illustrating a remarkable example of convergent evolution. In fact, at similar, or lower expression levels, protist pATOM36 and At5g55610 appear to be significantly more active than human MTCH2, emphasizing the apparent attenuation of human MTCH2 activity.

## Discussion

The endosymbiotic origin of mitochondria provides an explanation for the presence of several universally conserved β-barrel proteins in the OM (41, 42). However, their increasingly complex roles in eukaryotic cells necessitated evolution of a nuclear encoded class of mitochondrial OM α-helical proteins. Unlike their β-barrel counterparts, machinery for α-helical insertion was not inherited from bacteria, suggesting that the first α-helical proteins relied on spontaneous insertion into the OM (43). The function of an OM insertase may have become necessary to better regulate an expanding OM proteome, or to enable greater plasticity in the OM composition of complex multicellular organisms. Such machinery appears to have evolved independently three times, resulting in MTCH in holozoa (8), pATOM36 in plants (18, 44), and Mim1/2 in fungus (16, 17). While these three families of insertases have no sequence similarity, all converged on similar structural features to mediate integration into the bilayer. In vertebrates, where an additional level of regulation is required to ensure adaptation and tuning in diverse cell types, there are two paralogs of the MTCH family, MTCH1 and MTCH2.

In holozoa, the MTCH family of insertases has evolved from an SLC25 carrier protein to utilize a qualitatively similar mechanism for membrane integration as the widely conserved Oxa1 Superfamily of insertases (33, 34). In particular, we have experimentally shown that MTCH activity relies on a conserved hydrophilic groove within the bilayer, produced by loss of a TM and stabilized by evolution of a conserved C-terminal domain. We propose that the MTCH hydrophilic groove primarily serves two purposes. First, it provides a hydrogen bonding surface within the membrane that can interact with the soluble domain of a substrate as it transverses the hydrophobic core of the bilayer (45). Second, numerous experimental and molecular dynamics studies have demonstrated that these grooves induce local membrane thinning (35, 46–50). Together, these effects decrease the energetic barrier for translocating a soluble domain across the membrane, catalyzing membrane insertion (Fig. 5D) (3). These hydrophilic grooves are further able to scramble lipids, which may be an additional important facet of these insertases’ function (51, 52).

However, unlike other systems, mutations to the hydrophilic groove of several MTCH family homologs resulted in hyperactivation of their insertase activity. While not common amongst insertases, attenuation of basal activity has been routinely observed for other types of membrane transporters such as ion channels (53). Structural analysis of two distinct hyperactive mutants of MTCH2 suggested that the biophysical and structural properties of its hydrophilic groove were closely correlated with its activity. Similar principles would apply to any membrane integrase that must provide a path for substrates across the bilayer. One potential explanation for the observed attenuation of MTCH activity is to tune or inhibit the intrinsic pathway for apoptosis, which plays a unique function in metazoans and is regulated by MTCH2 (8, 54). Attenuation of MTCH2’s activity may also suggest its function is regulated, either through binding of small molecules or proteins (such as ARMC1 (38)), which could act by directly blocking the hydrophilic crevice or modulating the C-terminal conformation. Such regulation could enable rapid remodeling of the OM proteome in response to metabolic or signaling cues. Given MTCH2’s central role in regulating the composition of the OM, understanding the molecular details of its activity opens the door to development of therapeutics that critically modulate MTCH2 activity as a strategy to treat human disease.

## Supplementary Material

Supplement 1

Materials and Methods

Supplementary Text

Figs. S1 to S22

Tables S1 to S5

References (58–91)

Movies S1
