# Supplementary material for: Structural evolution of the MTCH family of mitochondrial insertases
Source: bioRxiv. 2026 Feb 18:2026.02.17.705849. Preprint. [Version 1] doi: 10.64898/2026.02.17.705849 (PMC12934571; doi:10.64898/2026.02.17.705849)
Supplement: Supplementary file 1 [file media-1.pdf]

Supplementary Materials for

Structural evolution of the MTCH family of mitochondrial insertases

Taylor A. Stevens*, Zhilin Luo*, Camryn Lee*, Masami Hazu[1], Erini G. Galatis[1], Alison J. Inglis[1], Alina Guna†, Rebecca M. Voorhees†

†Correspondence to: alina.guna@ucsf.edu (A.G.); voorhees@caltech.edu (R.M.V.)

The PDF file includes:

Materials and Methods
Supplementary Text
Figs. S1 to S22
Tables S1 to S5

Other Supplementary Materials for this manuscript include the following:

Movies S1

Materials and Methods

Plasmids and antibodies

All individual plasmids are listed in Table S2-3 and are available upon request. The majority of plasmids used for expression in K562 cells are in a lentiviral vector with a UCOE EF-1α- promoter and WPRE element as previously described (*58*) (Addgene #135448). Reporter constructs used in the split GFP complementation system were fused to the eleventh strand of GFP (GFP11: RDHMVLHEYVNAAGIT) at the IMS-localized termini of the reporter (*59*, *60*). In some cases, a GS linker was added to ensure 10 amino acids of separation between the transmembrane domain (TM) and GFP11, which we had previously found to be important for sterically allowing complementation with GFP(1-10) off of the lipid bilayer. To monitor outer membrane (OM) insertion of GFP11 tagged substrates (*22*), we used a construct encoding an inner membrane space (IMS) targeting sequence containing MICU1 residues 1-60 fused with GFP(1-10) (*21*). In order to specifically query post-translational processes, such as protein insertion, the red fluorescent protein mCherry (referred to as RFP throughout the publication for brevity) was expressed along with the GFP11-fused reporters separated by a P2A ribosomal skipping sequence, yielding two polypeptides from a single open reading frame (*61–64*).

All MTCH2 rescue experiments were performed using a UCOE EF-1α- BFP-P2A-MTCH2 WPRE construct, to allow analysis of only the transduced cell population (i.e. BFP+ cells) by flow cytometry. The same strategy was used for pATOM36 family rescue experiments. Point mutations to human MTCH2 or MTCH homologs were introduced via Gibson assembly with the mutations designed into the primer overhangs. UCOE EF-1α- BFP-P2A-MTCH1 WPRE rescue constructs included an N-terminal 9x GlySer linker.

For CRISPRi knockdown experiments with a single guide, sgRNAs were ordered as a pair of complementary oligonucleotides (Integrated DNA Technologies) and ligated between the BstXi and BlpI sites in the pU6-sgRNA EF-1α-Puro-T2A-BFP vector (Addgene, #84832) (*65*). To increase knockdown efficiency, particularly for MTCH1, two sgRNAs were cloned into a programmed dual sgRNA guide vector (Addgene #140096) (*66*). To enable simultaneous CRISPRi knockdown and expression of BFP-tagged rescue constructs, the BFP was removed from the CRISPRi knockdown plasmid in some experiments, while retaining the puromycin resistance marker to allow selection of cells transduced with the sgRNA-expressing plasmid. All sgRNA sequences and backbones are listed in Table S3.

The SP64 vector was used to generate PCR templates for *in vitro* transcription reactions for subsequent translation in rabbit reticulocyte lysate (RRL) (*67*). All OM substrates included an N-terminal 3xFLAG and C-terminal 6xHis tags, the latter of which was used to immune-precipitate a proteinase K (PK) protected fragment expected upon insertion into the membrane. For OMP25, the endogenous sequence was utilized. However, because of its large size, the RHOT1 TM was instead expressed as a fusion with an N-terminal villin headpiece (VHP) domain, as previously reported (*8*). As a MTCH2 independent control, a previously established matrix-targeted control protein was generated by fusing dihydrofolate reductase (DHFR) with residues 1-69 from *N. crassa* ATP synthase subunit 9 (Su9) (*8*).

For expression in insect cells, all MTCH2 structure constructs were cloned into the pFastBac1 backbone with an N-terminal GFP-SUMO^{Eu1} tag for purification (*35*, *68*). Constructs for *E. coli* expression of α-Fab and α-UCP1 nanobodies, used for structure determination were cloned into the pET Duet vector (Millipore-Sigma) with an N-terminal pelB leader sequence for periplasmic expression and a 6x His-tag followed by a TEV protease cleavage site. *E. coli*

expression of the α-GFP nanobody (Addgene #199370) (*68*) and the $SENP^{EuB}$ protease (*69*) (Addgene #149333) were performed using a previously established plasmid system.

Reporter and rescue constructs contained several endogenous sequences obtained from UniProtKB/Swiss-Prot, including: synaptojanin-2 binding protein (OMP25/SYNJBP; **P57105-1**), import intermembrane translocase subunit Tim9 (TIM9; **Q9VYD7**), mitochondrial antiviral-signaling protein (MAVS; **Q7Z434-1**), FUN14 domain-containing protein 1 (FUNDC1; **Q8IVP5-1**), mitochondrial Rho GTPase 1 (RHOT1; **Q8IXI2-1**), mitochondrial Rho GTPase 2 (RHOT2; **Q8IXI1-1**), mitochondrial import receptor subunit TOM70 (TOMM70; **O94826**), isopentenyl-diphosphate delta-isomerase (*A. thaliana* At5g55610; **Q9FM77**), mitochondrial carrier homolog 1 (MTCH1; **Q9NZJ7-1** [MTCH1-L] and **Q9NZJ7-2** [MTCH1-S], for both long and short isoforms, respectively), mitochondrial outer membrane protein SLC25A46 (SLC25A46; **Q96AG3-1**), mitochondrial carrier protein (*C. owczarzaki* MTCH; **A0A0D2WXZ5**), mitochondrial carrier homolog 2 (*M. musculus* MTCH2; **Q791V5**), LD43650p (*D. melanogaster* MTCH; **Q9V3Y4**), mitochondrial carrier homolog 2 (*N. vectensis* MTCH; **A7SSN1** [associated sequence has been updated, exact sequence used matches XP_032228681 in NIH database]), C1q and TNF-related 4 (*X. tropicalis* MTCH2; **Q6P818**), mitochondrial carrier homolog 2 (*H. sapiens* MTCH2; **Q9Y6C9**), Uncharacterized protein (*T. brucei* pATOM36; **Q582I5** [Met 86 chosen as start codon]).

The primary antibodies used for Western Blotting are: MTCH2 (ab113707, Abcam, UK, diluted 1:1000), MTCH1 (PA5-100201, Invitrogen, USA, diluted 1:1000), SLC25A46 (12277-1-AP, Proteintech, USA, diluted 1:2000), ARMC1 (HPA026085, Atlas Antibodies, Sweden, diluted 1:1000), Tubulin (T9026, Sigma- Aldrich, USA, diluted 1:20000), FLAG-HRP (A8592, Millipore-Sigma, USA, diluted 1:10000-1:20000 depending on expression levels of FLAG-tagged construct), and DNAJC11 (17331-1-AP, Proteintech, USA, diluted 1:1000). The secondary antibodies used are: goat α-mouse-HRP (#172-1011, Bio-Rad, USA, diluted 1:5000), and goat α-rabbit-HRP (#170-6515, Bio- Rad, USA, diluted 1:5000).

Cell culture and cell lines

Human K562 containing the dCas9-KRAB machinery cells were cultured in RPMI-1640 media supplemented with 25 mM HEPES, 2.0 g/L $NaHCO_3$, 10% FBS, 2 mM glutamine, 100 U/mL penicillin, and 100 μg/mL streptomycin. Cells were maintained at a density between $0.25 \times 10^6 - 1 \times 10^6$ cells/mL. HEK293T cells were cultured in DMEM supplemented with 10% FBS, 2 mM glutamine, 100 U/mL penicillin and 100μg/mL streptomycin. Human cell lines were grown at 37 °C with 5% CO_2.

All K562 experiments were performed using a CRISPRi cell line with stable expression of $dCas9\text{-}BFP\text{-}KRAB^{KOX1}$ (*70*) and the IMS-localized MICU1(1-60)-GFP(1-10) (*8*, *21*). To generate this cell line, CRISPRi K562 cells were transduced with lentivirus expressing IMS GFP(1-10) followed by single cell sorting into a 96-well plate using a Sony Cell Sorter (SH800S). Clones expressing IMS GFP(1-10) were identified by transducing with MICU(1-60)-GFP11 and checking for GFP fluorescence. CRISPR-Cas9-derived MTCH2 KO cells were generated on top of this parental cell line, as previously described (*8*), by nucleofecting cells with two MTCH2 targeting sgRNAs (AGCCGACATGTCTCTAGTGG and GGCTTTGCGAGTCTGAACGT) which were cloned in the pX458 plasmid (Addgene #48138) with the Lonza SF Cell Line 96-well Nucleofector Kit (V4SC-2096).

ExpiSf9 cells were used to express MTCH2 for structure determination. Cells were cultured in ExpiSf CD media (ThermoFisher) and maintained between $1 \times 10^6 – 10 \times 10^6$ cells/mL at 27 °C with 0% CO_2 in an orbital shaker at 225 rpm.

Lentivirus production and spinfection

To produce lentivirus, HEK293T cells were simultaneously transfected with the two packaging plasmids (psPAX2 and pMD2.G were both gifts from Didier Trono, Addgene #12260 and #12259) and the transfer plasmid using the TransIT-293 transfection reagent (Mirus). Supernatant was collected 48 hours after transfection and aliquoted, frozen, and stored at -80° C for later use.

For generating lentivirus with any MTCH1-encoding plasmids, the pan-caspase inhibitor Q-VD-OPh (MedChemExpress) was added to a final concentration of 25 µM at the time of transfection and 24 after transfection to enable lentivirus generation by preventing apoptosis.

Bioinformatic sequence analysis

MTCH homolog sequences were identified by querying the UniProt knowledgebase (UniProtKB) for sequences annotated with the PANTHER family PTHR10780 (*71*). In order to study the distribution of MTCH homolog subfamilies across the holozoan clade, 10 holozoan species were used in species-restricted UniProtKB queries for sequences annotated with one of three PTHR10780 subfamilies: SF20 (MTCH2), SF3 (MTCH1), and SF18 (invertebrate MTCH). Query results are summarized in Table S4. To visualize the resulting information, query results were mapped onto a published holozoan species tree (Fig. 1A) (*72*).

In order to identify conserved and diverged features between MTCH and canonical SLC25 family members, we aligned individual SLC25 repeats from multiple human SLC25 family members and MTCH homologs with the hmmalign tool from the HMMER3.3 package (*73*) using a publicly available SLC25 hmm profile (pf00153) (fig. S1B) (*74*).

A putative plant OM insertase was identified from a dataset of 42 proteins confirmed to localize to the *A. thaliana* OM using isolation of mitochondria and proteomics (*39*). AlphaFold3-predicted models of each protein (*23*, *24*) were visually inspected for features similar to our MTCH2 structure, leading to the identification of At5g55610 (Fig. 5B).

In order to study the distribution of multiple OM insertase families across eukaryotes, 13 eukaryotic lineages (defined by taxid) were used in lineage-restricted UniprotKB queries for sequences annotated with the panther families PTHR10780 (MTCH), PTHR36074 (At5g55610), or PTHR28241 (Mim1) (*71*), or the pfam family PF19224 (pATOM36) (*74*). Query results are summarized in Table S5. To visualize the resulting information, query results were mapped onto a published eukaryotic species tree (Fig. 5A) (*57*). Alignments of pATOM36 homologues were produced using MUSCLE (*75*) and visualized using the ESPript 3.2 server (*76*).

Fluorescent reporter experiments in human cells

Flow cytometry data collection and analysis

For all fluorescent reporter experiments, data was collected with an Attune NXT Flow Cytometer (ThermoFisher) and analyzed in Python using the FlowCytometryTools package (v0.5.1). FSC-A vs. SSC-A gates were applied to select live cells and FSC-A vs. FSC-H gates were applied to exclude doublets. Appropriate gates selecting BFP-positive cells (for rescue or CRISPRi experiments) and RFP-positive cells (for all reporter experiments) were determined

based on measurements of non-fluorescent control cells with either the VL-1 (BFP) or YL-2 (RFP) channels. The GFP:RFP ratio was calculated from the BL-1 (GFP) and YL-2 (RFP) channels to quantify reporter activity. Histograms displaying rescue phenotypes in MTCH2 KO cells throughout the manuscript have been normalized to the median GFP:RFP ratio obtained from cells expressing WT MTCH2 ($GFP:RFP_{rescue}/GFP:RFP_{wt}$). Histograms displaying knockdown effects throughout the manuscript have been normalized to values for cells expressing a non-targeting (NT) sgRNA control ($GFP:RFP_{knockdown}/GFP:RFP_{NT}$).

CRISPRi knockdown experiments

To assess the effects of depletion of specific factors on OM protein integration, K562 CRISPRi IMS GFP(1-10) cells were spinfected with lentivirus expressing the desired sgRNA (targeting either MTCH2, ARMC1, DNAJC11 or a dual guide targeting MTCH1 as described in Table S3) and a puromycin resistance marker. Cells were then treated with puromycin (1ug/mL) at 48, 72, and 96-hour timepoints after transduction to select for sgRNA-expressing cells. Five days following the initial transduction, cells were spinfected again with lentiviral constructs expressing the RFP-P2A-GFP11-tagged reporters. For rescue experiments using MTCH1 and MTCH2 (Fig. S2A), BFP-P2A-tagged rescue constructs, and a BFP-only control, were transduced along with the reporter at an MOI of ~1. Analysis by flow cytometry took place 72 hours after the second spinfection. For all experiments, live cells expressing both BFP and RFP were analyzed for their GFP:RFP expression. We have previously established that decreases in GFP (relative to our expression control) are a proxy for successful mitochondrial integration (*8*).

Homolog rescue experiments

K562 CRISPRi IMS GFP(1-10) MTCH2 KO cells were spinfected with two individual lentiviral constructs expressing an RFP-P2A-GFP11-tagged reporter and a BFP-P2A-tagged MTCH homolog (*M. musculus*, *X. tropicalis*, *D. melanogaster, N. vectensis*, and *C. owczarzaki*), pATOM36, or At5g55610 rescue construct (*8*). Additional samples with WT and MTCH2 KO cells were transduced with a BFP-only control. Analysis by flow cytometry took place after 72 hours. The GFP:RFP ratio was analyzed for BFP- and RFP-positive cells after normalizing to the median GFP:RFP ratio obtained from cells expressing WT MTCH2 ($GFP:RFP_{homolog}/GFP:RFP_{wt}$).

MTCH2 mutational analysis experiments

K562 CRISPRi IMS GFP(1-10) MTCH2 KO cells were spinfected with two individual lentiviral constructs expressing RFP-P2A-GFP11-tagged reporters and BFP-P2A-tagged human MTCH2 WT and mutant rescue constructs. Additional samples with WT and MTCH2 KO cells were transduced with a BFP-only control. Analysis by flow cytometry took place after 72 hours. GFP:RFP was then calculated in BFP-positive and RFP-positive cells to determine whether each MTCH2 mutant could restore OM integration of each reporter construct. This was assessed by normalizing to the median GFP:RFP ratio obtained from cells expressing WT MTCH2 ($GFP:RFP_{mutant}/GFP:RFP_{wt}$). To analyze the relationship between hydrophobicity and mutational effects, a hydrophobicity scale (*56*) was used to calculate the hydrophobicity shift in the amino acid at each position (Δhydrophobicity) caused by each mutation. Specific values used were 100 for Phe, 97 for Leu, 76 for Val, 74 for Met, 63 for Tyr, 41 for Ala, -5 for Ser, -10 for Ala, -14 for Arg, -23 for Lys, -28 for Asn, and -31 for E. Δhydrophobicity was calculated by the following formula: $hydrophobicity_{aa\ mut}$-$hydrophobicity_{aa\ wt}$, where $hydrophobicity_{aa\ mut}$ is the

hydrophobicity of the mutated amino acid and $hydrophobicity_{aa\ wt}$ is the hydrophobicity of the WT amino acid at this position. To assess correlations, the split-GFP reporter mutant phenotypes were plotted against Δhydrophobicity, a linear equation was fit to the resulting data points in python using numpy (*77*), and the coefficient of determination (R^2) was calculated with the following formula: $1 – SS_{res}/SS_{tot}$ where SS_{res} is the residual sum-of-squares (the squared difference of each GFP:RFP value from the linear fit) and SS_{tot} is the total sum-of-square (the squared difference of each GFP:RFP value from the mean of all GFP:RFP values) (Fig. 3D and S14A,B).

OMM topology experiments

K562 CRISPRi IMS GFP(1-10) cells were spinfected with lentiviral constructs expressing RFP-P2A-GFP11 tagged proteins. Analysis by flow cytometry took place after 72 hours. GFP:RFP was then calculated in RFP-positive cells to determine if the GFP11 tagged termini of each protein was localized in the IMS (Fig. S8B-C, S21A).

<u>*In vitro* mitochondrial insertion</u>

Mitochondrial Isolation

To directly test if *C. owczarzaki* MTCH was active at the level of insertion, we generated human K562 cells depleted of MTCH2 but expressing either the human or *C. owczarzaki* MTCH. More specifically, we generated CRISPRi IMS GFP(1-10) MTCH2 KO cells expressing BFP-P2A tagged human 3xFLAG-MTCH2 or *C. owczarzaki* 3xFLAG-MTCH rescue constructs by lentiviral transduction at an MOI of ~1. WT cells and MTCH2 KO cells expressing a BFP-only control were also generated. ~20 million BFP expressing cells for each of the four samples were isolated using a BD Biosciences FACS Aria Fusion Cell Sorter, and expanded for 5-7 days prior to harvesting for use in mitochondrial isolation experiments.

Mitochondria were isolated similarly to a previously described protocol (*8*, *78*, *79*). K562 cells were centrifuged for 5 minutes at 220x g. The resulting pellets were then washed in homogenization buffer (210 mM mannitol, 70 mM sucrose, 5 mM HEPES pH 7.4, 10 mM EDTA, 1 mM PMSF, 2 mg/mL BSA), resuspended in 2 mL of the same buffer, and incubated on ice for 10 minutes. A 2 mL glass Dounce homogenizer with a tight-fitting pestle was used to lyse cells using ~30 passes. After lysis, the cell lysate was centrifuged at 1300x g for 5 minutes. The supernatant was transferred to a clean tube and centrifuged twice more to remove all nuclei and unlysed cells. The supernatant was then transferred to a new tube and centrifuged at 11,000x g for 10 minutes to pellet mitochondria. Mitochondria were washed 2x in isolation buffer (210 mM mannitol, 70 mM sucrose, 5 mM HEPES pH 7.4, 10 mM EDTA) and the final pellet was resuspended in 5-10µl isolation buffer. Prior to downstream use, protein concentration was measured using a Bradford assay and each mitochondrial sample was normalized by diluting to a protein concentration of 5 mg/mL.

In vitro *translation and insertion*

In vitro translation was performed using rabbit reticulocyte lysate (RRL) (*67*) and mitochondrial insertion was assessed as previously described (*8*, *78*). Transcription templates were amplified via PCR using primers which bound upstream of the SP6 promoter and ≥ 12 bp downstream of the stop codon, and purified using the QIAquick PCR purification kit (Qiagen). The DNA template was then transcribed using SP6 polymerase at 37 °C for 90 minutes. mRNA

was then translated for 15-30 minutes at 32 °C in the presence of radioactive ^{35}S-methionine, followed by addition of 1 mM puromycin to end translation and ensure nascent chain release from the ribosome.

Insertion reactions with isolated mitochondria were performed by diluting 4µl translation into 50µl import buffer (250 mM sucrose, 5 mM $Mg(Acetate)_2$, 80 mM KAcetate, 20 mM HEPES pH 7.4, 2.5 mM ATP, and 15 mM succinate) and adding 15 µg purified mitochondria, followed by incubation at 32 °C for 30 minutes.

To determine insertion efficiency, proteinase K was added at a concentration of 0.25 mg/mL followed by incubation on ice for 1 hour. Reactions were quenched by addition of 5 mM PMSF in DMSO, followed by transfer to boiling 1% SDS in 0.1 mM Tris/HCl pH 8.0. 6xHis-tagged protected fragments were affinity purified by diluting PK-digested samples in IP buffer (50 mM HEPES pH 7.5, 500 mM NaCl, 10 mM imidazole, 1% Triton X-100) and incubating with 10 µL NiNTA resin while mixing for 1.5 hours at 4 °C. The resin was then washed 3x with 1 mL IP buffer, and the enriched fragments were then eluted from resin in Laemmli Sample buffer supplemented with 50 mM EDTA pH 8.0. PK protected fragments were quantified after running samples on SDS-PAGE gels and imaging with autoradioagraphy.

Structural determination of human MTCH2

Design of human MTCH2 expression constructs for structure

Due to its small size (33 kDa), structure determination of MTCH2 required fiducial markers to enable the accurate determination of particle orientation and to allow 3D reconstruction. We utilized two strategies to do this. First, we tested fusion to the small globular domain, BRIL, which itself can be recognized by a semi-synthetic Fab (BAK5) (*28*) which was further stabilized by an additional α-Fab nanobody (Nb) (*31*), resulting in a trimeric complex of ~100 kDa. While partially successful, we observed significant flexibility between the BRIL fusion and MTCH2 in these reconstructions that limited the resulting resolution.

In addition, we reasoned we could leverage the tools developed for determination of the recent structure of the SLC25, UCP1 (*29*, *55*). In this case, an α-UCP1 Nb (pMb65) was developed that specifically recognized the loop between TMs 4 and 5 of UCP1, which is a similar length in all SLC25s and MTCH family homologs. We therefore created a MTCH2 fusion with the short sequence of UCP1 that is recognized by this α-UCP1 Nb. The α-UCP1 Nb was engineered to enable the binding of a universal α-nanobody Fab which was further stabilized through the binding of an additional α-Fab Nb, resulting in a tetrameric complex of ~110 kDa.

BRIL fusion construct | To design the MTCH2-BRIL fusion MTCH2(BRIL), human MTCH2 residues 207-214 were replaced with an engineered variant of apocytochrome b562 (BRIL) flanked by two α-helical linkers, in order to enable complexing with synthetic antibodies to facilitate cryo-EM structure determination (*28*). Rosetta (*80*, *81*) was used to identify specific linker lengths predicted to form rigid α-helices, and optimal sequences were designed from the resulting backbone using ProteinMPNN (*82*). The designs were then further optimized by iterative modification based on AlphaFold3 predictions (*23*, *24*).

UCP1 fusion construct | To design the MTCH2-UCP1 fusion, MTCH2(UCP1), human MTCH2 residues 203-222 were replaced with UCP1 residues 198-218, and an additional 3 stabilizing mutations were included: L199V, A200Y, T226A to recreate an α-UCP1 Nb (pMb65) binding epitope (*29*).

To enable the formation of a larger complex containing MTCH2 (UCP1), the TC-Nb4 interface was engineered onto pMb65 to allow the binding of the nanobody-binding-Fab (NabFab) (*30*) by removing the first three residues (GPS) and introducing 6 point mutations: V5R, A17P, P27A, K89E, Q123K, and Q126P.

MTCH2 UCP1 mutant constructs | To determine structures of representative members of the Class I and Class II hyperactive MTCH2 mutants, we introduced mutations into the MTCH2 UCP1 fusion construct. For the Class I F285N F286N mutant, we used blunt end ligation with phosphorylated primers in which the mutations were designed into the 5' end of one primer. For the Class II K25E Y235A V23D triple mutant, we used Gibson assembly in which the mutations were designed into the primer overhangs.

Expression construct design | The resulting MTCH2 sequences were cloned into a baculovirus expression plasmid pFastBac1 to enable expression in Sf9 cells. The MTCH2 sequence was fused to a GFP-SUMO^{Eu1} tag to enable native purification using the GFP Nb strategy previously described by our lab in which GFP Nb is immobilized to affinity resin (in this case NiNTA), and MTCH2 is eluted by cleavage of the SUMO^{Eu1} by incubation the engineered protease SENP^{EuB}.

Expression and purification of SENP^{EuB} protease

To allow the native elution of untagged MTCH2 following isolation using the GFP Nb, we used SENP^{EuB} protease, because we had previously shown this results in better yield and cleaner preparations than other strategies. Expression and purification of the SENP^{EuB} protease (*68*) was conducted based on a previously described protocol, modified with an additional size exclusion step to reduce contaminants interfering with structure determination efforts. In brief, the plasmid was transformed into NEBExpress *E. coli* expression strain (New England Biolabs). A single colony was inoculated into 50 mL Luria-Bertani (LB) medium supplemented with 50 µg/mL kanamycin and incubated shaking at 37 °C for 4-5 hours. 5 mL of overnight culture was inoculated into 1 L SuperBroth (SB) medium supplemented with 50 µg/mL kanamycin and incubated at 37 °C while shaking at 220 rpm. Expression was induced by addition of 1mM IPTG when OD_{600} = 0.8. Cells were grown for 6 hours at 18 °C post induction and harvested by centrifugation. Cells were resuspending in 50 mL resuspension buffer (50 mM Tris/HCl pH 7.5, 500 mM NaCl, 10 mM imidazole, and 5 mM beta-mercaptoethanol) and flash frozen for later use.

The resulting resuspension was thawed and supplemented with cOmplete™ EDTA-free Protease Inhibitor Cocktail (Roche), lysed by sonication, and cleared by centrifugation at 18,000 rpm and 4 °C for 30 minutes using an SS-34 rotor (Beckman-Coulter). Cleared lysate was incubated with 1 mL Ni-NTA agarose (Qiagen) for 15 minutes while rolling at 4 °C. Ni-NTA resin was then loaded onto a gravity flow column (Bio-Rad) and washed with 100 mL resuspension buffer. Protein was eluted with 5 mL of elution buffer (50 mM Tris pH 7.5, 150 mM NaCl, 500 mM Imidazole, and 5 mM beta-mercaptoethanol). Eluted protein was then buffer exchanged into storage buffer (50 mM Tris pH 7.5, 150 mM NaCl, 10 mM Imidazole, 10% [v/v] glycerol, and 5 mM beta-mercaptoethanol) using PD-10 desalting column (Cytiva). Protein was then cleaved overnight with TEV protease. After cleavage, protein was flowed over NiNTA resin to remove any remaining un-cleaved protein. The cleaved flowthrough was then concentrated using a 10K MWCO concentrator (Millipore-Sigma) and injected onto a S200 increase 10/300 GL size exclusion column (Cytiva) equilibrated in SEC buffer (150 mM NaCl, 50 mM Tris pH

7.5, 10% glycerol [v/v], and 1 mM DTT). The protein-containing peak was pooled, concentrated, aliquoted, and then flash frozen in liquid nitrogen and stored for later use.

Expression and purification of α-GFP Nb

We leveraged the extremely specific and high affinity interaction between the GFP Nb and GFP-MTCH2 to allow isolation in a single step from Sf9 cells. Expression and purification of the α-GFP Nb was conducted as previously described (*68*). Briefly, the plasmid was transformed into AVB101 *E. coli* expression strain (New England Biolabs). A single colony was inoculated into 100 mL LB medium supplemented with 50 µg/mL kanamycin plus 10 µg/mL carbenicillin and incubated at 37 °C while shaking at 220 rpm overnight. 5 mL overnight culture was inoculated into 1 L SB medium supplemented with 50 µg/mL kanamycin, 10 µg/mL carbenicillin, and 50 µM biotin, and grown to an OD_{600} = 1 up while shaking at 37 °C. Expression was induced by addition of 1 mM IPTG. Cells were grown overnight at 18 °C post induction and harvested by centrifugation. Cells were resuspending in 50 mL wash buffer (50 mM Tris/HCl pH 7.5, 500 mM NaCl, and 10 mM imidazole) and flash frozen for later use.

After thawing, the resuspension was supplemented with cOmplete™ EDTA-free Protease Inhibitor Cocktail (Roche), subsequently lysed by sonication, and cleared by centrifugation at 18,000 rpm and 4 °C for 30 minutes using an SS-34 rotor (Beckman-Coulter). Cleared lysate was incubated with 1 mL Ni-NTA agarose (Qiagen) for 15 minutes while rolling at 4 °C. Ni-NTA resin was then loaded onto a gravity flow column (Bio-Rad) and washed with 100 mL or wash buffer. Protein was eluted with 5 mL of elution buffer (50 mM Tris pH 7.5, 150 mM NaCl, and 500 mM Imidazole). Eluted protein was then buffer exchanged into storage buffer (50 mM Tris pH 7.5, 150 mM NaCl, 10 mM Imidazole, and 10% [v/v] glycerol) using a PD-10 desalting column (Cytiva), aliquoted, and flash frozen for later use.

Expression and purification of α-UCP1 and α-Fab Nbs

To increase the size of MTCH2 for structure determination, we used purified α-UCP1 and α-Fab Nbs to form the MTCH2(BRIL)•BAK5•α-Fab Nb and MTCH2 (UCP1)•α-UCP1 Nb•NabFab•α-Fab Nb complexes with the modified MTCH2 sequences described above. The α-UCP1 Nb is based on a previously characterized nanobody (*29*) further engineered to allow binding to NabFab (*30*), while the α-Fab Nb (*31*) aids in structure determination by binding to the hinge region of the NabFab and BAK5 synthetic antibodies in order to increase overall complex size, break symmetry of the Fab, and reduce flexibility. To express the α-UCP1 and α-Fab Nbs, plasmids were transformed into BL21 (DE3) *E. coli* expression strain. A single colony was inoculated into 50 mL Luria-Bertani medium supplemented with 100 µg/mL carbenicillin and incubated at 37 °C while shaking at 220 rpm overnight. 5 mL of overnight culture was inoculated into 1 L SB medium supplemented with 100 µg/mL carbenicillin. Expression was induced by addition of 1mM IPTG when OD_{600} = 0.8. Cells were grown for 4 hours after induction and harvested by centrifugation. Pelleted cells were resuspended in 50 mL wash buffer (50 mM Tris pH 7.5, 200 mM NaCl, 10 mM Imidazole) and flash frozen for later use.

After thawing, the resuspension was supplemented with cOmplete™ EDTA-free Protease Inhibitor Cocktail (Roche), subsequently lysed by sonication, and cleared by centrifugation at 18,000 rpm and 4 °C for 30 minutes using an SS-34 rotor (Beckman-Coulter). Cleared lysate was incubated with 1 mL Ni-NTA agarose (Qiagen) for 15 minutes while rolling at 4 °C. Ni-NTA resin was then loaded onto a gravity flow column (Bio-Rad) and washed with 100 mL of wash buffer. Protein was eluted with 5 mL of elution buffer (50 mM Tris pH 7.5, 150 mM NaCl, and

500 mM Imidazole). Eluted protein was then buffer exchanged into storage buffer (50 mM Tris pH 7.5, 150 mM NaCl, 10 mM Imidazole, and 10% [v/v] glycerol) using PD-10 desalting column (Cytiva). Protein was then cleaved overnight with TEV protease. After cleavage, protein was flowed over NiNTA resin to remove any remaining uncleaved protein. The cleaved flowthrough was then concentrated using a 10K MWCO concentrator (Millipore-Sigma) and injected onto a S200 increase 10/300 GL size exclusion column (Cytiva) equilibrated in SEC buffer (150 mM NaCl, 50 mM Tris pH 7.5, 10% glycerol [v/v]). The protein-containing peak was pooled, concentrated, aliquoted, and then flash frozen in liquid nitrogen and stored at -80 °C for later use.

Expression and purification of synthetic antibodies NabFab and BAK5

To further increase the size of MTCH2 for structure determination, we used the synthetic antibodies BAK5 and NabFab to form the MTCH2(BRIL)•BAK5•α-Fab Nb and MTCH2 (UCP1)•α-UCP1 Nb•NabFab•α-Fab Nb complexes with the modified MTCH2 sequences described above. BAK5 binds to a BRIL domain that we engineered into the loop in between MTCH2 TM4 and TM5, while NabFab binds to an interface that was engineered into the α-UCP1 Nb sequence. The pRH2.2 NabFab plasmid was transformed into C43(Pro+) *E. coli* expression strain (both plasmid and expression strain provided as a kind gift by A. Kossiakoff) (*30*). The pRH2.2 α-BRIL Fab BAK5 was transformed into BL21-Gold *E. coli* expression strain (Agilent). All remaining steps were the same for both constructs. A single colony was used to inoculate a culture in Luria Broth (LB) medium supplemented with 100 µg/mL carbenicillin for overnight incubation while shaking at 37 °C 220 rpm. 20mL of overnight culture was subsequently inoculated into 2 L SB medium supplemented with 100 µg/mL Carbenicillin. Expression was induced by addition of 1mM IPTG when OD_{600} = 0.8. Cells were grown for 4 hours at 37 °C after induction and harvested by centrifugation. Pelleted cells were flash frozen for storage at -80 °C.

The resulting pellet was resuspended in lysis buffer (50 mM Tris pH 7.5, 200 mM NaCl, and 1x cOmplete™ EDTA-free Protease Inhibitor Cocktail [Roche]). Cells were lysed by sonication. The lysate was incubated at 62 °C for 30 minutes and cleared by centrifugation at 18,000 rpm and 4 °C for 30 minutes using a SS-34 rotor (Beckman-Coulter). Cleared lysate was subsequently loaded onto a 5 mL Pierce™ Protein L column (ThermoScientific) and washed with 50 mL of wash buffer (50 mM Tris pH 7.5 and 500 mM NaCl). Protein was eluted with 100 mM acetic acid and immediately neutralized by 1M Tris. Protein-containing fractions were loaded onto a 1 mL HiTrap SP cation-exchange column (Cytiva) and washed with 5 mL of cation exchange wash buffer (50 mM Na Acetate pH 5.0). The protein was eluted by running a 0-100% gradient over 25 mL of cation exchange elution buffer (50mM Na Acetate pH 5.0 and 2M NaCl) followed by immediate neutralization using 1 M Tris. Purified protein was buffer exchanged into storage buffer (50 mM Tris pH 7.5, 200mM NaCl, and 5% glycerol) using a PD-10 desalting column (Cytiva). Buffer exchanged protein was concentrated using a 30K MWCO concentrator (Millipore-Sigma), then flash frozen in liquid nitrogen and stored at -80 °C for later use.

Baculovirus-based expression of MTCH2 in insect cells for structure determination

To obtain large quantities of the complex-forming MTCH2 variants described above (BRIL and UCP1), sequences were fused to an N-terminal GFP-SUMO^{Eu1} tag (GFP-SUMO^{Eu1}-MTCH2) and cloned into the pFastbac1 backbone to enable expression in insect cells with baculovirus. The plasmids were transformed into MAX Efficiency™ DH10Bac competent cells

(Gibco) and the PureLink^{TM} HiPure plasmid miniprep kit (Invitrogen) was used to obtain bacmid DNA. Bacmid DNA was then transfected in ExpiSf9 cells using the ExpiFectamine™ Sf transfection reagent (Gibco) and cell viability was monitored over several days. After cell viability dropped below 80%, cells were pelleted by centrifugation at 300x rcf for 5 minutes and the baculovirus containing supernatant (P0) was stored at 4 °C protected from light for later use. ExpiSF cells were mixed with a dilution series of baculovirus and cultured for 24 hours, and the then the % GFP-positive cells was measured using the Attune NxT flow cytometer (ThermoFisher) with a BL1 channel gate generated using uninfected ExpiSF cells. Next, a larger culture of ExpiSF cells was mixed with P0 baculovirus at a multiplicity of infection (MOI) of 0.075 and cell viability was monitored for several days. After cell viability dropped below 80%, cells were pelleted by centrifugation at 300x g for 5 minutes and the baculovirus containing supernatant (P1) was stored at 4 °C protected from light for later use.

For protein expression, ExpiSf9 cells were seeded at a density of 2.5 x 10^6 cells/mL and ExpiSf Enhancer (Gibco) was added. On the next day, P1 baculovirus was added at a MOI of 2. The cells were harvested after 48 hours by centrifugation at 6,000 rpm and 4 °C for 10 minutes in a H-6000A rotor (Sorvall). The resulting pellet was washed by resuspending in phosphate-buffered saline and centrifuging again at 3500x rcf and 4 °C for 10 minutes. The supernatant was then discarded and the pellet was weighed, then flash frozen in liquid nitrogen and stored at -80 °C for later use.

Purification of MTCH2 (UCP1) and (BRIL) for structure determination

GFP-SUMO^{Eu1}-MTCH2 (UCP1 or BRIL) was purified using an α-GFP Nb as previously described (*68*) with some modifications. To isolate a mitochondrially-enriched membrane fraction, cells were resuspended in hypotonic lysis buffer (10mM HEPES/KOH pH 7.5, 10 mM K Acetate, 1.5 mM Mg Acetate, 2 mg/mL Iodoacetamide, and 1x cOmplete^{TM} EDTA-free Protease Inhibitor Cocktail [Roche]) and incubated on ice for 10 minutes followed by lysis using a 40 mL glass Dounce homogenizer with a loose-fitting pestle, followed by addition of NaCl to 190 mM. Membranes were pelleted by centrifugation for 10 minutes at 18,000 rpm and 4 °C using an SS-34 rotor (Beckman-Coulter). The pelleted membranes were washed 1x with membrane wash buffer (10 mM HEPES/KOH pH 7.5, 200 mM NaCl, 1.5 mM Mg Acetate) and resuspended at a total protein concentration of 2.5 mg/mL in solubilization buffer (50 mM HEPES/KOH pH7.5, 2 mM Mg Acetate, 50 mM imidazole, 2 mg/mL Iodoacetamide, 1x cOmplete^{TM} EDTA-free Protease Inhibitor Cocktail [Roche], and 2% [w/v] n-Undecyl-β-D-Maltoside [UDM; Anatrace]) and incubated for 1 hour at 4 °C while rolling. Debris was removed by centrifugation for 30 minutes at 18,000 rpm and 4 °C using an SS-34 rotor.

Molar concentration of GFP-SUMO^{Eu1}-MTCH2 in the solubilized membrane fraction was estimated by measuring fluorescence with a purified GFP standard in a BioTek Synergy HTX plate reader (Agilent). 14x His-tagged GFP Nb was then added to the solubilized membrane fraction at a 2-fold molar excess to GFP-SUMO^{Eu1}-MTCH2 and incubated with Ni-NTA agarose resin (QIAGEN, 30210) at 4 °C for 1 hour while rolling. Ni-NTA resin was then washed two times in wash buffer A (50 mM HEPES/KOH pH 7.5, 2 mM Mg Acetate, 50 mM imidazole, 1x cOmplete^{TM} EDTA-free Protease Inhibitor Cocktail [Roche], 0.5 mM ATP, and 0.1% UDM), with a 15 minute incubation per wash step. Lastly, Ni-NTA resin was washed twice with wash buffer B (50 mM HEPES/KOH pH 7.5, 2 mM Mg Acetate, 50 mM imidazole, 1x cOmplete^{TM} EDTA-free Protease Inhibitor Cocktail [Roche], and 0.07% UDM). Protein was eluted by incubating with wash buffer B + 600 nM SENP^{EuB} protease for 1 hour at 4 °C while

rolling. Eluted protein was concentrated to 500uL using 30K MWCO concentrator (Millipore-Sigma) and injected onto a Superdex 200 Increase 10/300 GL column (Cytiva) equilibrated in size exclusion buffer (50 mM HEPES/KOH pH 7.5, 2 mM Mg Acetate, and 0.07% UDM). MTCH2-containing fractions were then pooled prior to downstream complexing steps.

For MTCH2^{F285N,F286N} and MTCH2^{K25E,Y235A,V238D}, the same purification strategy was used with the following differences: after washing twice with buffer A, an additional wash step was performed with wash buffer A2 (50 mM HEPES/KOH pH 7.5, 10 mM K Acetate, 2 mM Mg Acetate, 50 mM imidazole, 1x cOmpleteTM EDTA-free Protease Inhibitor Cocktail [Roche], and 1% Triton X-100 [v/v]).

MTCH2 complex assembly for structure determination

MTCH2 (BRIL)•BAK5•α-Fab Nb complex formation | Size-exclusion purified MTCH2 (BRIL) was mixed with BAK5 and α-Fab Nb at a 1:1.5:2 molar ratio followed by incubation at 4 °C overnight. The mixture was then concentrated to 100 μL and injected onto a Superdex 200 Increase 10/300 GL column (Cytiva) equilibrated in size exclusion buffer (fig. S4B). Fractions containing the MTCH2 (BRIL)•BAK5•α-Fab Nb complex were pooled and concentrated using a 100K MWCO concentrator (Millipore-Sigma).

MTCH2 (UCP1)•α-UCP1 Nb•NabFab•α-Fab Nb complex formation | Size-exclusion purified MTCH2 (UCP1) was mixed with α-UCP1 Nb, NabFab, and α-Fab Nb at a 1:1.5:2:2.5 molar ratio and incubated overnight at 4 °C. The mixture was concentrated to 500 μL and injected onto a Superdex 200 Increase 10/300 GL column (Cytiva) equilibrated in size exclusion buffer (fig. S6B). Fractions containing the MTCH2 (UCP1)•α-UCP1 Nb•NabFab•α-Fab Nb complex were pooled and concentrated using a 100K MWCO concentrator (Millipore-Sigma).

Cryo-EM grid preparation and data collection

To prepare cryo-EM grids, 3.1 μL of either MTCH2 (BRIL)•BAK5•α-Fab Nb complex or MTCH2 (UCP1)•α-UCP1 Nb•NabFab•α-Fab Nb complex was applied to Quantifoil R 1.2/1.3 holey carbon film grids (Ted Pella, Inc.) which had been glow-discharged with a PELCO easiGlow™ (Ted Pella, Inc.) at 20 mA for 60 s. A FEI Vitrobot Mark IV (Thermo Fisher) was used to blot grids with filter paper and then plunge freeze them in liquid ethane. Blotting was carried out at 6 °C and 100% humidity with -1 blot force for 5s. The protein concentration used for grid preparation was 3.5 mg/mL for the MTCH2 (BRIL)•BAK5•α-Fab Nb complex, 5.9 mg/mL for the WT MTCH2 (UCP1)•α-UCP1 Nb•NabFab•α-Fab Nb complex, 3.4 mg/mL for the MTCH2^{F285N,F286N} (UCP1)•α-UCP1 Nb•NabFab•α-Fab Nb complex, and 4.9 mg/mL for the MTCH2^{K25E,Y235A,V238D} (UCP1)•α-UCP1 Nb•NabFab•α-Fab Nb complex.

Data were collected on a Titan Krios electron microscope (Thermo Fisher) at 300 keV equipped with a K3 direct electron detector (Gatan) and a 20 eV slit width energy filter. Automatic image acquisition was performed using SerialEM in super resolution mode at a magnification of 105,000, corresponding to a calibrated pixel size of 0.418 Å/pixel. Images were collected at a defocus range of -0.8 to -1.8 μm and a total dose of 60 $e^-/Å^2$. We collected for a total of 26,889 micrographs for the MTCH2 (BRIL)•BAK5•α-Fab Nb complex, 15,334 micrographs for the WT MTCH2 (UCP1)•α-UCP1 Nb•NabFab•α-Fab Nb complex, 10,506 micrographs for the MTCH2^{F285N,F286N} (UCP1)•α-UCP1 Nb•NabFab•α-Fab Nb complex, and 10,816 micrographs for the MTCH2^{K25E,Y235A,V238D} (UCP1)•α-UCP1 Nb•NabFab•α-Fab Nb complex.

Cryo-EM image processing

Graphical schematics of the processing workflow for each structure are shown in fig. S4, S6, S16, and S18. All data processing was performed with CryoSPARC v4.7.0 (*83–85*). Movies were downsampled to 0.836 Å/pixel, motion-corrected, dose-weighted, and the contract transfer function (CTF) was estimated in CryoSPARC Live.

For MTCH2 (BRIL)•BAK5•α-Fab Nb, 23,738 out of 26,889 micrographs were selected for downstream processing based on CTF quality, ice thickness, and visual inspection. The blob picker was used on 6,937 micrographs to pick 4,622,038 particles which were extracted at 4.16 Å/pixel. After iterative rounds of 2D classification, classes containing 50,000 particles were selected for ab-initio reconstruction to generate 3 volumes. One of the 3 volumes resembled a Fab attached to a detergent solubilized membrane protein and was used to generate templates for template picker. Resulting particle picks were combined with the initial particle set for a total of 7,786,491 particles. Together, 11 rounds of heterogenous refinement were conducted using one ab-initio volume corresponding to the MTCH2 complex and four decoy volumes, resulting in a final particle set containing 199,397 particles, which were then extracted at 0.998 Å/pixel. A final map was obtained from non-uniform refinement. Local resolution was calculated using a mask from the non-uniform refinement job and visualized in ChimeraX. Settings of the data collection are reported on Table S1.

For MTCH2 (UCP1)•α-UCP1 Nb•NabFab•α-Fab Nb, 14,190 of out 15,334 micrographs were selected for downstream processing based on CTF quality, ice thickness, and visual inspection. The blob picker was used on 4,885 micrographs to pick 1,082,415 particles which were extracted at 3.44 Å/pixel. After iterative rounds of 2D classification, classes containing 160,865 particles were selected for to ab-initio reconstruction to generate 3 volumes. One of the 3 volumes resembled a Fab attached to a detergent solubilized membrane protein and was used to generate templates for template picker. Resulting particle picks were combined with the initial particle set for a total of 4,791,546 particles. Together, 11 rounds of heterogenous refinement were conducted using one ab-initio volume corresponding to the MTCH2 complex and four decoy volumes, resulting in a final particle set containing 485,622 particles, which were then extracted at 0.83 Å/pixel. The extracted particles were processed to correct for anisotropic magnification using the global CTF refinement job and the motion-correction and dose-weighting was refined using reference based motion correction. After this processing, a final map was obtained from non-uniform refinement followed by local refinement with mask on MTCH2 only. Local resolution was calculated using a mask for MTCH2 and visualized in ChimeraX. B-factor was calculated and visualized in ChimeraX. Settings of the data collection are reported on Table S1.

For MTCH2^{F285N,F286N} (UCP1)•α-UCP1 Nb•NabFab•α-Fab Nb, the same processing steps were performed with the following differences: 6,937 out of 10,506 micrographs were selected for downstream processing. A total of 2,013,310 particles from blob picker and template picker were obtained. After running 5 rounds of heterogenous refinement, 321,419 particles were selected for further processing.

For MTCH2^{K25E,Y235A,V238D} (UCP1)•α-UCP1 Nb•NabFab•α-Fab Nb, the same processing steps were performed with the following differences: 9,633 out of 10,816 micrographs were selected for downstream processing. A total of 3,547,162 particles from blob picker and template picker were obtained. After running 7 rounds of heterogenous refinement, 612,877 particles were selected for further processing.

Model building and refinement

For MTCH2 (BRIL)•BAK5•α-Fab Nb, an AlphaFold3 predicted model of MTCH2 (BRIL) (*23*, *24*) was docked into the EM density using ChimeraX (*86*). There was prominent density for the four-helix bundle of BRIL with one rigid helix linking the C-terminus of BRIL with TM5 of MTCH2. Low resolution density could be observed for the remainder of MTCH2, roughly confirming the positions of TM3 and TM4, but the rest of the density was too poor to be useful for modelling.

To build MTCH2 (UCP1)•α-UCP1 Nb, the initial model for MTCH2 (UCP1) was generated with AlphaFold3 (*23*, *24*) using the sequence corresponding to the MTCH2 (UCP1), and the initial model for the α-UCP1 Nb was derived from a previously determined structure (PDB ID 8G8W) (*29*). The initial models were loaded into ChimeraX (*86*) and docked into the EM density, then the docked models were loaded into COOT (*87*) and the TC-Nb4 scaffold residues which enable NabFab binding were manually mutated. The models were manually rebuilt using COOT, including adjustment to the C-terminal region (285-301) which was poorly predicted by AlphaFold. The model was then subsequently subjected to iterative refinement with *phenix.real_space_refinement* (*88*) using secondary restraints and Ramachandran restrains. Statistics of the EM map and model refinement are reported in Table S1.

The complete model for the WT $MTCH2^{UCP1}$•α-UCP1 Nb complex was docked into the maps for $MTCH2^{F285N,F286N}$ (UCP1)•α-UCP1 Nb•NabFab•α-Fab Nb and $MTCH2^{K25E,Y235A,V238D}$ (UCP1)•α-UCP1 Nb•NabFab•α-Fab Nb and subject to further manual building in COOT, including a significant conformational change of residues 283-286 in the $MTCH2^{K25E,Y235A,V238D}$ model, and further rounds of iterative refinement with *phenix.real_space_refinement*. Since no density was observed for the loop in between TM2 and TM3 (residues 100-115) this region was excluded from the final model.

Structural model analysis

Molecular analysis and graphic were performed utilizing USCF ChimeraX (*86*) and PyMOL (*89*). Coulombic electrostatic potential was calculated and mapped onto the surface representation of each structure in ChimeraX. Previously determined experimental structures of Ylp1, YidC, the endoplasmic reticulum (ER) membrane protein complex (EMC), uncoupling protein 1 (UCP1), and the ATP/ADP translocase were used in comparisons with our MTCH2 structure (*25*, *26*), while models of MTCH homologs, *T. brucei* pATOM36, and *A. thaliana* At5g55610 were generated using AlphaFold3 (*23*, *24*). In surface representations of MTCH2 and other models, a black line was drawn to outline the hydrophilic crevice, which we define as a concave hydrophilic surfaces flanked by hydrophobic TM residues.

Fig S1

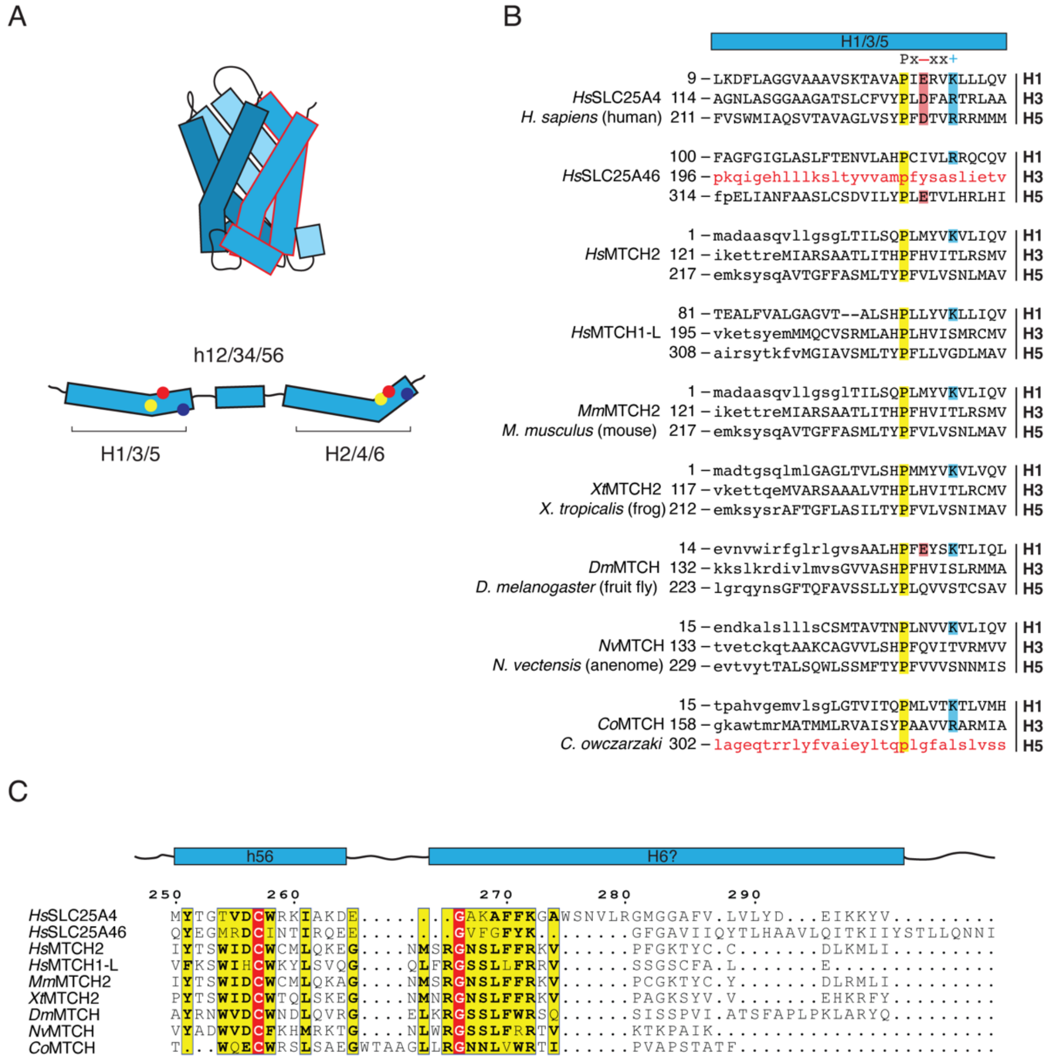

Fig. S1. Sequence analysis of conserved features across the MTCH insertase family. A) Schematic of the canonical SLC25 carrier fold highlighting the three pairs of TMs (six total) and their features that are conserved in SLC25 transporters. **B)** Hmmalign based sequence alignment of H1/3/5 of a canonical SLC25 transporter (the inner membrane localized SLC25A4), the OM localized SLC25A46, and 7 MTCH homologs. MTCH1 sequence numbering is based on the long isoform (MTCH1-L). Unaligned sections are shown in lowercase with no gaps. Highlighted columns correspond by color to the features shown in (A). The universally conserved proline, found in both the SLC25 and MTCH family is highlighted in yellow. The two charged residues shown in red and blue form a salt bridge that is important for stabilizing the cytosol-open conformation of canonical SLC25 carriers. The two repeats shown in red text were not recognized by hmmalign and were positioned based on the location of the conserved proline. **C)** MUSCLE-generated sequence alignment of the conserved C-terminal domain of the MTCH insertases that is not found in the SLC25 family. This domain is modeled with very low confidence by AlphaFold3 (*23*, *24*), and its conservation and positioning suggested it may be

functionally important. Therefore, an experimental structure was required to confidently model this region of the MTCH insertases. Labels above the alignment correspond to positions in canonical SLC25 transporters that typically encode the structural features h56 (a membrane-peripheral helix between H5 and H6) and H6.

Fig S2

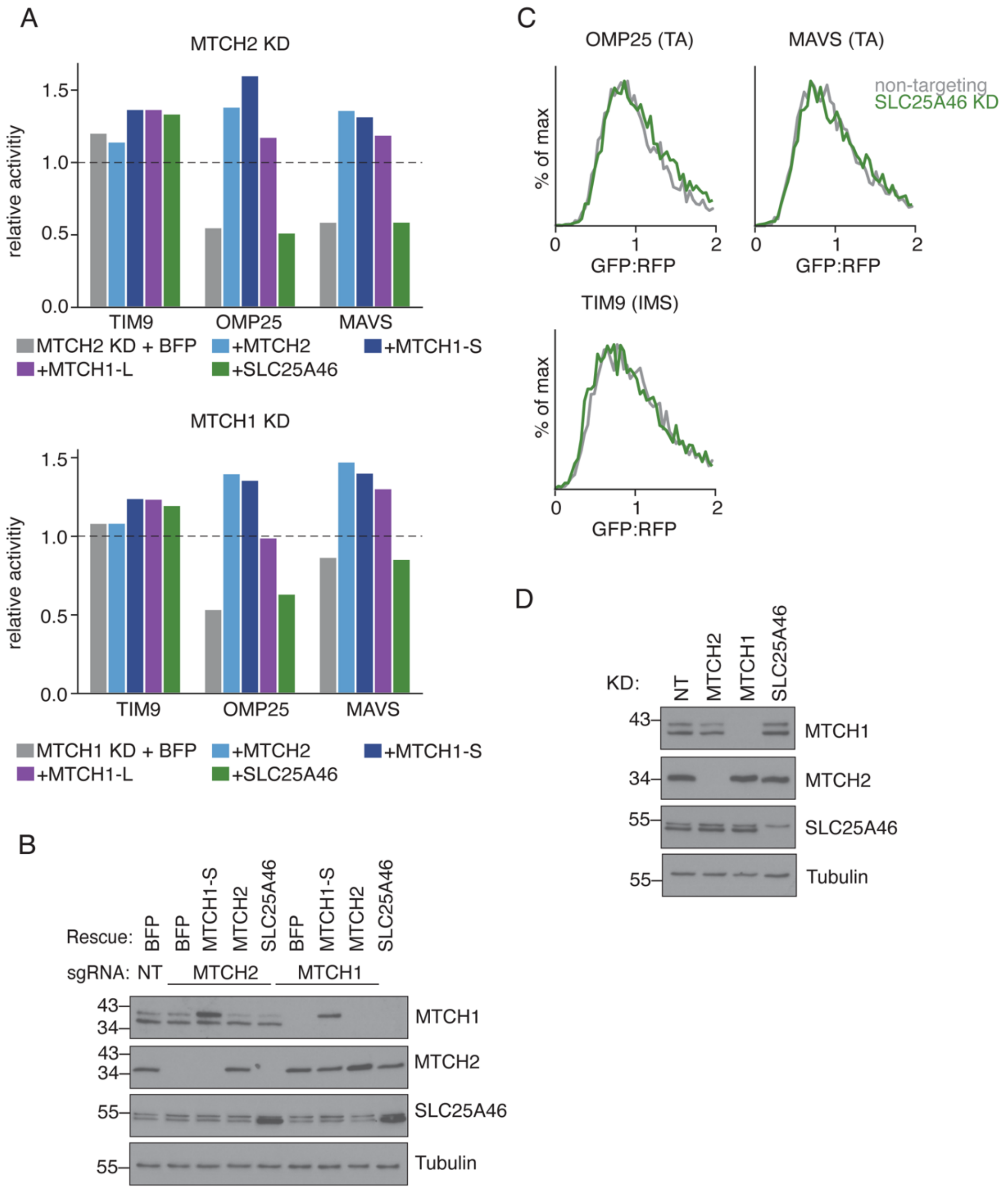

Fig. S2. OM insertion is a unique feature of MTCH1 and MTCH2 among human SLC25 carriers. A) (Top) To test whether the three SLC25 family members localized to the OM in human cells, MTCH1, MTCH2, and SLC25A46, are capable of mediating α-helical membrane protein insertion, we leveraged the ratiometric fluorescent reporter system described in Fig. 1B. Using human K562 CRISPRi cells constitutively expressing GFP(1-10) in the mitochondrial IMS, we depleted MTCH2 by transducing either a non-targeting (nt) or a MTCH2-targeting sgRNA. Rescue constructs encoding a BFP control, MTCH2-P2A-BFP, MTCH1-P2A-BFP (long [-L] and short [-S] splice isoforms), and SLC25A46-P2A-BFP were expressed to

determine if they could rescue the MTCH2 KD insertion phenotype on three GFP-11 fused reporters: the OM proteins OMP25 and MAVS, and a soluble IMS control, TIM9. Relative activity was calculated using the median GFP:RFP values for each rescue condition normalized to the median GFP:RFP values from nt cells (calculated as $GFP:RFP_{knockdown}/GFP:RFP_{nt}$). (Bottom) The same rescue experiment was performed with K562 CRISPRi cells expressing a MTCH1-targetting sgRNA. MTCH1 and MTCH2 are both capable of rescuing the phenotype when the other is depleted, while SLC25A46 is not. **B)** Immunoblotting was performed to confirm the MTCH1 and MTCH2 knockdown efficiency and the expression of the rescue constructs (MTCH1-S, MTCH2, and SLC25A46) shown in (A) relative to a housekeeping control (tubulin). While both MTCH1 isoforms are expressed in K562, the MTCH1-S isoform appears to be the dominant one. **C)** As in (A) except testing the effect of depletion of SLC25A46 on OM insertion of the indicated reporter constructs. Consistent with the results in (A) knockdown of SLC25A46 had no effect on OM insertion. **D)** Immunoblotting to show efficient depletion of SLC25A46 for the experiment in (C).

Fig S3

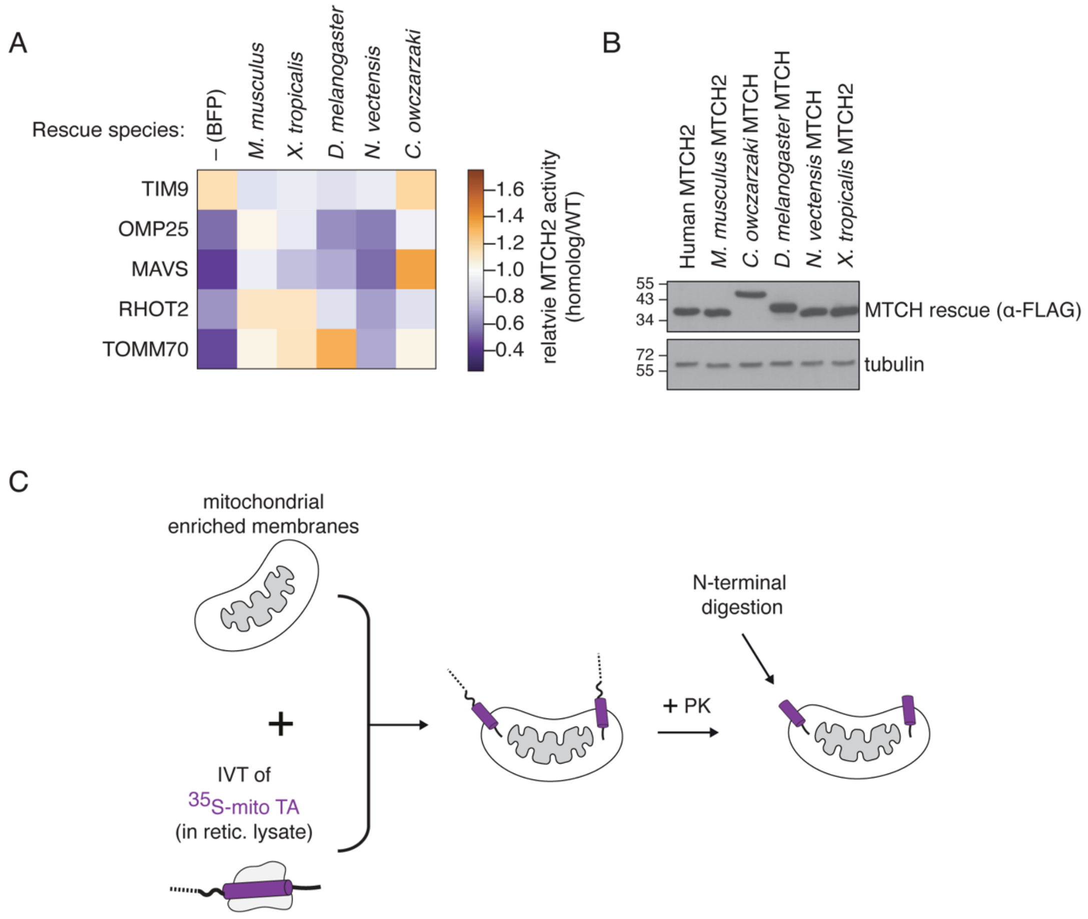

Fig. S3. **MTCH homologs are highly conserved across holozoans and all function as OM insertases. A)** The activity of MTCH homologs from *M. musculus, C. owkzarzaki, D. melanogaster*, *N. vectensis*, and *X. tropicalis* were assessed to determine if OM insertion of α-helical proteins is a conserved function across the MTCH family. The ratiometric reporter system was used to measure the insertion of five OM reporter proteins (OMP25, MAVS, RHOT2, FUNDC1, TOMM70) and the soluble IMS control, TIM9, in human K562 MTCH2 KO cells. Rescue constructs encoding either a BFP control, human (*H. sapiens*) MTCH2, or five different MTCH homologs, all 3xFLAG-tagged, were transduced along with the indicated reporters. 48 hours after transduction, reporter insertion was measured using flow cytometry. Displayed is the insertion activity of each homolog relative to the human MTCH2 rescue, calculated as: median $GFP:RFP_{homolog}$/median $GFP:RFP_{human}$. The resulting values are displayed as a heat map with orange representing increased rescue activity compared to human MTCH2, white representing equal rescue activity, and blue representing reduced or no rescue activity. Representing a remarkable level of conservation, the majority of MTCH homologs, even from distant organisms such as the single-celled *C. owkzarzaki,* are capable of rescuing the MTCH2 knockout phenotype in human cells for several human substrates. Data for select homologs are plotted as histograms in Fig. 1C. **B)** Immunoblotting was performed to confirm expression of the MTCH homologs shown in (A), which was normalized for transduced cells using % BFP positive cells. **C)** Schematic of an in vitro insertion assay into purified mitochondria as previously described (*8*,

78), used to generate the data displayed in Fig. 1D. An ^{35}S-methionine -labeled OM tail-anchored (TA) substrate is translated in rabbit reticulocyte lysate. Translation is terminated by incubation with puromycin, followed by incubation with mitochondria purified from K562 cells expressing a particular MTCH family member. Insertion of the OM substrate in the correct topology is measured by a protease protection assay, in which mitochondria are incubated with proteinase K (PK), followed by affinity purification of the protease protected fragment using a C-terminal 6x-HIS tag. Insertion is quantified by SDS-PAGE and autoradiography.

Fig S4

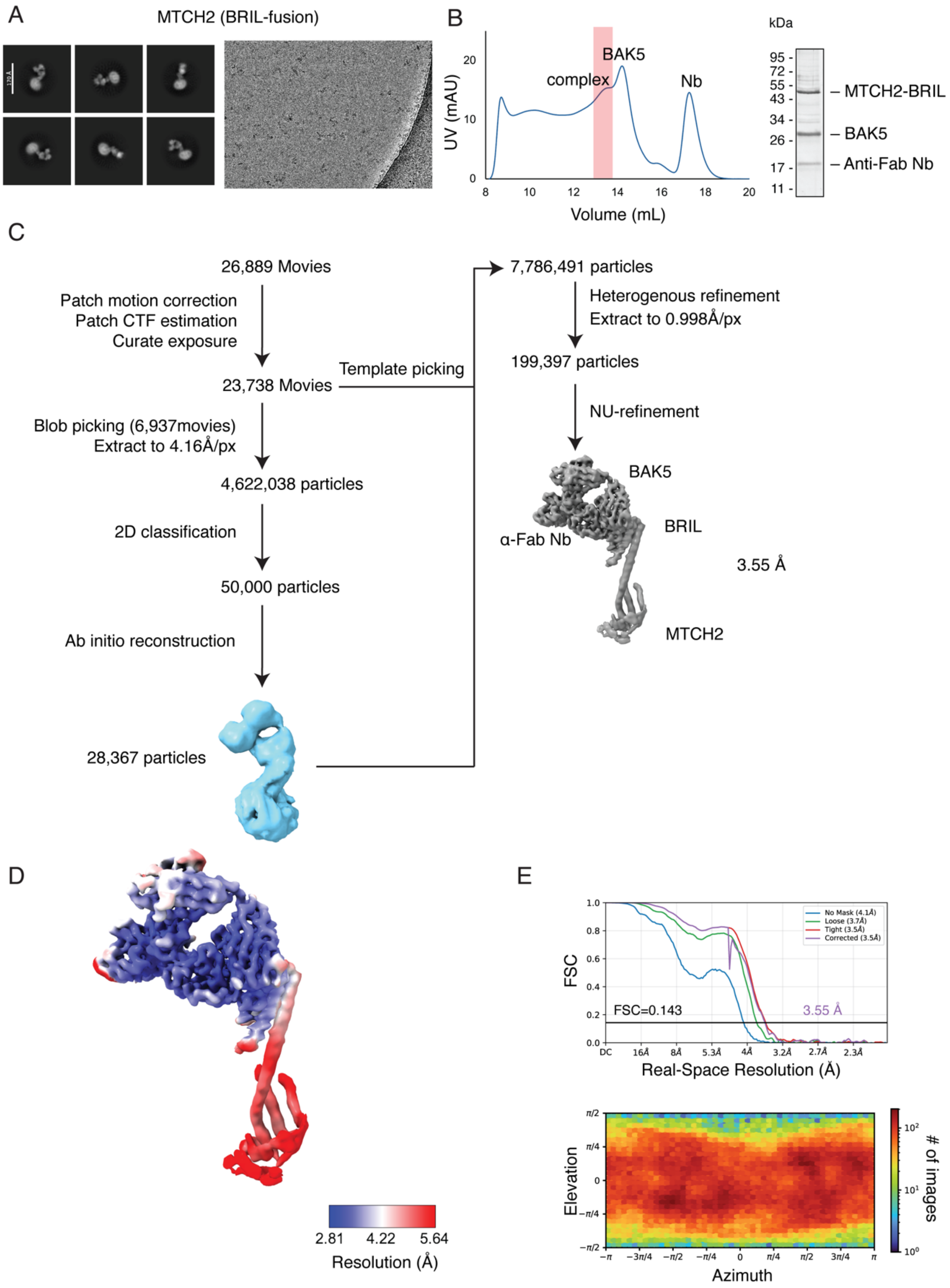
A
MTCH2 (BRIL-fusion)
B
UV (mAU)
Volume (mL)
complex
BAK5
Nb
kDa
95
72
55
43
34
26
17
11
– MTCH2-BRIL
– BAK5
– Anti-Fab Nb
C
26,889 Movies
Patch motion correction
Patch CTF estimation
Curate exposure
23,738 Movies
Template picking
Blob picking (6,937movies)
Extract to 4.16Å/px
4,622,038 particles
2D classification
50,000 particles
Ab initio reconstruction
28,367 particles
7,786,491 particles
Heterogenous refinement
Extract to 0.998Å/px
199,397 particles
NU-refinement
BAK5
BRIL
α-Fab Nb
3.55 Å
MTCH2
D
2.81
4.22
5.64
Resolution (Å)
E
FSC
No Mask (4.1Å)
Loose (3.7Å)
Tight (3.5Å)
Corrected (3.5Å)
FSC=0.143
3.55 Å
Real-Space Resolution (Å)
Elevation
Azimuth
# of images

Fig. S4. Cryo-EM data processing workflow for MTCH2(BRIL)•BAK5•α-Fab Nb. A) (left) Representative 2D class averages of the MTCH2(BRIL)•BAK5•α-Fab Nb complex, (right) and a representative micrograph from the data collection. **B)** (left) Size exclusion chromatography trace with the peak corresponding to the MTCH2 (BRIL)•BAK5•α-Fab Nb complex highlighted in red. (right) Pooled peak fractions were analyzed by SDS-PAGE and SYPRO Ruby stain. **C)** Data processing pipeline using cryoSPARC v4.7.0. Micrographs were curated and a subset was used for blob picking. The resulting particle set was extracted at 4.16 Å/pixel and subject to iterative rounds of 2D classification. Particles from selected 2D classes were used in ab-initio reconstruction to generate 3D volumes. The volume representing a Fab attached to a detergent micelle was selected and used to generate 2D templates for template picker. After re-picking particles from the entire dataset, the resulting particle set was combined with the initial set and processed through iterative rounds of heterogenous refinement, yielding a final particle set of 199,397 particles. The particles were re-extracted at 0.998 Å/pixel and used to generate a final map in a non-uniform refinement job. **D)** Cryo-EM density map of MTCH2 (BRIL)•BAK5•α-Fab Nb colored by local resolution in Å as calculated by cryoSPARC v4.7.0. **E)** Gold Standard Fourier Shell Correlation (GSFSC) curves (top) of the MTCH2 (BRIL)•BAK5•α-Fab Nb complex map with a loose mask (green), tight mask (red), corrected mask (purple) or no mask (blue), and particle Euler angle distribution (bottom). A nominal resolution of 3.55 Å was determined for this map based off of an FSC cutoff of 0.143. Both plots were generated from the local refinement job in cryoSPARC v4.7.0.

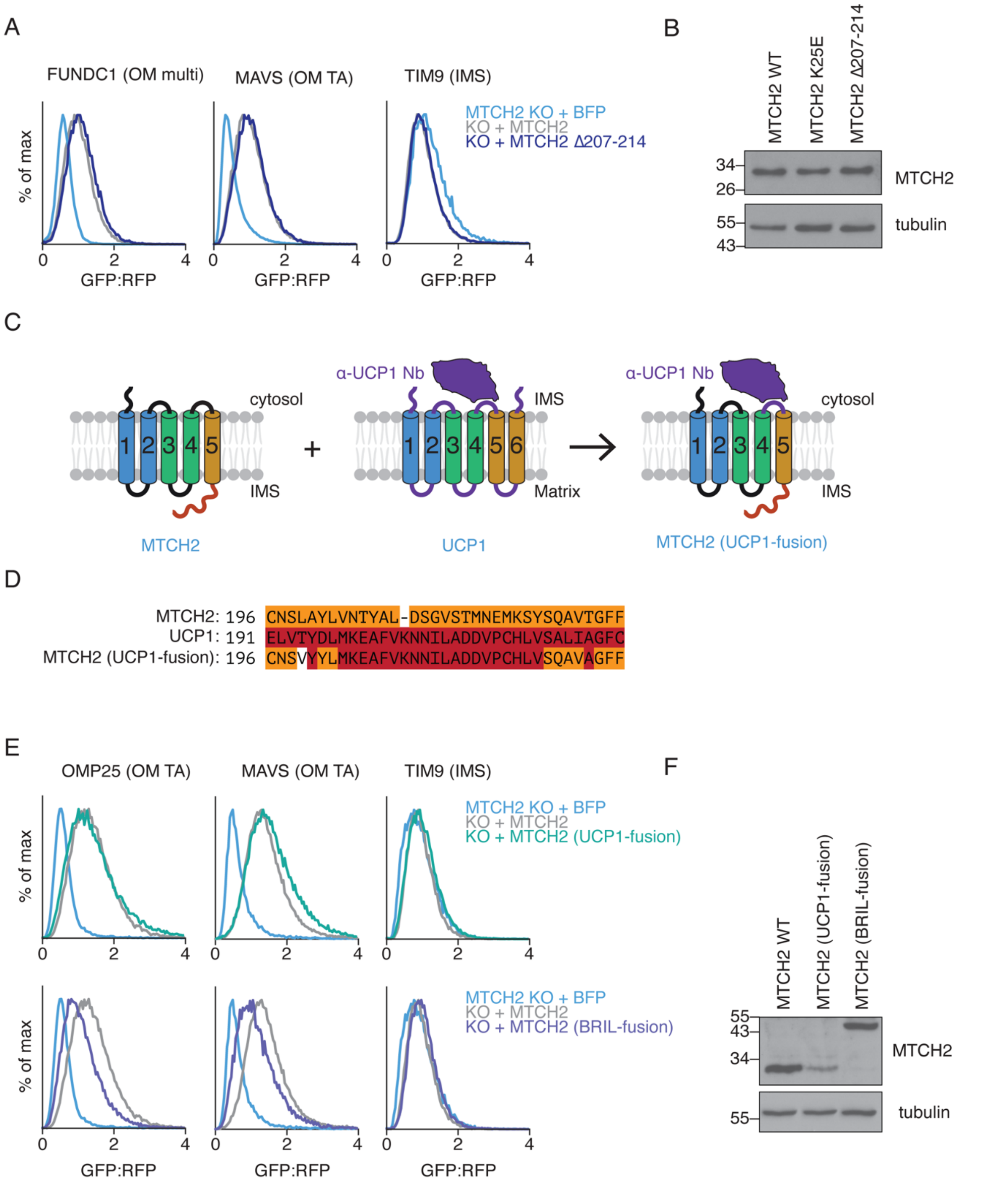

Fig. S5. A universal strategy for determination of SLC25 structures including the diverged MTCH family homologs. A) The functional importance of the MTCH2 loop 4/5 was tested using a deletion mutant. The ratiometric reporter system was used to measure the insertion of two OM proteins, FUNDC1 (a multi-pass protein) and MAVS (a TA), and the soluble IMS control TIM9 in MTCH2 KO cells. Rescue constructs encoding either a BFP control, WT MTCH2, or a MTCH2 variant with residues 207-214 in loop 4/5 replaced with a glycine-serine

linker of equivalent length were expressed to determine if the loop 4/5 mutant could rescue the MTCH2 KO phenotype. MTCH2 activity is plotted as a histogram. Loop 4/5 mutants were equally as active as WT MTCH2, suggesting that loop 4/5 is dispensable for its insertase activity. **B)** Immunoblotting analysis of MTCH2 was performed to verify expression of the constructs from (A). To account for differences in transduction efficiency, samples are normalized to % BFP positive cells. Expression of the loop4/5 mutant is displayed relative to that of other MTCH2 mutations that do not cause destabilization. **C)** Schematic of the strategy used to determine the structure of human MTCH2 using an α-UCP1 nanobody. This design strategy exploits the fact that MTCH2 and UCP1 are both SLC25 carriers and thus have a similar fold. A previously characterized α-UCP1 nanobody (pMb65) recognizes a small number of sequential UCP1 residues within the IMS-facing loop between TM4 and TM5 (*29*), a region known to be dispensable for function in other SLC25 carriers (*32*) as well as MTCH2 (A). To generate a MTCH2 variant capable of binding pMb65, several residues in MTCH2 loop 4/5 were replaced with those from UCP1 (shown in purple). **D)** Sequence alignment showing the mutations to WT MTCH2 within cytosolic loop 4/5 made to allow the α-UCP1 antibody to recognize MTCH2. Human MTCH2 residues 203-222 were replaced with UCP1 residues 198-218, and three point mutations, L199V, A200Y, and T226A, were introduced to recreate the α-UCP1 nanobody (pMb65) binding epitope Residues of the MTCH2 (UCP1) originated from human MTCH2 are shown in orange, UCP1 residues in red, and stabilizing mutations are left uncolored. **E)** The activity of the MTCH2 (UCP1) and (BRIL) variants were assessed to determine if the modifications interfered with insertase function. Insertion of two OM proteins, OMP25 and MAVS, and the soluble IMS control TIM9 were tested in human K562 MTCH2 KO cells. Rescue construct encoding either a BFP control, WT MTCH2, MTCH2 (UCP1), or MTCH2 (BRIL) were expressed to determine if the variants designed for structure determination could rescue the MTCH2 KO phenotype. Both MTCH2 fusions mediate insertion of the OM reporters as well as WT suggesting the mutations to enable structure determination do not affect MTCH2 activity in cells**. F)** Immunoblotting analysis of MTCH2 was performed to verify the expression of the constructs from (E). To account for differences in transduction efficiency, samples are normalized to the % BFP positive cells.

Fig S6

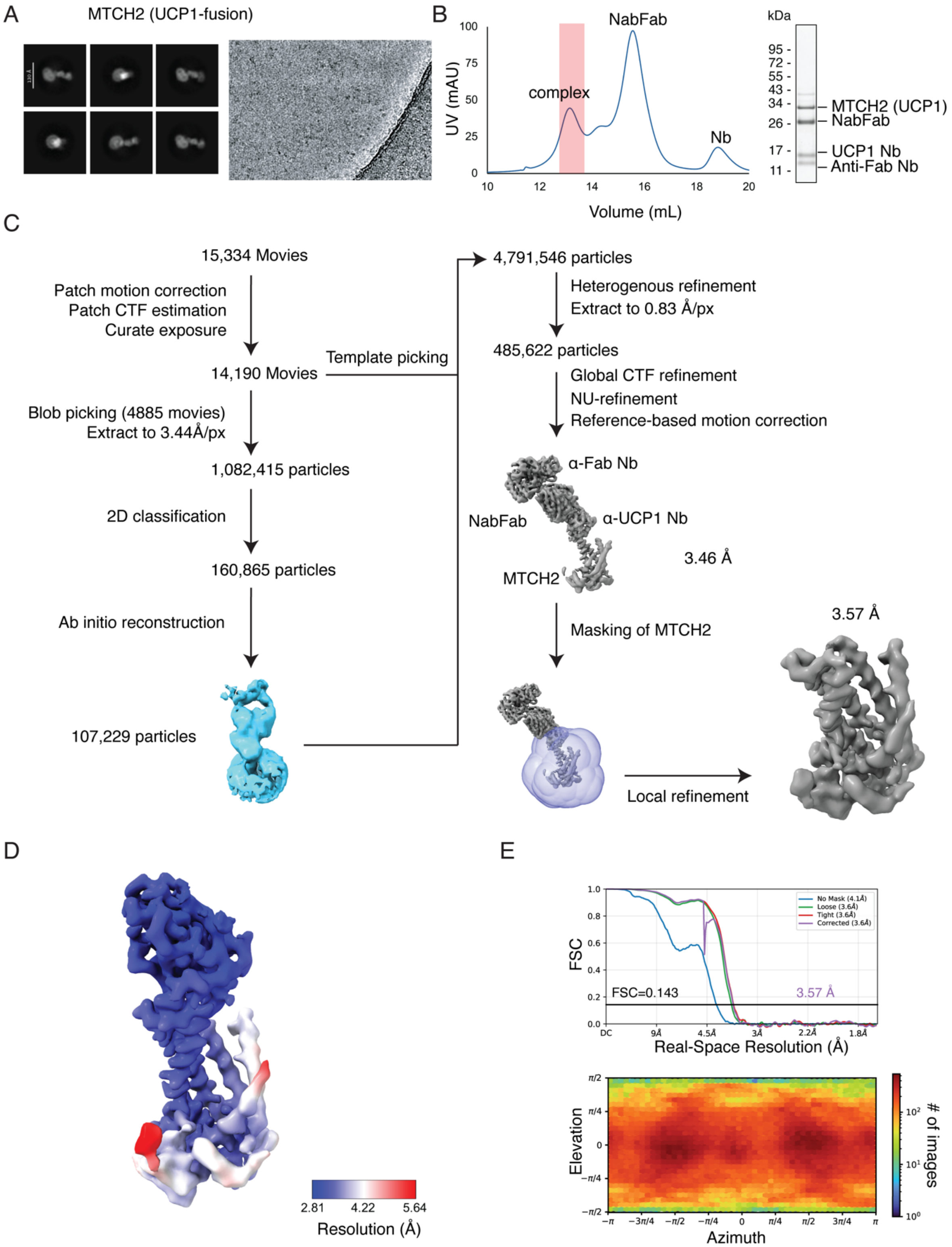
A
MTCH2 (UCP1-fusion)
B
NabFab
complex
Nb
UV (mAU)
Volume (mL)
kDa
MTCH2 (UCP1)
NabFab
UCP1 Nb
Anti-Fab Nb
C
15,334 Movies
Patch motion correction
Patch CTF estimation
Curate exposure
14,190 Movies
Template picking
Blob picking (4885 movies)
Extract to 3.44Å/px
1,082,415 particles
2D classification
160,865 particles
Ab initio reconstruction
107,229 particles
4,791,546 particles
Heterogenous refinement
Extract to 0.83 Å/px
485,622 particles
Global CTF refinement
NU-refinement
Reference-based motion correction
α-Fab Nb
NabFab
α-UCP1 Nb
MTCH2
3.46 Å
Masking of MTCH2
3.57 Å
Local refinement
D
2.81
4.22
5.64
Resolution (Å)
E
FSC
FSC=0.143
3.57 Å
Real-Space Resolution (Å)
Elevation
Azimuth
# of images

Fig. S6. Cryo-EM data processing workflow for MTCH2•α-UCP1 Nb•NabFab•α-Fab Nb. **A)** (left) Representative 2D class averages of the MTCH2•α-UCP1 Nb•NabFab•α-Fab Nb tetrameric complex, (right) and a representative micrograph from the data collection. **B)** (left) Size exclusion chromatography trace with the peak corresponding to the MTCH2•α-UCP1 Nb•NabFab•α-Fab Nb complex highlighted in red. (right) Pooled peak fractions were analyzed by SDS-PAGE and SYPRO Ruby stain (right). **C)** Data processing pipeline of MTCH2 using cryoSPARC v4.7.0. Micrographs were curated and a subset was used for blob picking. The resulting particle set was extracted at 3.44 Å/pixel and subject to iterative rounds of 2D classification. Particles from selected 2D classes were used in ab-initio reconstruction to generate 3D volumes. The volume representing a Fab attached to a detergent micelle was selected and used to generate 2D templates for template picker. After re-picking particles from the entire dataset, the resulting particle set was combined with the initial set and processed through iterative rounds of heterogenous refinement, yielding a final particle set of 485,622 particles. The particles were re-extracted at 0.83 Å/pixel and subject to global CTF refinement and reference-based motion correction. The final map was obtained from a non-uniform refinement job followed by the local refinement job using a mask on MTCH2 only. **D)** Cryo-EM density map of MTCH2•α-UCP1 Nb colored by local resolution in Å as calculated by cryoSPARC v4.7.0. **E)** Gold Standard Fourier Shell Correlation (GSFSC) curves (top) of the MTCH2•α-UCP1 Nb map with a loose mask (green), tight mask (red), corrected mask (purple) or no mask (blue), and particle Euler angle distribution (bottom). A nominal resolution of 3.57 Å was determined for this map based off of an FSC cutoff of 0.143. Both plots were generated from the local refinement job in cryoSPARC v4.7.0.

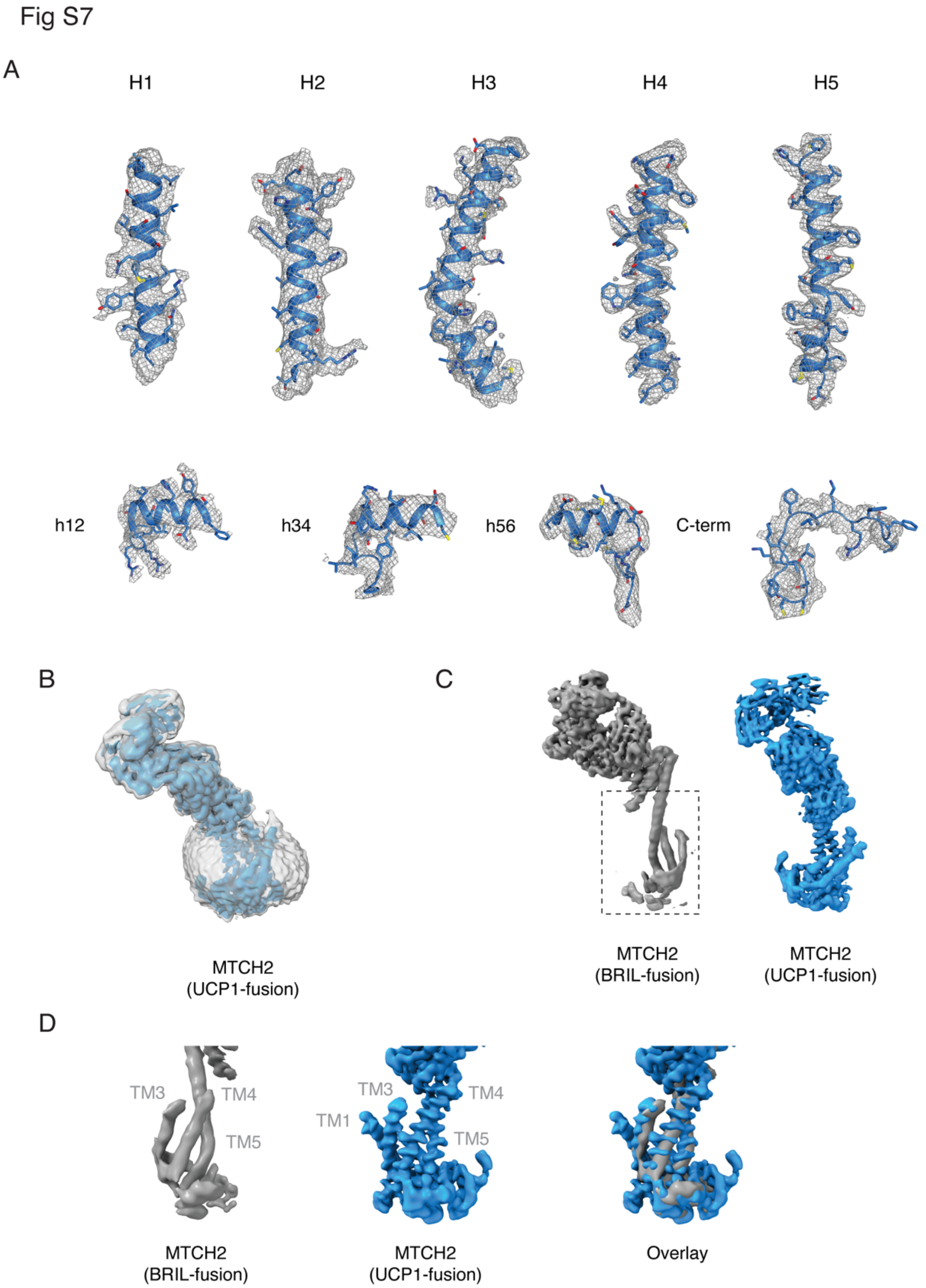

Fig. S7. Cryo-EM map assessment and MTCH2 fusion constructs comparison. A) Cryo-EM density map of MTCH2 (UCP1) displayed as mesh around the 5 TM helices, and helical linker

h12, h34, and h56, and the C-terminus, highlighting overall map quality, with density for at least one or two prominent side chains visible on each helix. **B)** Low-threshold (gray, transparent) overlayed with high-threshold (blue, solid) cryo-EM density map of MTCH2 •α-UCP1 Nb•NabFab•α-Fab Nb complex highlighting MTCH2 embedded into UDM detergent micelle. **C)** Comparison between cryo-EM density map of MTCH2 (BRIL)•BAK5•α-Fab Nb complex (left, gray) and MTCH2•α-UCP1 Nb•NabFab•α-Fab Nb complex (right, blue). **D)** MTCH2-focused comparison of the EM density maps for MTCH2(BRIL) (gray) with that from MTCH2(UCP1) (blue). The overall conformation of MTCH2 in both fusions is similar, suggesting the fusions used for structure determination are not altering the conformation of MTCH2.

Fig S8

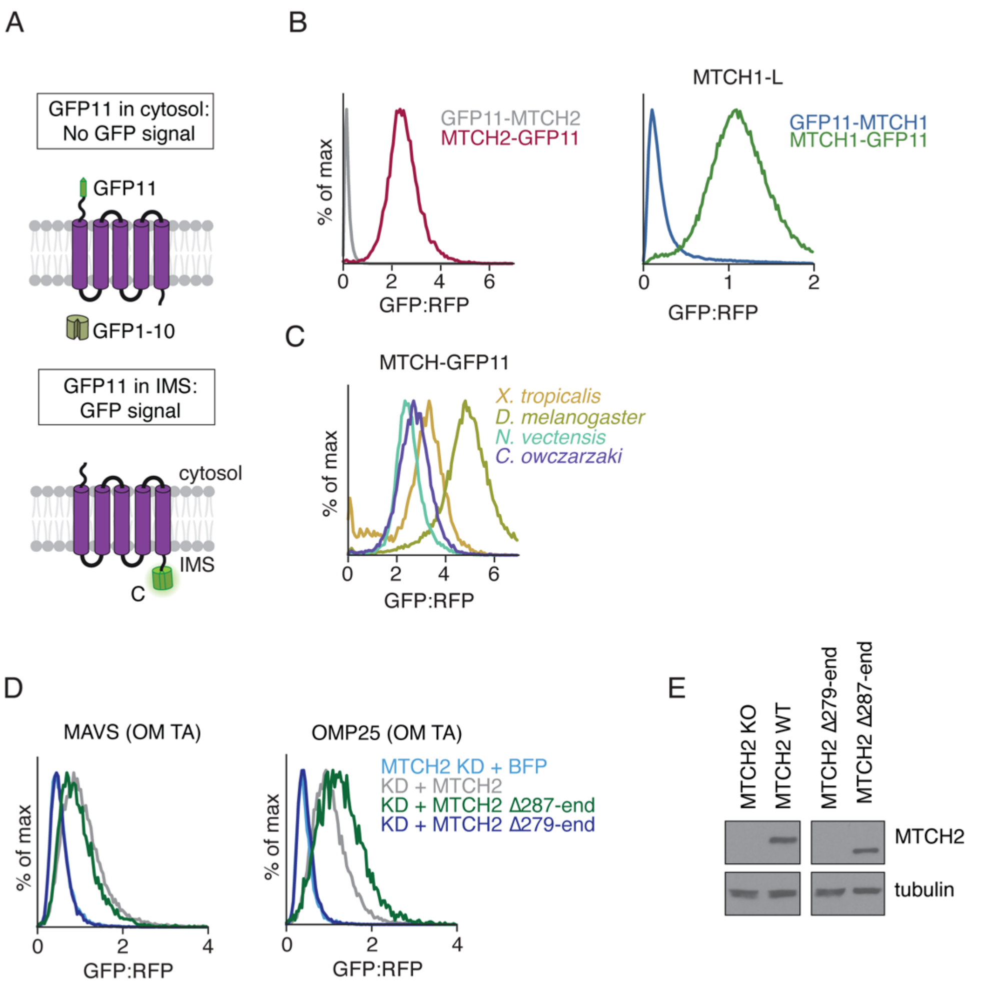

Fig. S8. MTCH homologs adopt a conserved 5 TM topology, which is specifically stabilized by a portion of the C-terminus. A) To experimentally confirm the topology of MTCH2 observed in the cryo-EM structure, we leveraged the split-GFP reporter system described in Fig. 1B. Here, GFP11 is fused to either the N- or C-terminus while GFP(1-10) is expressed in the IMS. If the GFP11-tagged terminus is in the IMS of the mitochondria, this will result in complementation and thus GFP fluorescence. Comparison of the GFP fluorescence of these two constructs, relative to a normalization marker (RFP), can be used determine protein topology. **B)** (top) Flow cytometry measurements of K562 IMS GFP(1-10) cells expressing RFP-P2A-tagged MTCH2 or MTCH1-L (long isoform, see fig. S2A) with GFP11 on either the N- (gray) or C-terminus (red). The C-terminal GFP11 tag has a much higher GFP:RFP for both proteins, consistent with positioning of their C-termini in the IMS, and their N-termini in the cytosol, as observed in the structure. Based on these data, together with the structure, we definitively concluded that MTCH2 contains five TMs. **C)** As in (B) for the indicated C-terminally GFP11 tagged MTCH homologs: *X. tropicalis*, *D. melanogaster, N. vectensis*, and *C. owczarzaki*. GFP complementation for all the homologs is similar to that for *H. sapiens* MTCH2-GFP11, suggesting the MTCH family all adopt same topology with 5 TMs and their C-termini in the IMS. **D)** Based on its position in the structure, we hypothesized that the C-terminal domain of

MTCH2 could be important for stabilizing its remaining 5 TMs, and thereby the hydrophilic groove created by loss of the 6th TM. To test this, we generated two truncations to the C-terminus of MTCH2 and tested their ability to mediate insertion of OMP25 and MAVS in K562 CRISPRi cells expressing a MTCH2 targeting sgRNA (MTCH2 KD). Rescue constructs encoding either a BFP control, WT MTCH2, or MTCH2 with C-terminal truncations to position 287 or 279 were expressed to determine if they could rescue the MTCH2 KO phenotype. **E)** Immunoblotting analysis of MTCH2 was performed to verify the expression of the constructs from (C). To account for differences in transduction efficiency, samples are normalized to the % BFP positive cells. While the truncation at residue 287 retained insertase activity and expressed as normal, the truncation at residue 279 was destabilized, indicating an important role for residues 279-286 in stabilizing the fold of MTCH2.

Fig S9

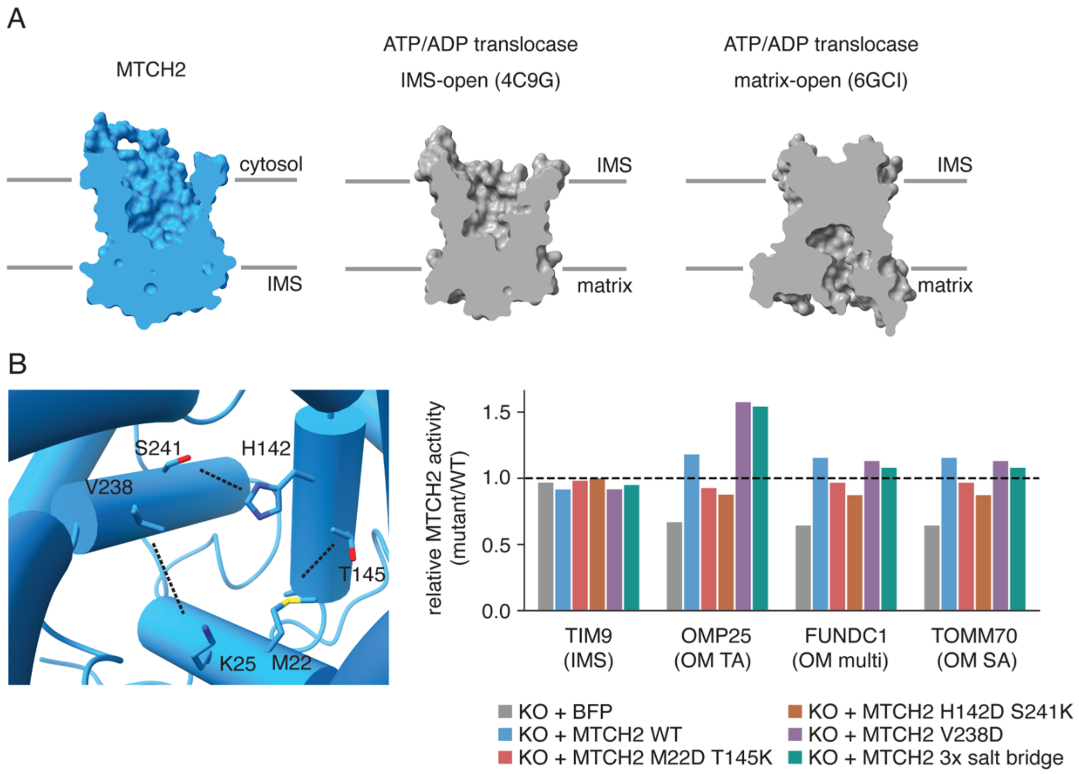

Fig. S9. MTCH2 exhibits a cytoplasm-open conformation without stabilizing salt bridges. **A)** Space-filling representation of the experimental MTCH2 structure and the SLC25 ATP/ADP translocase (AAC) in either an IMS-open (*26*) or matrix-open conformation (*25*) highlighting accessibility of the interior cavity from either side of the membrane. MTCH2 is stabilized in a conformation that is open towards the cytosol, consistent with its function in inserting OM proteins from a cytosolic substrate pool, despite the absence of canonical salt bridge-forming residues known to stabilize this conformation in other SLC25 carriers. **B)** Canonical SLC25s contain several conserved salt bridges between TM helices that enable the conformational changes required for the alternating access model of solute transport. The majority of these salt bridges are no longer found in the MTCH family of insertases, and we wondered if the loss of these interactions were important for MTCH2's insertase function. Therefore, we tested the effect of restoring these SLC25 salt bridges to MTCH2 on its insertion activity. (left) MTCH2 side chains are shown at positions where conserved salt bridge forming residues are normally located. (right) Insertion of three MTCH2-dependent OM proteins, OMP25 and FUNDC1, and RHOT1, and the soluble IMS control TIM9 were measured in MTCH2 KO cells. Rescue constructs encoding either a BFP control, WT MTCH2, MTCH2 mutants with a single salt bridge restored at one of three positions, or a MTCH2 mutant with all three salt bridges restored were expressed to determine if they could rescue the MTCH2 KO phenotype. The activity of each MTCH2 mutant relative to WT MTCH2 (calculated as median $GFP:RFP_{mut}$/median $GFP:RFP_{wt}$) was determined. The presence of salt bridges either does not alter MTCH2 insertase function, or potentially enhances it in one position, V238. We therefore concluded that loss of

these salt bridges was not a critical evolutionary adaptation of the MTCH family during their evolution from an SLC25 transporter.

Fig S10

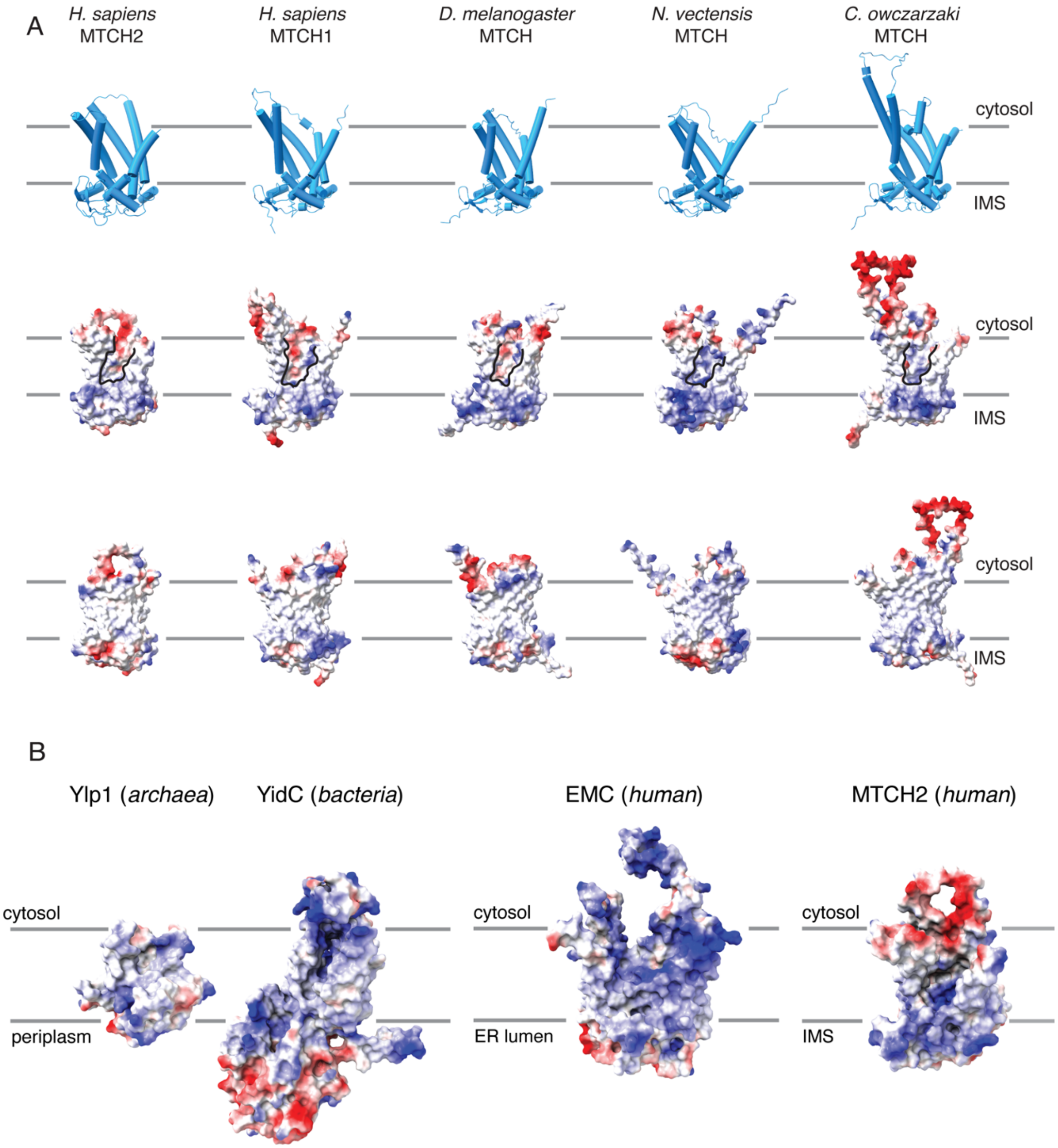

Fig. S10. Identification of conserved features of the MTCH family of insertases. A) Comparison (from left to right) between our experimental MTCH2 model, and AlphaFold3-predicted (*23*, *24*) models of human MTCH1, and MTCH homologs from *Drosophila melanogaster*, *Nematostella Vectensis*, and *Capsaspora Owczarzaki*. Cartoon rendered models are shown on top and surface-mapped coulombic electrostatic potential (calculated with ChimeraX) is shown in the middle (front-facing) and bottom rows (180° rotated). All homologs have a comparable fold. We have also experimentally confirmed that they each contain 5 TMs, can express in the OM of human cells (fig. S8A-C), and adopt the same topology as human

MTCH2 with their C-termini in the IMS. The loss of TM6, when compared to an SLC25, results in a prominent, lipid-accessible hydrophilic groove, which is a conserved feature across the MTCH family of insertases. Structural modeling does not accurately predict the conformation of the C-terminal domain of human MTCH2, so it is not possible to interpret the structural conservation of this feature. However, sequence alignments in fig. S1C, suggest that this domain is also a conserved feature across the MTCH family of insertases that is not found in canonical SLC25s. Residues 1-70 of MTCH1, which are predicted with low confidence were omitted for simplicity. **B)** Space-filling representation of human MTCH2 colored by electrostatic potential in comparison to other evolutionarily unrelated insertases. Highlighted is the presence of a conserved lipid-exposed hydrophilic groove, which is a unifying feature defining diverse insertase folds. Coulombic electrostatic potential was calculated using ChimeraX and mapped onto the surface of Ylp1 (PDB: 5C8J; (*90*)), YidC (PDB: 6Y86; (*91*)), EMC (PDB: 8S9S; (*45*)), and MTCH2.

Fig S11

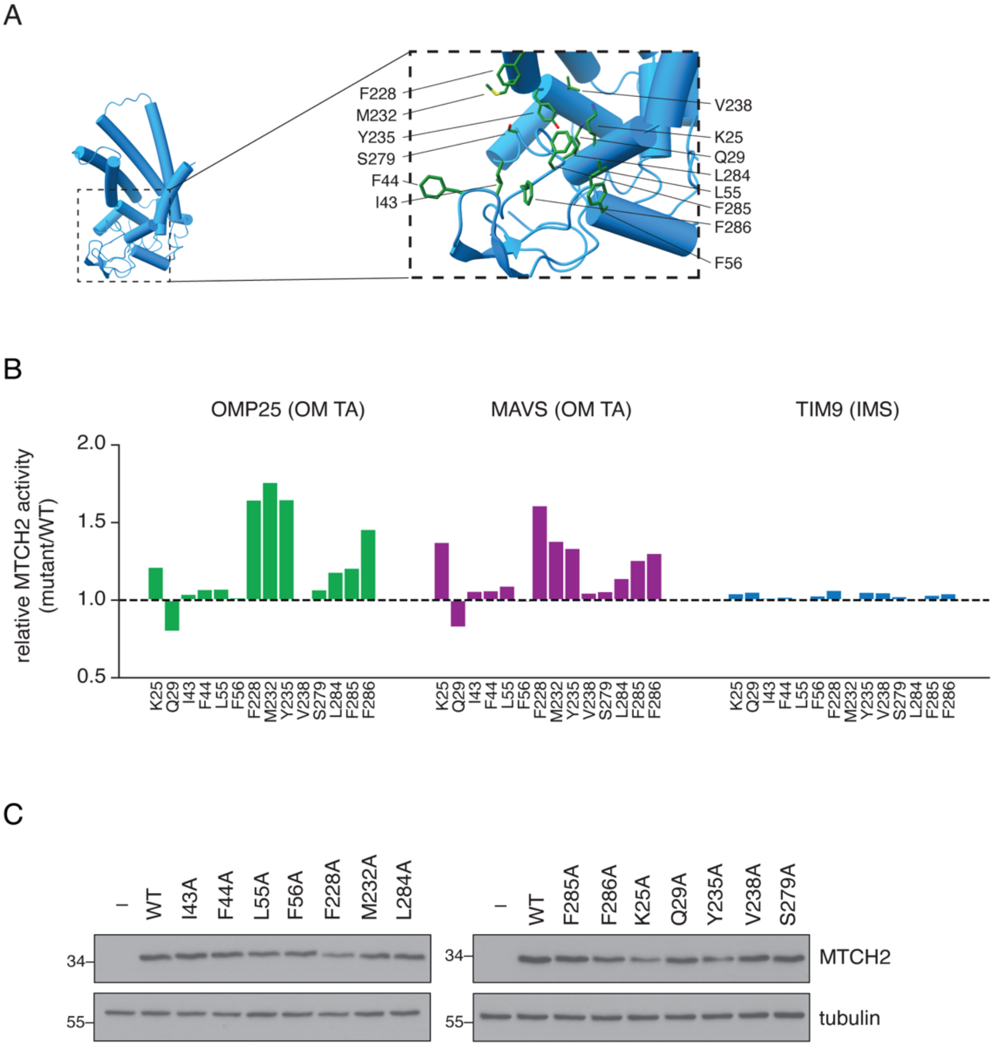

Fig. S11. MTCH2 activity is attenuated by residues lining its hydrophilic groove. A) The most distinctive feature of the MTCH homologs relative to other SLC25 family members is the absence of TM6, leaving a hydrophilic groove accessible to the bilayer. Given the functional significance of these types of hydrophilic grooves in other insertase families, we hypothesized that the amino acids that define this region would be important for MTCH2's insertase activity. To assess the importance of amino acids in this region, 14 sites (K25, Q29, I43, F44, L55, F56, F228, M232, Y235, V238, S279, L284, F285, F286) were chosen for initial focus in structure-function experiments. **B)** Alanine scanning mutagenesis was performed on MTCH2 at the indicated 14 sites in and adjacent to the base of the hydrophilic groove. The ratiometric reporter system was used to measure the insertion of two OM proteins, OMP25 and MAVS, and the soluble IMS protein control, TIM9, in MTCH2 KO cells. Rescue constructs encoding WT MTCH2 and alanine mutations at each of the 14 positions were expressed to determine their effect on OM insertion. The activity of each MTCH2 mutant relative to WT MTCH2 (calculated as median $GFP:RFP_{mut}$/median $GFP:RFP_{wt}$) was determined. Notably many of the mutations appear to increase insertion activity. This data is mapped onto the MTCH2 structure in Fig. 3B.

C) Immunoblotting of the MTCH2 mutants in (B) was performed to exclude potential effects of protein expression and stability on activity. To control for differences in transduction efficiency, samples were normalized to % BFP positive cells.

Fig S12

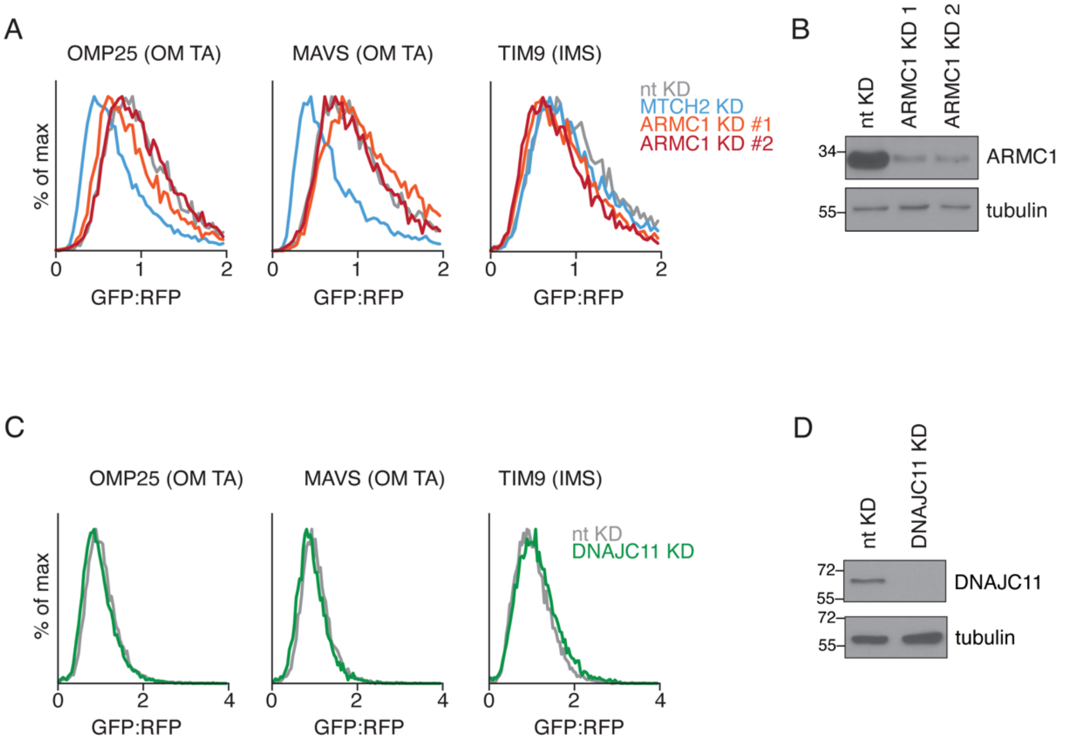

Fig. S12. In K562 cells, MTCH2 activity is not attenuated by its two known binding partners, DNAJC11 and ARMC1. A) One potential explanation for MTCH2's apparent attenuation, could be binding of an inhibitory regulatory molecule. We assessed whether this could be the case for one of the known binding partners of MTCH2, ARMC1 (*38*). To test whether depletion of ARMC1 in K562 cells would affect MTCH2's insertase function, we measured the insertion of the indicated OM proteins and soluble IMS control, in K562 CRISPRi cells expressing either a non-targeting (nt) or a ARMC1-targeting sgRNA. Results are normalized to nt, and displayed as a histogram. The results suggest that MTCH2 function does not depend on ARMC1 in K562 cells, however this may not be the case in other cell lines. **B)** Immunoblotting was performed to measure ARMC1 depletion in (A). **C,D)** As in (A, B), except for DNAJC11, since it is another MTCH2-interaction partner that could regulate its activity (*38*).

Fig S13

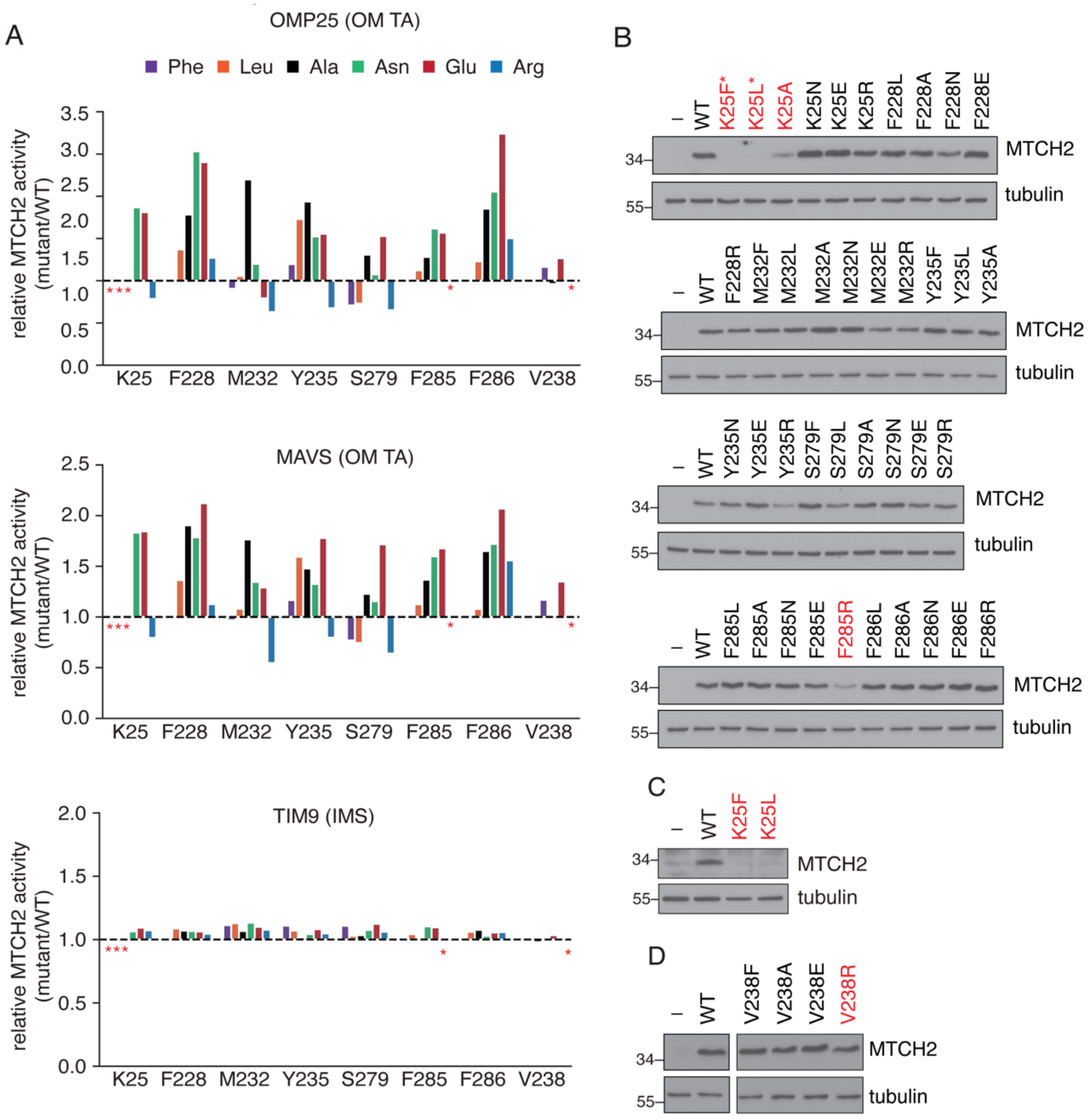

Fig. S13. Systematic mutagenesis of MTCH2 to identify hyperactivity patterns. A) A subset of 8 residues (K25, F228, M232, Y235, S279, F285, and F286, V238) from the mutants shown in fig. S11 were chosen for further interrogation. After observing hyperactivity from the alanine scanning analysis (fig. S11), we hypothesized that certain amino acids may have an inhibitory effect at these positions. To test this hypothesis, we mutated each position to a broader range of amino acids (Phe, Leu, Ala, Asn, Glu, and Arg) and tested the effects of these mutations on insertion of two OM proteins, OMP25 and MAVS, and a soluble IMS control, TIM9, in MTCH2 KO cells. Rescue constructs encoding WT MTCH2 and a total of 42 mutants were expressed to determine their effect on OM insertion. Relative activity was determined for each mutant compared to WT MTCH2. Mutations clearly affecting stability, as shown in B-D), were excluded from analysis at positions marked with a red asterisk. V238 mutational analysis was performed in a separate experiment where only Phe, Ala, Arg and Glu mutants were tested. **B)**

Immunoblotting of the MTCH2 mutants in (A) was performed, controlling for differences in transduction efficiency by normalizing to % BFP positive cells. Red font is used to denote cases where mutations are destabilizing, markedly decreasing MTCH2 expression (K25F, K25L, K25A, F285R). We concluded that we cannot interpret the effects of these mutations on MTCH2 activity. *K25F and K25L were not normalized for % BFP positivity in this set of immunoblots due to their toxicity during lentiviral production and thereby poor transduction efficiency. **C)** Immunoblotting with K25F and K25L mutants from (A) after normalizing to % BFP positive cells, confirming lack of expression. **D)** Immunoblotting of MTCH2 V238 mutants in (A) was performed, controlling for differences in transduction efficiency by normalizing to % BFP positive cells. Individual lanes were cropped from the same immunoblot at the same exposure level.

Fig S14

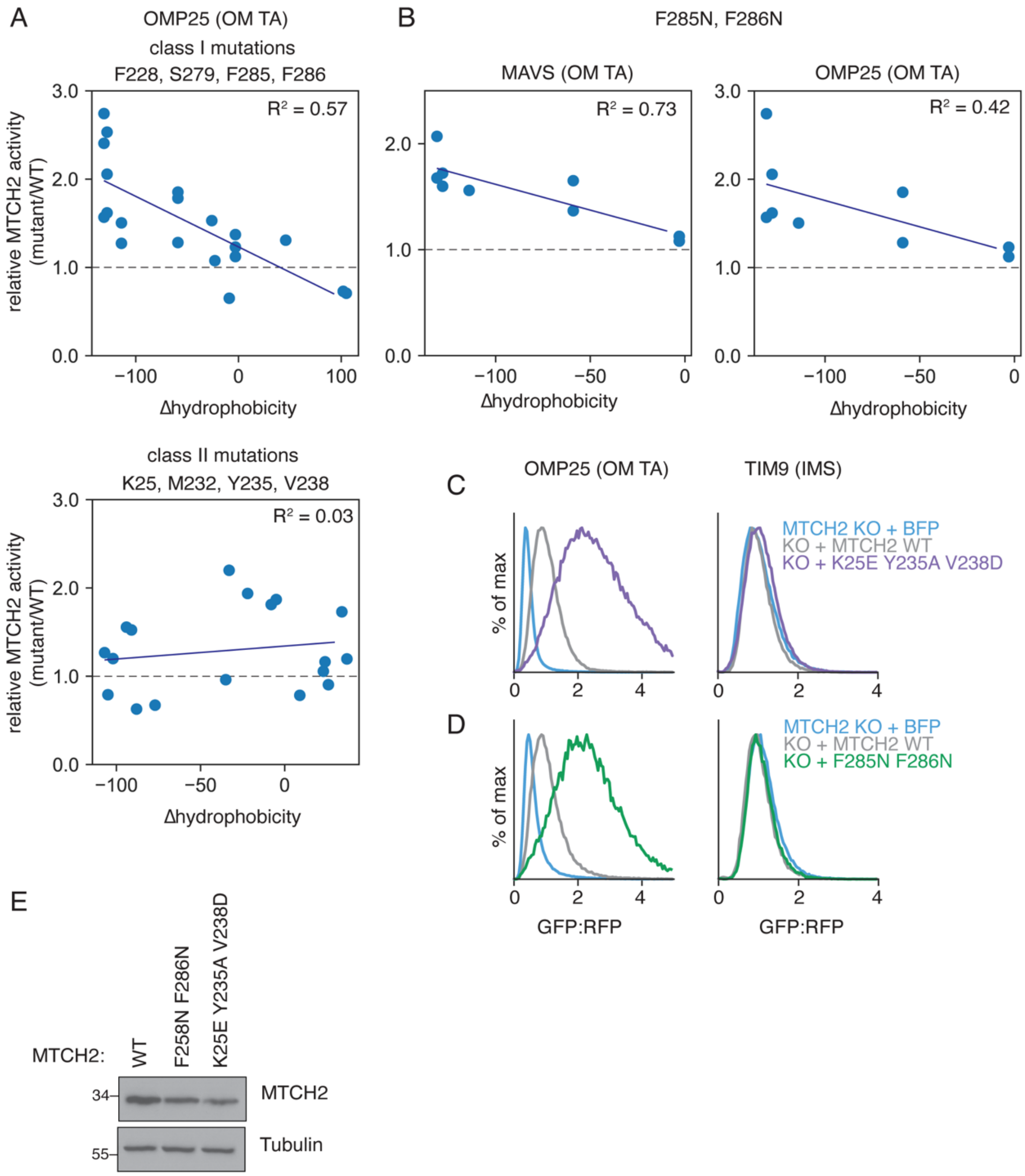

Fig. S14. Two distinct classes of MTCH2 mutations result in hyperactivation. A) Additional analysis related to Fig. 3D. (top) Data summarizing the effect of MTCH2 mutations (Phe, Leu, Ala,Gln, Glu, Arg) at 4 class I positions (F228, S279, F285, F286) on OMP25 insertion (full mutation panel shown in fig. S13A). The relative MTCH2 activity ($GFP:RFP_{mut}/GFP:RFP_{wt}$) plotted against the change in hydrophobicity of the mutated amino acid (ΔHydrophobicity=$hydrophobicity_{aa\ mut}$-$hydrophobicity_{aa\ wt}$), as determined using the indicated hydrophobicity scale (*56*). (bottom) data shown for 4 class II positions (K25, M232, Y235, V238). **B)** (left) Data summarizing the effect of mutations (Phe, Leu, Ala,Gln, Glu, Arg) at 2 positions (F285, F286) on MAVS insertion by MTCH2 mutants (full mutation panel shown in

fig. S13A). The relative MTCH2 activity ($GFP:RFP_{mut}/GFP:RFP_{wt}$) plotted against the change in hydrophobicity of the mutated amino acid (ΔHydrophobicity=$hydrophobicity_{aa\ mut}$-$hydrophobicity_{aa\ wt}$), as determined using a published hydrophobicity scale (*56*). (right) Effects for the same mutations on OMP25 insertion. **C-D)** Additional activity measurements for class I and class II combination mutants corresponding to data shown in Fig. 4A,C. We measured the insertion of indicated OM proteins and soluble IMS control, in MTCH2 KO cells. Rescue constructs encoding a BFP control, WT MTCH2, and either the class II combination mutant $MTCH2^{F285N,F286N}$ in (D) or the class I combination mutant $MTCH2^{F285N,F286N}$ in (E) were expressed to determine their effect on OM insertion. Results are normalized to MTCH2 WT and displayed as histograms. **E)** Immunoblotting of the MTCH2 mutants in (D-E) was performed, controlling for differences in transduction efficiency by normalizing to % BFP positive cells.

Fig S15

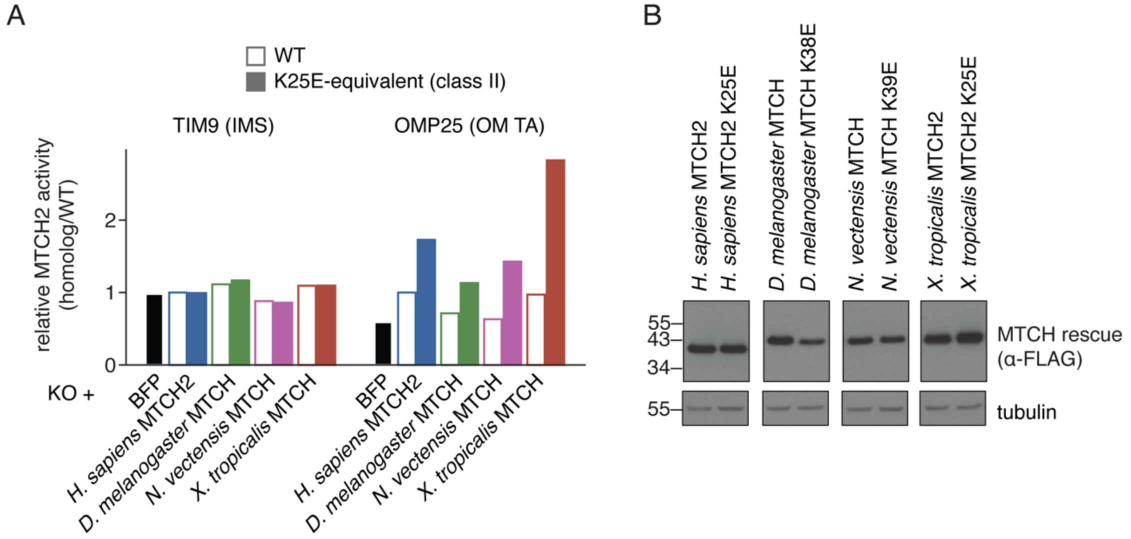

Fig. S15. Activity of the MTCH homologs are all attenuated through a similar mechanism. **A)** To test if the activity of the MTCH family of insertases relied on a similar mechanism to *H. sapiens* MTCH2, we introduced the equivalent mutation to the activating K25E to several MTCH homologs. The ratiometric reporter system was used to measure the insertion of indicated OM proteins and a soluble IMS control. Rescue constructs encoding a BFP control, WT MTCH2, and K25E-equivalent mutations to 3 MTCH homologs from *D. melanogaster, N. vectensis*, and *X. tropicalis*, all 3xFLAG-tagged, were expressed to determine their effect on OM insertion. Relative insertion of each construct was compared to WT MTCH2 (calculated as median GFP:RFP$_{mut}$/median GFP:RFP$_{wt}$). K25E-equivalent mutations were activating across all homologs tested, suggested a conserved mechanism of activity across all MTCH homologs. Additional data for this experiment is displayed in Fig. 3E. **B)** Immunoblot analysis was performed to confirm the expression of the MTCH homologs and the MTCH homolog mutants shown in (A). Individual lanes were cropped from the same immunoblot at the same exposure level.

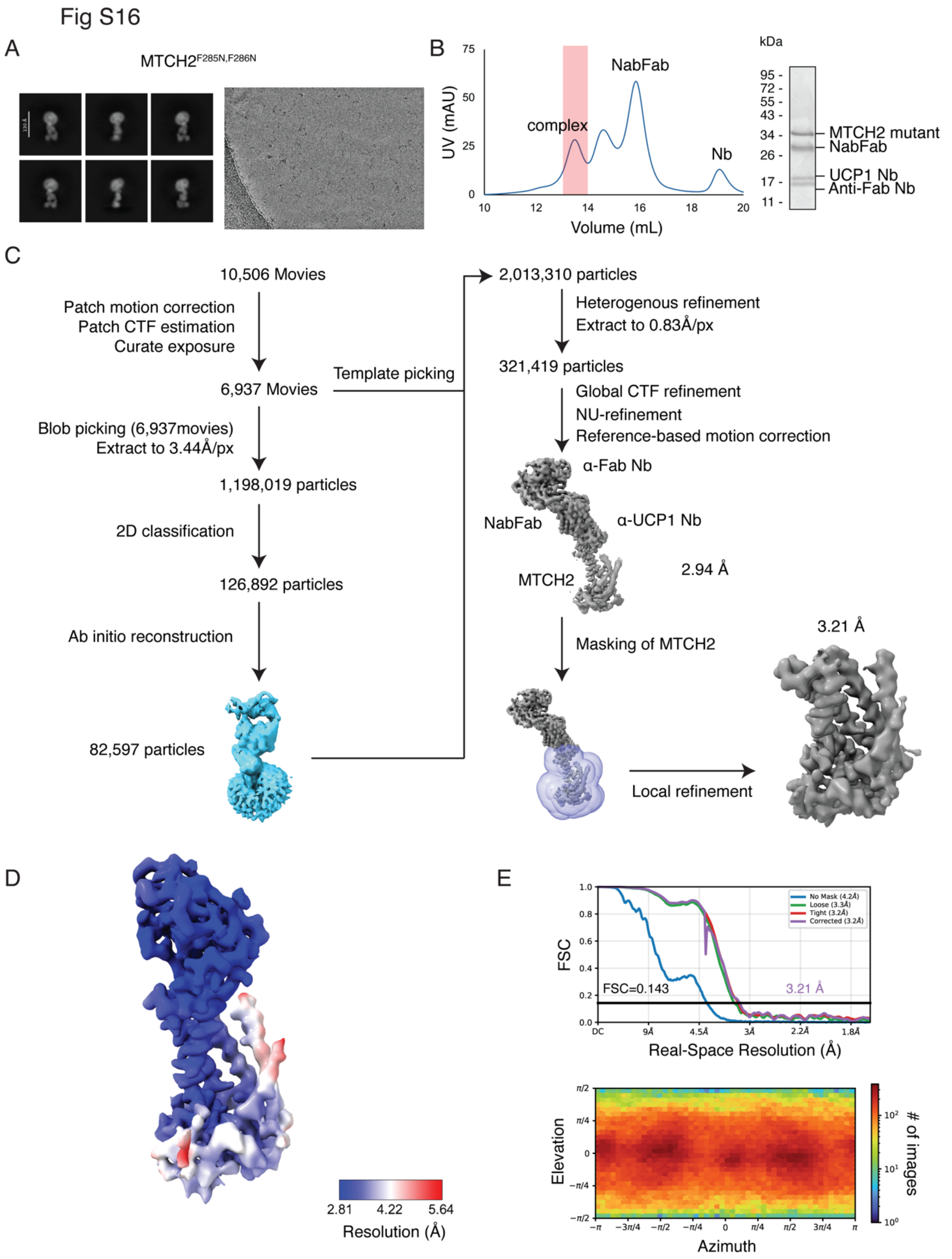

Fig. S16. Cryo-EM data processing workflow for MTCH2^{F285N,F286N}. A) (left) Representative 2D class averages of the MTCH2^{F285N,F286N}•α-UCP1 Nb•NabFab•α-Fab Nb complex, (right) and a micrograph from the data collection. **B)** (left) Size exclusion chromatography trace with the

peak corresponding to the MTCH2^{F285N,F286N}•α-UCP1 Nb•NabFab•α-Fab Nb complex highlighted in red. (right) Pooled peak fractions were analyzed by SDS-PAGE and SYPRO Ruby stain (right). **C)** Data processing pipeline of MTCH2^{F285N,F286N} using cryoSPARC v4.7.0. Micrographs were curated and a subset was used for blob picking. The resulting particle set was extracted at 3.44 Å/pixel and subject to iterative rounds of 2D classification. Particles from selected 2D classes were used in ab-initio reconstruction to generate 3D volumes. The volume representing a Fab attached to a detergent micelle was selected and used to generate 2D templates for template picker. After re-picking particles from the entire dataset, the resulting particle set was combined with the initial set and processed through iterative rounds of heterogenous refinement, yielding a final particle set of 321,419 particles. The particles were re-extracted at 0.83 Å/pixel and subject to global CTF refinement and reference-based motion correction. The final map was obtained from a non-uniform refinement job followed by the local refinement job using a mask on MTCH2 only. **D)** Cryo-EM density map of MTCH2^{F285N,F286N}•α-UCP1 Nb colored by local resolution in Å as calculated by cryoSPARC v4.7.0. **E)** Gold Standard Fourier Shell Correlation (GSFSC) curves (top) of the MTCH2 complex with a loose mask (green), tight mask (red), corrected mask (purple) or no mask (blue), and particle Euler angle distribution (bottom). A nominal resolution of 3.21 Å was determined for this map based off of an FSC cutoff of 0.143. Both plots were generated from the local refinement job in cryoSPARC v4.7.0.

Fig S17

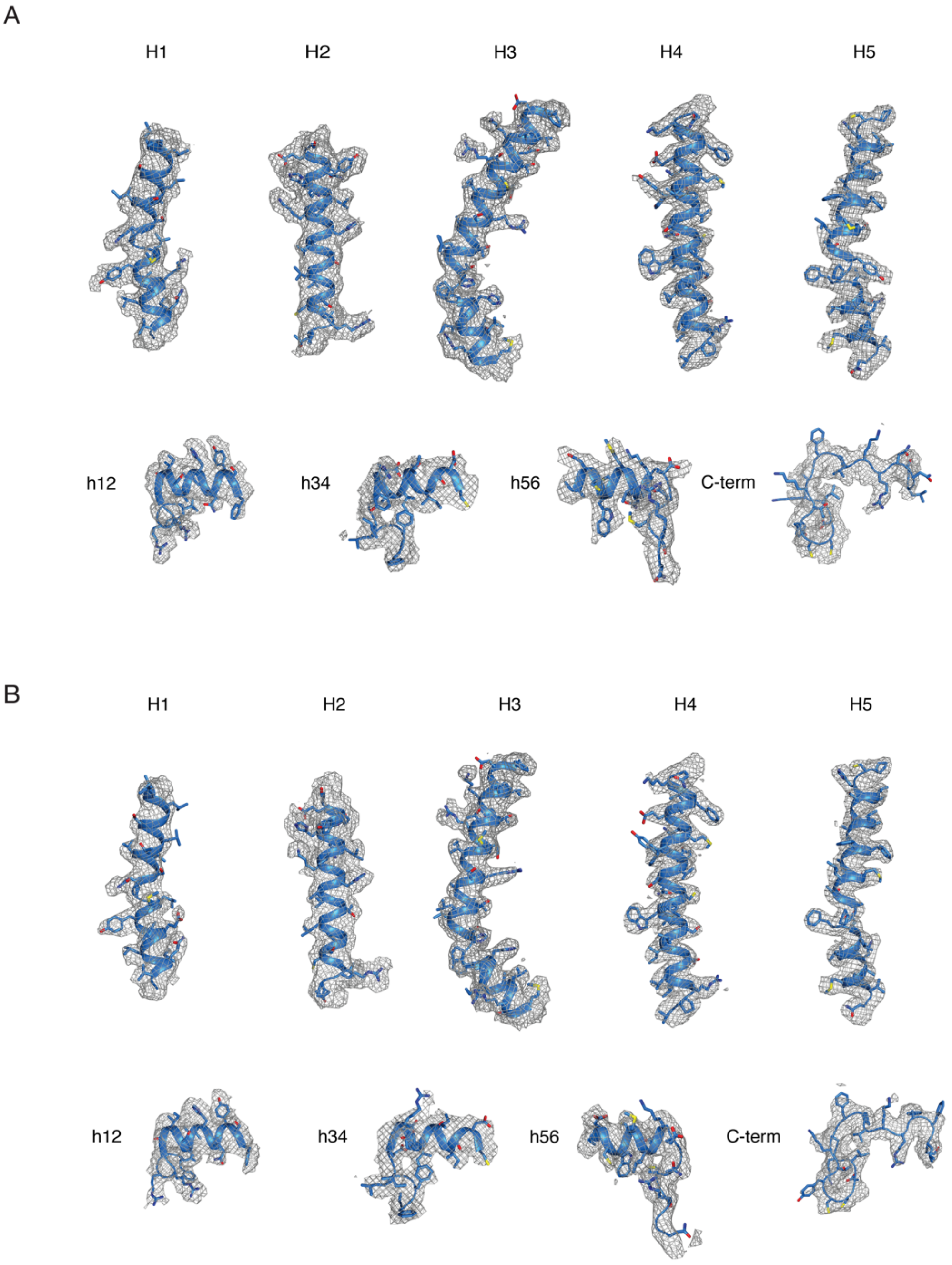

Fig. S17. Representative density for the MTCH2^{K25E,Y235A,V238D} and MTCH2^{F285N,F286N} mutant structures. A) Mesh density from the MTCH2^{K25E,Y235A,V238D} cryo-EM map displayed around the 5 TM helices, helical linker h12, h34, h56, and the C-terminus, highlighting overall map quality, with density for at least one or two prominent side chains visible on each helix. **B)** Same as (A) for MTCH2^{F285N,F286N}.

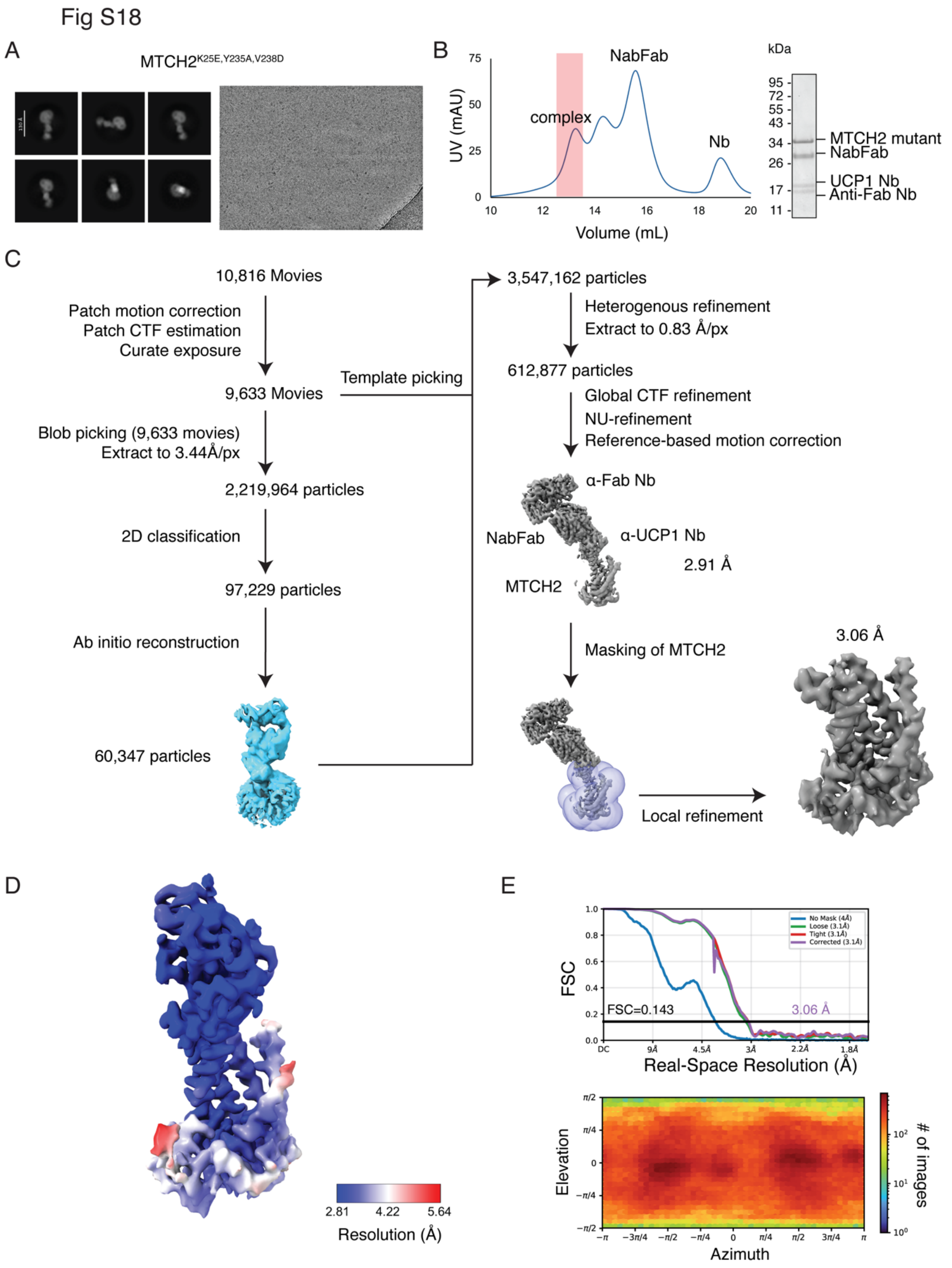

Fig. S18. **Cryo-EM data processing workflow for MTCH2^{K25E,Y235A,V238D}. A)** (left) Representative 2D class averages of the MTCH2^{K25E,Y235A,V238D}•α-UCP1 Nb•NabFab•α-Fab Nb complex, (right) and a micrograph from the data collection. **B)** (left) Size exclusion

chromatography trace with the peak corresponding to the MTCH2^{K25E,Y235A,V238D}•α-UCP1 Nb•NabFab•α-Fab Nb complex highlighted in red. (right) Pooled peak fractions were analyzed by SDS-PAGE and SYPRO Ruby stain (right). **C)** Data processing pipeline of MTCH2^{K25E,Y235A,V238D}, same as that for MTCH2^{F285N,F286N}•α-UCP1 Nb•NabFab•α-Fab Nb complex described in S15C, resulting in a set of 612,877 particles. **D)** Cryo-EM density map of MTCH2^{K25E,Y235A,V238D}•α-UCP1 Nb colored by local resolution in Å as calculated by cryoSPARC v4.7.0. **E)** Gold Standard Fourier Shell Correlation (GSFSC) curves (top) of the MTCH2 complex with a loose mask (green), tight mask (red), corrected mask (purple) or no mask (blue), and particle Euler angle distribution (bottom). A nominal resolution of 3.06 Å was determined for this map based off of an FSC cutoff of 0.143. Both plots were generated from the local refinement job in cryoSPARC v4.7.0.

Fig S19

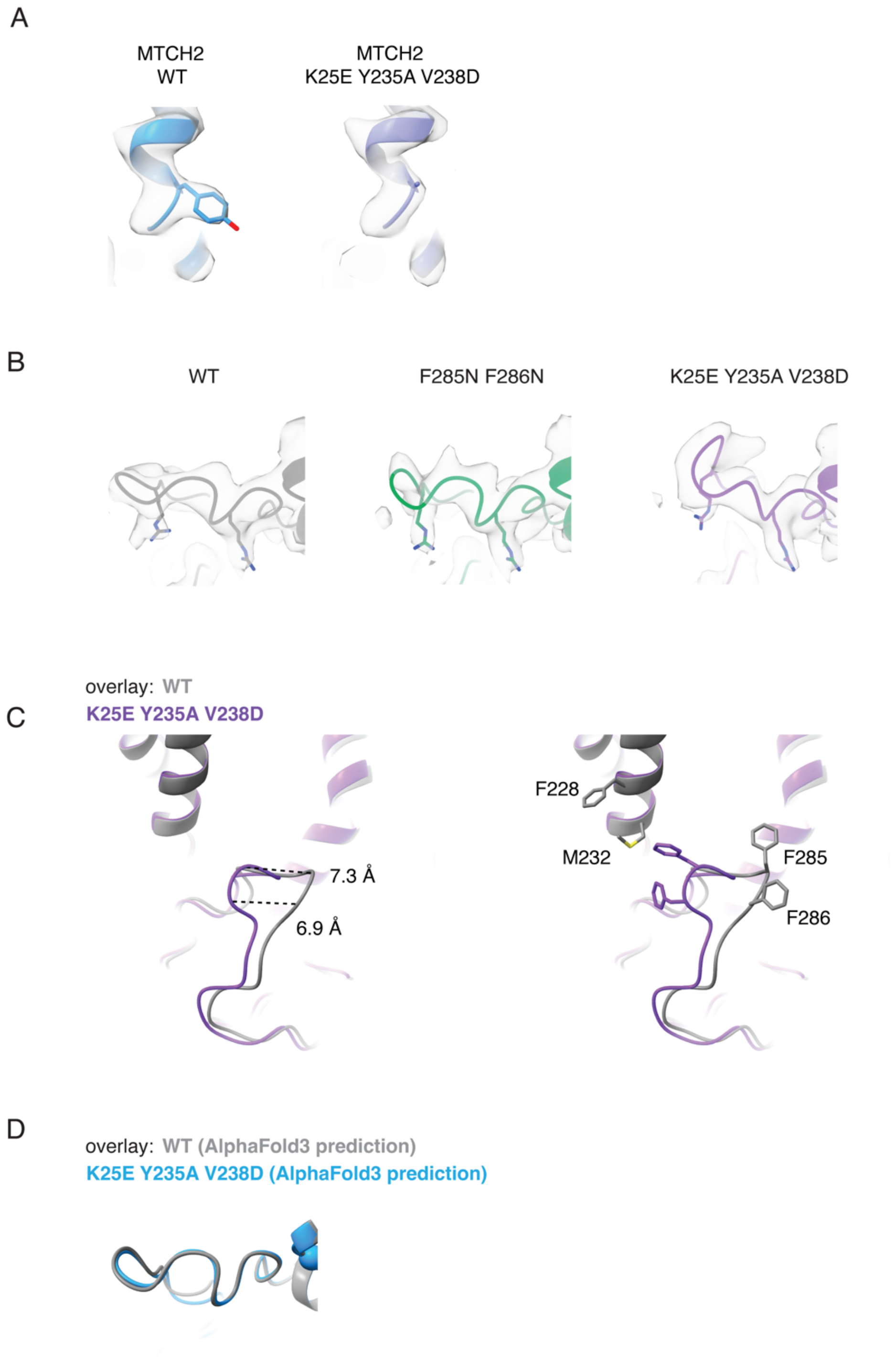

Fig. S19. Cryo-EM density of the alternative C-terminal conformation in MTCH2^{K25E,Y235A,V238D}. A) Comparison of the cryo-EM density surrounding Y235 in WT MTCH2 and MTCH2^{K25E,Y235A,V238D} confirms the loss of density due to the alanine mutation. **B)** Comparison of cryo-EM density around the C-terminus for WT MTCH2, MTCH2^{K25E,Y235A,V238D},

and MTCH2^{F285N,F286N}. Prominent density for two arginine residues, R280 and R287, help to verify the correct sequence register and overall positioning of the peptide backbone for the region. **C)** (left) C-terminus centered view of MTCH2 WT (gray) overlayed with MTCH2^{K25E,Y235A,V238D} (purple), with measurements of the Cα displacement shown for F285 and F286. (right) The same overlay with side chains for F228, M232, F285, and F286 for WT and the altered F285 and F286 positions for MTCH2^{K25E,Y235A,V238D}. **D)** Overlay of the C-terminus of the AlphaFold3-predicted models of WT MTCH2 and MTCH2^{K25E,Y235A,V238D}, showing no conformational change.

Fig S20

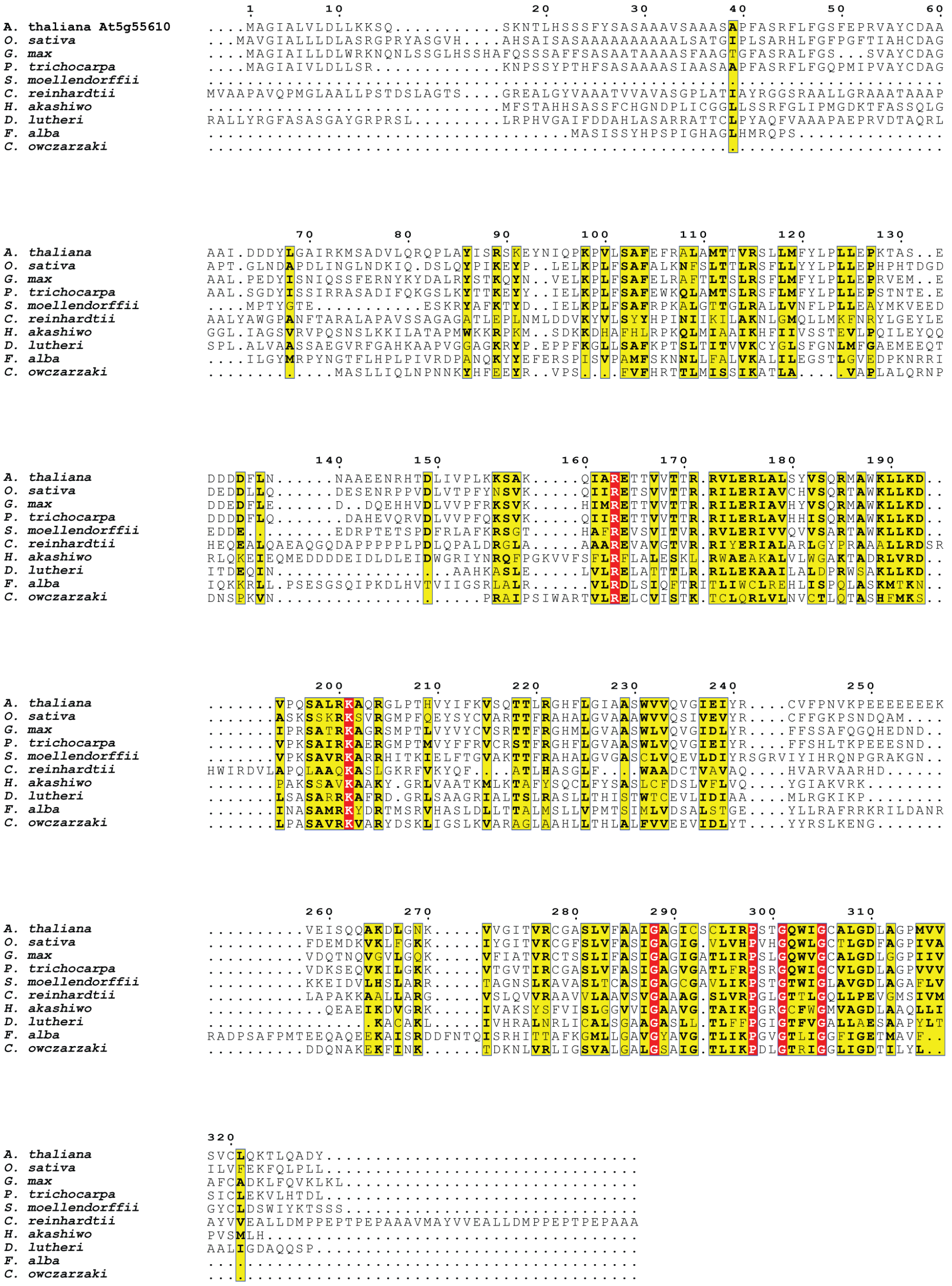

Fig. S20. The At5g55610 sequence is conserved across plants and other eukaryotes. Multiple sequence alignment of At5g55610 homologs from plants, *C. reinhardtii* (Chlorophyte), *H. akashiwo* (stramenopiles), *D. lutheri* (haptista), *C. owczarzaki* (holozoa), and *F. alba*

(holomycota) generated with MUSCLE (*75*) and visualized with the ESPript 3.2 server (*76*). The yellow regions correspond to positions where 70% or more of the residues have similar physicochemical properties, and the red regions correspond to positions where all residues have similar physicochemical properties

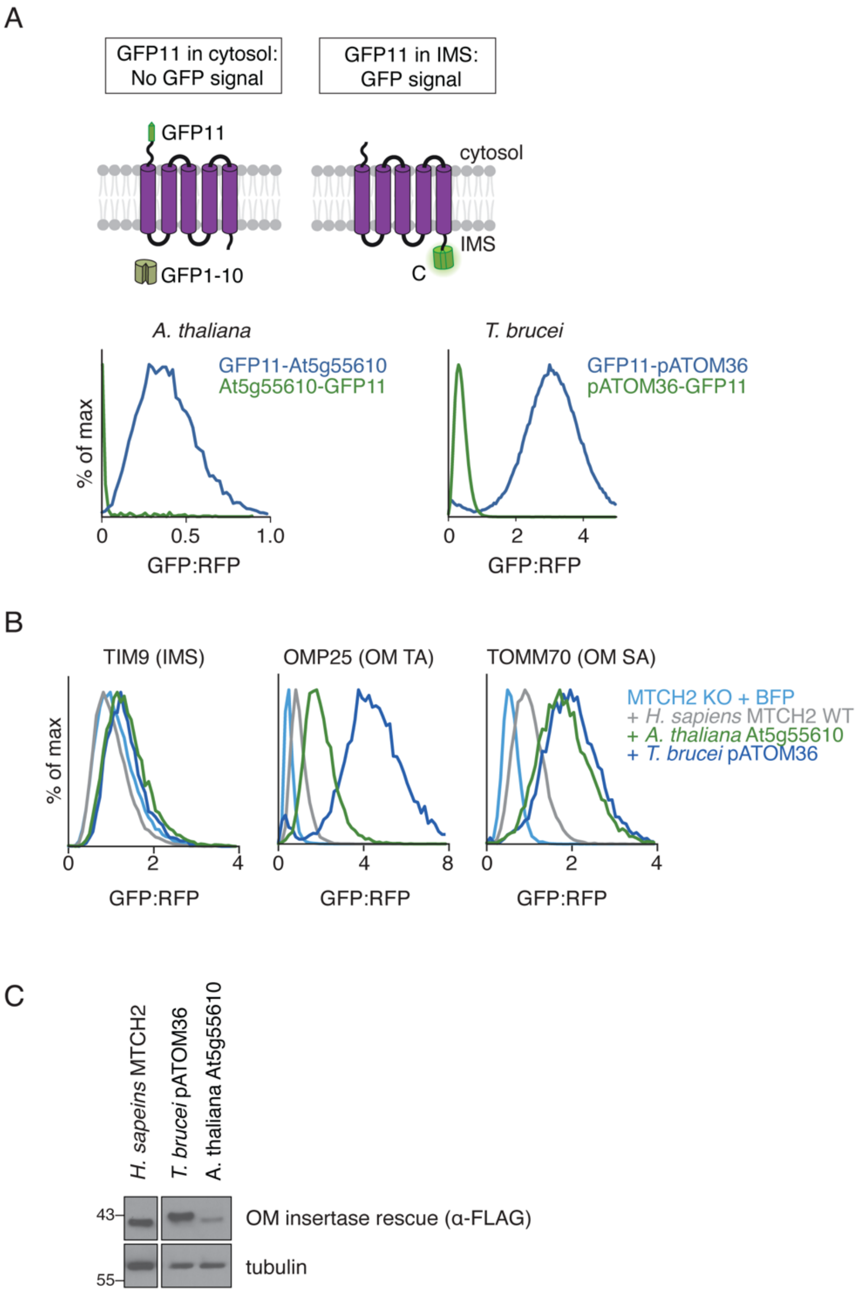

Fig. S21. pATOM36 and At5g55610 localize to the OM and function as insertases in human cells. A) Flow cytometry measurements of K562 IMS GFP(1-10) cells expressing RFP-P2A-tagged pATOM36 and At5g55610 with GFP11 on either the N- (blue) or C-terminus (green). N-terminal GFP11 tags have a much higher GFP:RFP ratio for both proteins, indicating that the N-terminus is in the IMS, while the C-terminus is in the cytosol. This is a reverse topology compared to MTCH homologs, and when analyzed together with the AlphaFold3 models (shown in Fig. 5B) suggests that pATOM36 and At5g55610 adopt an inverted fold compared to the

MTCH homolog family. AlphaFold modeling suggested that these plant and protist OM insertases adopt an orientation that is more open towards the cytosol to facilitate their insertase function, similar to that observed for MTCH2.**B)** Additional data corresponding to Fig. 5C. We compared the insertase activity in human cells of MTCH2 to the known OM insertase from *T. brucei* pATOM36 and an OM protein from *A. thaliana* At5g55610 identified for its resemblance to MTCH2 and especially pATOM36. We used our ratiometric reporter assay to query indicated OM substrates and an IMS control. Rescue constructs encoding a BFP control, WT MTCH2, pATOM36, and At5g55610, all 3x-FLAG tagged, were expressed to determine their effect on OM insertion. Results are displayed as a histogram, normalized to WT MTCH2. **C)** Immunoblot analysis of was performed to compare expression levels of MTCH2 with At5g55610 and pATOM36 rescue constructs used in (A). Individual lanes were cropped from the same immunoblot at the same exposure level.

Fig S22

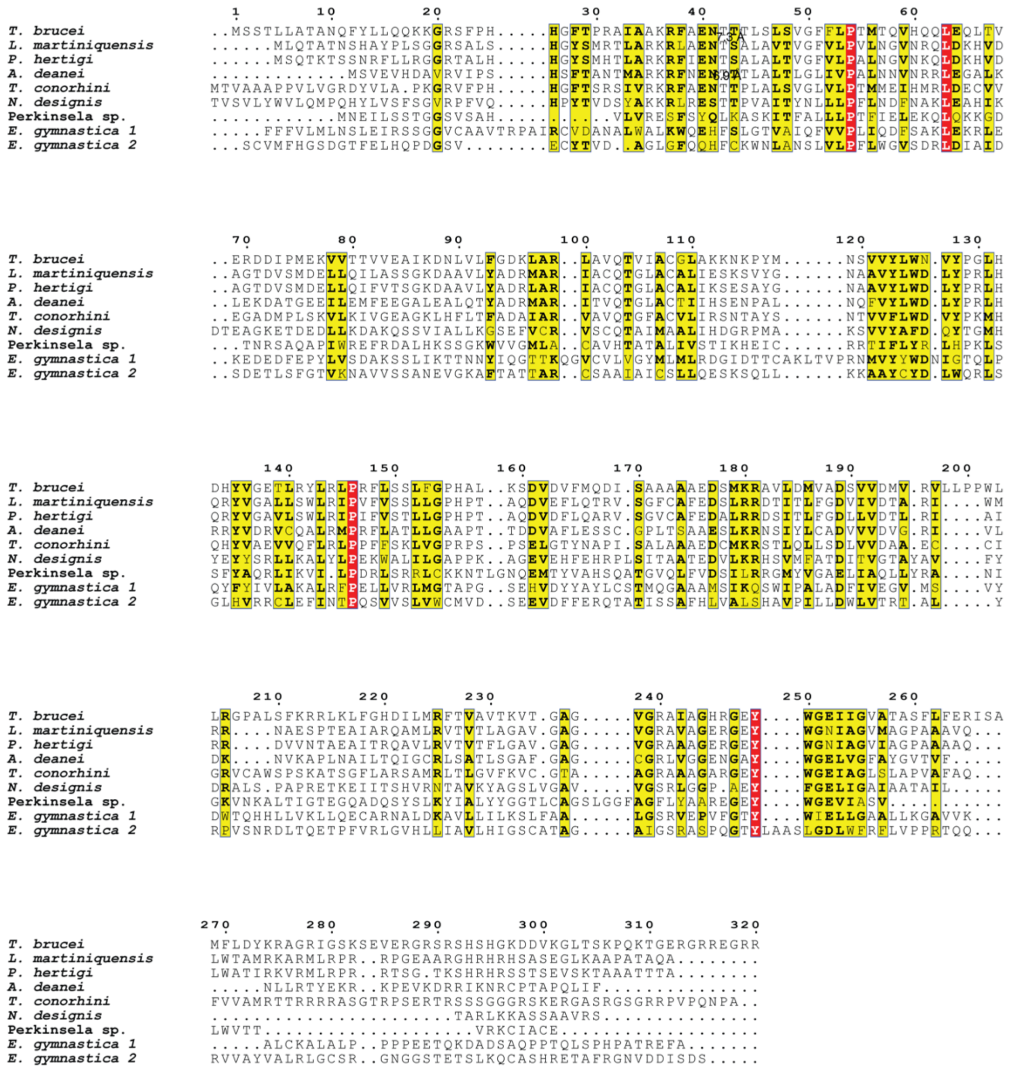

Fig. S22. The pATOM36 sequence is conserved across Euglenozoa. Multiple sequence alignment of pATOM36 homologs from Euglenozoa generated with MUSCLE (*75*) and visualized with the ESPript 3.2 server (*76*). The yellow regions correspond to positions where 70% or more of the residues have similar physicochemical properties, and the red regions correspond to positions where all residues have similar physicochemical properties.

Table S1. Cryo-EM data collection, refinement and validation statistics

	MTCH2 (BRIL)	MTCH2 (UCP1)	MTCH2 F285N F286N	MTCH2 K25E Y235A V238D
**Data collection and processing**				
Magnification (nominal)	105,000	105,000	105,000	105,000
Voltage (kV)	300	300	300	300
Electron exposure (e–/Å^2)	60	60	60	60
Defocus range (µm)	-0.8 to -1.8	-0.8 to -1.8	-0.8 to -1.8	-0.8 to -1.8
Pixel size (Å)	0.832	0.832	0.832	0.832
Initial particle images (no.)	7,786,491	4,791,546	3,547,162	2,013,310
Final particle images (no.)	199,397	485,622	612,877	321,419
Map resolution (Å)	3.55	3.57	3.06	3.21
FSC threshold	0.143	0.143	0.143	0.143
Map resolution range (Å)	3.0-9.8	2.9-7.4	2.5-8.5	2.7-7.4
**Refinement**				
Software (phenix.real_space_refine)	N/A	PHENIX 1.21.2-5419-000		
Initial model used (PDB code)	AlphaFold3	AlphaFold3	AlphaFold3	AlphaFold3
Correlation coefficient (CC_{mask})		0.85	0.83	0.83
Map sharpening *B* factor (Å^2)		0	0	0
Model composition				
Non-hydrogen atoms		3301	3295	3295
Protein residues		426	426	426
B factors (Å^2)				
Protein (min/max/mean)		71/265/136	67/340/177	80/355/194
R.m.s. deviations				
Bond lengths (Å)		0.002	0.003	0.002
Bond angles (°)		0.477	0.406	0.444
Validation				
MolProbity score		1.24	1.22	1.32
Clashscore		4.25	2.74	3.80
Poor rotamers (%)		0.56	0.85	0.56
Cβ outliers (%)		0.00	0.00	0.00
CaBLAM outliers (%)		1.44	2.15	1.67
Ramachandran plot				
Favored (%)		97.87	97.16	97.16
Allowed (%)		2.13	2.84	2.84
Disallowed (%)		0.00	0.00	0.00

Table S2. Plasmids used

**Plasmid**	**Backbone**	**Insert**	**Source**
		Split GFP-reporter plasmids	
pAG391	UCOE EF-1α	RFP-P2A-MAVS-GFP11	Guna et al. (*8*)
pAG397	UCOE EF-1α	RFP-P2A-OMP25-GFP11	Guna et al. (*8*)
pAG205	UCOE EF-1α	RFP-P2A-MICU1(1-60)-GFP11-Sec61B	Guna et al. (*8*)
pAG206	UCOE EF-1α	RFP-P2A-TIM9A-GFP11	Guna et al. (*8*)
pMH409	UCOE EF-1α	RFP-P2A-RHOT2-GFP11	Guna et al. (*8*)
pAG435	UCOE EF-1α	RFP-P2A-FUNDC1-GFP11	Guna et al. (*8*)
pAG648	UCOE EF-1α	RFP-P2A-GFP11-TOMM70	Guna et al. (*8*)
pTS311	UCOE EF-1α	RFP-P2A-GFP11-At5g55610-3xFLAG	this study
pTS702	UCOE EF-1α	RFP-P2A-3xFLAG-At5g55610-GFP11	this study
pTS331	UCOE EF-1α	RFP-P2A-ATOM36-GFP11	this study
pAG658	UCOE EF-1α	RFP-P2A-GFP11-ATOM36-1xFlag	this study
pAG670	UCOE EF-1α	RFP-P2A-C. owczarzaki MTCH2-GFP11	this study
pTS686	UCOE EF-1α	RFP-P2A-GFP11-C. owczarzaki MTCH2	this study
pTS684	UCOE EF-1α	RFP-P2A-MTCH1-L-GFP11 (Q9NZJ7-1)	this study
pTS685	UCOE EF-1α	RFP-P2A-GFP11-MTCH1-L (Q9NZJ7-1)	this study
pAG553	UCOE EF-1α	RFP-P2A-GFP11-MTCH2	this study
pAG554	UCOE EF-1α	RFP-P2A-MTCH2-GFP11	Muthukumar et al. (*22*)
pAG708	UCOE EF-1α	RFP-1xFLAG-M. musculus MTCH2-GFP11	this study
pAG676	UCOE EF-1α	RFP-1xFLAG-X. tropicalis MTCH2-GFP11	this study
pAG673	UCOE EF-1α	RFP-1xFLAG-D. melanogaster MTCH2-GFP11	this study
pAG674	UCOE EF-1α	RFP-1xFLAG-N. vectensis MTCH2-GFP11	this study
		Mammalian rescue plasmids	
pAG496	UCOE EF-1α	BFP-P2A-MTCH2	Guna et al. (*8*)
pAG301	UCOE EF-1α	BFP	Guna et al. (*8*)
pTS682	UCOE EF-1α	BFP-P2A-MTCH2 (UCP1)	this study
pTS683	UCOE EF-1α	BFP-P2A-MTCH2 (BRIL)	this study
pAG718	UCOE EF-1α	BFP-P2A-3xFlag-C. owczarzaki MTCH	this study
pCL21	UCOE EF-1α	BFP-P2A-3xFlag-C. owczarzaki MTCH K35E F324A S327D	this study
pAG719	UCOE EF-1α	BFP-P2A-3xFlag-D. melanogaster MTCH	this study
pCL22	UCOE EF-1α	BFP-P2A-3xFlag-D. melanogaster MTCH K38E Y241A Q244D	this study
pAG720	UCOE EF-1α	BFP-P2A-3xFlag-N. vectensis MTCH	this study
pCL23	UCOE EF-1α	BFP-P2A-3xFlag-N. vectensis MTCH K39E Y247A V250D	this study
pAG721	UCOE EF-1α	BFP-P2A-3xFLAG-X. tropicalis MTCH2	this study
pAG710	UCOE EF-1α	BFP-P2A-3xFLAG-M. musculus MTCH2	this study
pAG570	UCOE EF-1α	BFP-P2A-MTCH1-L (Q9NZJ7-1)	this study
pCL16	UCOE EF-1α	BFP-P2A-MTCH1-S (Q9NZJ7-2)	this study
pAG569	UCOE EF-1α	BFP-P2A-SLC25A46	this study
		Lentiviral packaging plasmids	
psPax2			gift from Didier Trono (Addene #12260)
pMD2.G			gift from Didier Trono (Addene #12259)
		Baculovirus generation plasmids	

pZL5	pFastBac1	GFP-SUMO-MTCH2(BRIL fusion)	this study
pZL10	pFastBac1	GFP-SUMO-MTCH2(UCP1 fusion)	this study
pZL15	pFastBac1	GFP-SUMO-MTCH2(UCP1 fusion) F285N F286N	this study
pZL16	pFastBac1	GFP-SUMO-MTCH2(UCP1 fusion) K25E Y235A V238D	this study
		E. coli expression plasmids	
pTS117	pQE	14xHis-avi-GFP Nb	Stevens et al. (*68*) (Addgene #199370)
pAV286	pDG	SENPEu	Vera Rodriguez et al. (*69*) (Addgene #149333)
pTS534	pET Duet	Pelb-14xHis-TEV-anti-UCP1 Nb TC-NB4 scaffold	This study
pZL2	pET Duet	Pelb-14xHis-TEV-anti-Fab Nb	Li et al. (*92*)
	pRH2.2	NabFab	Bloch et al. (*30*)
	pRH2.2	anti BRIL sAB BAK5	Mukherjee et al. (*28*)
		In vitro translation plasmids	
pAI351	SP64	OMP25	Guna et al. (*8*)
pAI379	SP64	VHP-RHOT1	Guna et al. (*8*)
gTS10	SP64	SU9-DHFR	Guna et al. (*8*)

Table S3. CRISPRi sgRNA plasmids used

**Plasmid**	**Backbone**	**sgRNA**	**sgRNA sequence**	**Source**
pAG285	pU6-sgRNA EF-1α-Puro	Non-targeting	GAACGACTAGTTAGGCGTGTA	Guna et al. (*8*)
pAG59	pU6-sgRNA EF-1α-Puro-T2A-BFP	Non-targeting	GAACGACTAGTTAGGCGTGTA	Guna et al. (*8*)
pAG491	pU6-sgRNA-hU6-sgRNA EF-1α-Puro	MTCH2 dual	GACCGGCTCACCGGGTCGCT, GGGCTCACCGGGTCGCTTGG	Guna et al. (*8*)
pAG692	pU6-sgRNA-hU6-sgRNA EF-1α-Puro	MTCH1 dual	GCGGCACCGCCGCGAGCCCA, GAGCCCAGGGCGCCACTTCC	Guna et al. (*8*)
pAG515	pU6-sgRNA-hU6-sgRNA EF-1α-Puro-T2A-BFP	MTCH1 dual	GCGGCACCGCCGCGAGCCCA, GAGCCCAGGGCGCCACTTCC	Guna et al. (*8*)
pAG440	pU6-sgRNA-hU6-sgRNA EF-1α-Puro-T2A-BFP	MTCH2 single	GACGGAGCCACCAAGCGACC	Guna et al. (*8*)
pAG514	pU6-sgRNA-hU6-sgRNA EF-1α-Puro-T2A-BFP	SLC25A46 dual	GCAGGGATGAGGGGTTACTG, GAGCACACACAGCTTCCCGT	this study
pMH458	pU6-sgRNA EF-1α-Puro-T2A-BFP	ARMC1 #1	GGGAAGCGGCCCCTGTACCG	this study
pMH459	pU6-sgRNA EF-1α-Puro-T2A-BFP	ARMC1 #2	GCAGAGGCGTCAGGTCACCT	this study
pAG452	pU6-sgRNA-hU6-sgRNA EF-1α-Puro-T2A-BFP	DNAJC11 dual	GTGTCCCTGACGCGGATCAC, GGGGCCAGTGATCCGCGTCA	this study

Table S4. UniProtKB-based MTCH homolog identification

Protein family	PTHR10780:SF20 (MTCH2)	PTHR10780:SF3 (MTCH1)	PTHR10780:SF18 (MTCH)
Species		# of sequences	
H. sapiens (human)	4	23	0
M. musculus (mouse)	5	9	0
X. tropicalis (frog)	4	0	0
S. salar (salmon)	3	3	0
C. elegans (nematode)	0	0	2
D. melanogaster (fly)	0	0	14
N. vectensis (anenome)	0	0	3
S. rosetta (choanoflagellate)	0	0	1
C. owczarzaki	0	0	1
S. arctica	0	0	1

Table S5. UniProtKB-based OM insertase homolog identification

Protein family	PTHR10780 (MTCH)	PTHR28241 (Mim1)	IPR043645 (pATOM36)	PTHR36074 (At5g55610)
Clade (taxid)	# of sequences			
Euglenozoa (33682)	0	0	42	0
Heterolobosea (5752)	0	0	0	0
Chlorophyta (3041)	0	0	0	45
Streptophyta (35493)	0	0	0	682
Rhodophyta (2763)	0	0	0	0
Alveolata (33630)	0	0	0	0
Stramenopila (33634)	0	0	0	94
Amoebozoa (554915)	0	0	0	0
Fungi (4751)	0	1235	0	0
Fonticula (691882)	0	0	0	1
Capsaspora (192874)	0	0	0	1
Choanoflagellates (28009)	2	0	0	0
Metazoans (33208)	2836	0	0	0

Movie S1. Morph between WT MTCH2 and $MTCH2^{K25E,Y235A,V238D}$ conformation shown in space-filling representation.
